# Ring Enlargement of Three‐Membered Heterocycles by Treatment with In Situ Formed Tricyanomethane†

**DOI:** 10.1002/chem.202000089

**Published:** 2020-04-30

**Authors:** Klaus Banert, Madhu Chityala, Marcus Korb

**Affiliations:** ^1^ Organic Chemistry Chemnitz University of Technology Strasse der Nationen 62 09111 Chemnitz Germany; ^2^ Faculty of Science School of Molecular Sciences The University of Western Australia 35 Stirling Highway Crawley, Perth, Western Australia 6009 Australia

**Keywords:** cyanoform, nitrogen heterocycles, reaction mechanisms, reactive intermediates, ring expansion

## Abstract

Although the chemistry of elusive tricyanomethane (cyanoform) has been studied during a period of more than 150 years, this compound has very rarely been utilized in the synthesis or modification of heterocycles. Three‐membered heterocycles, such as epoxides, thiirane, aziridines, or 2*H*‐azirines, are now treated with tricyanomethane, which is generated in situ by heating azidomethylidene‐malonodinitrile in tetrahydrofuran at 45 °C or by adding sulfuric acid to potassium tricyanomethanide. This leads to ring expansion with formation of 2‐(dicyanomethylidene)oxazolidine derivatives or creation of the corresponding thiazolidine, imidazolidine, or imidazoline compounds and opens up a new access to these push–pull‐substituted olefinic products. The regio‐ and stereochemistry of the ring‐enlargement processes are discussed, and the proposed reaction mechanisms were confirmed by using ^15^N‐labeled substrates. It turns out that different mechanisms are operating; however, tricyanomethanide is always acting as a nitrogen‐centered nucleophile, which is quite unusual if compared to other reactions of this species.

## Graphical abstract

**Hidden acid**: Cyanoform, which is generated in situ from azidomethylidene‐malonodinitrile or other precursors, induces ring‐expansion reactions of epoxides, thiirane, aziridines, or 2*H*‐azirines. The transformations are always initiated through protonation of the heteroatom X by cyanoform, but lead to the five‐membered heterocycles through different mechanisms (see scheme).

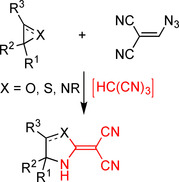

## Introduction

**Scheme 1:**
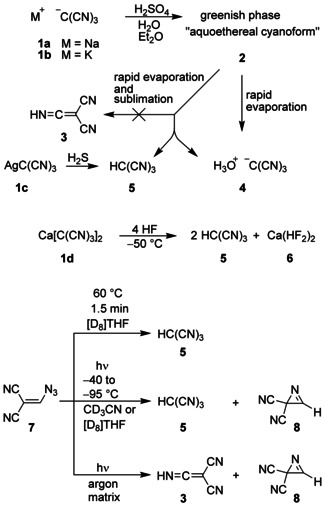
Previous syntheses of tricyanomethane (**5**).

The history of tricyanomethane (**5**) dates back to 1864 when the first isolation of so‐called cyanoform was reported.^1^ But this and a second synthesis^2^ could not be reproduced by other chemists,^2^, ^3^ and later it was found that **5**, even if generated, would not have been able to survive the described drastic reaction conditions. In 1896, Schmidtmann treated sodium tricyanomethanide (**1 a**) with dilute sulfuric acid and then with diethyl ether (Scheme 1).^4^ He obtained a three‐phase system, which comprised a greenish yellow middle layer **2** that was claimed to include cyanoform (**5**), although first experiments to remove the solvents did not lead to characterizable substances. Three years later, Hantzsch and Osswald confirmed these phenomena and stated that tautomerism of **5** is likely to form ketenimine **3**.^5^ In 1963, Trofimenko used the salt **1 b** to repeat the synthesis of **2** and called this compound “aquoethereal cyanoform”.^6^ He successfully liberated **2** from the solvents by rapid evaporation and vacuum sublimation to receive unstable white crystals, to which he erroneously assigned the structure of **3**. When Dunitz et al. performed the rapid evaporation of **2** without sublimation, they were able to isolate single crystals of hydronium tricyanomethanide (**4**), and that facilitated the structure verification by X‐ray diffraction studies.^7^ In 2017, it was shown that Trofimenko's experiment did not produce **3** because rapid evaporation and sublimation of **2** led to the isolation of a mixture of **4** and **5**.^8^ Under special conditions of vacuum sublimation, single crystals of tricyanomethane (**5**) were accessible, which allowed structure confirmation by X‐ray diffraction. If moisture was excluded, such single crystals could be handled at ambient temperature for a short time. But cyanoform (**5**) could not be analyzed in solution by NMR spectroscopy at room temperature due to rapid decomposition. Moreover, **5** was easily converted into **4** even if only trace amounts of water were present. Especially, mixtures of **4** and **5** tended to dynamic processes, which led to extremely broad ^1^H and ^13^C NMR signals at temperatures between −50 and 0 °C. Thus, even lower temperatures, for example, −70 °C, were necessary to detect sharp ^13^C NMR signals for pure **5** in [D_8_]THF.^8^

Cyanoform (**5**) was prepared not only from **1 a** or **1 b** via **2**, but also by reacting **1 c** with hydrogen sulfide,^9^ or alternatively by treatment of **1 d** with an excess of anhydrous hydrogen fluoride,^10^ and finally by short‐time thermolysis or photolysis of vinyl azide **7**.^8^ The products **5** and **6** could not be separated in the case of precursor **1 d**; however, convincing spectroscopic identification of **5** was nevertheless possible.^10^ Whereas thermolysis of **7** produced only **5** and small amounts of **4**, irradiation of **7** in solution led to **5** and the 2*H*‐azirine **8**.^8^ The photolysis of **7** isolated in an argon matrix did not generate **5** since ketenimine **3** and the heterocycle **8** were formed instead.

During a period of more than 150 years, the chemistry of cyanoform (**5**) has been studied thoroughly.^11^ Its gas‐phase structure was investigated by photoelectron spectroscopy^12^ and microwave spectroscopy.^9^ The relative stabilities, spectroscopic data, and isomerization reactions of **3**, **5**, and other C_4_HN_3_ species were analyzed by utilizing quantum‐chemical methods.^13^ In particular, the acidic properties of **5** as well as its tautomer **3** were discussed intensively.^5^, ^6^, ^14^ Hence, **5** is presented in textbooks of organic chemistry as one of the strongest carbon acids.

As a Brønsted acid, tricyanomethane (**5**) should be able to catalyze the ring opening of three‐membered heterocycles, for example, epoxides. If no other reactive species such as competing nucleophiles are present, interesting novel types of open‐chain or ring‐expanded products may result. Herein, we report on surprising transformations which were induced by treatment of oxiranes, thiirane, aziridines, and 2*H*‐azirines with in situ formed cyanoform (**5**).

## Results and Discussion

**Scheme 2:**
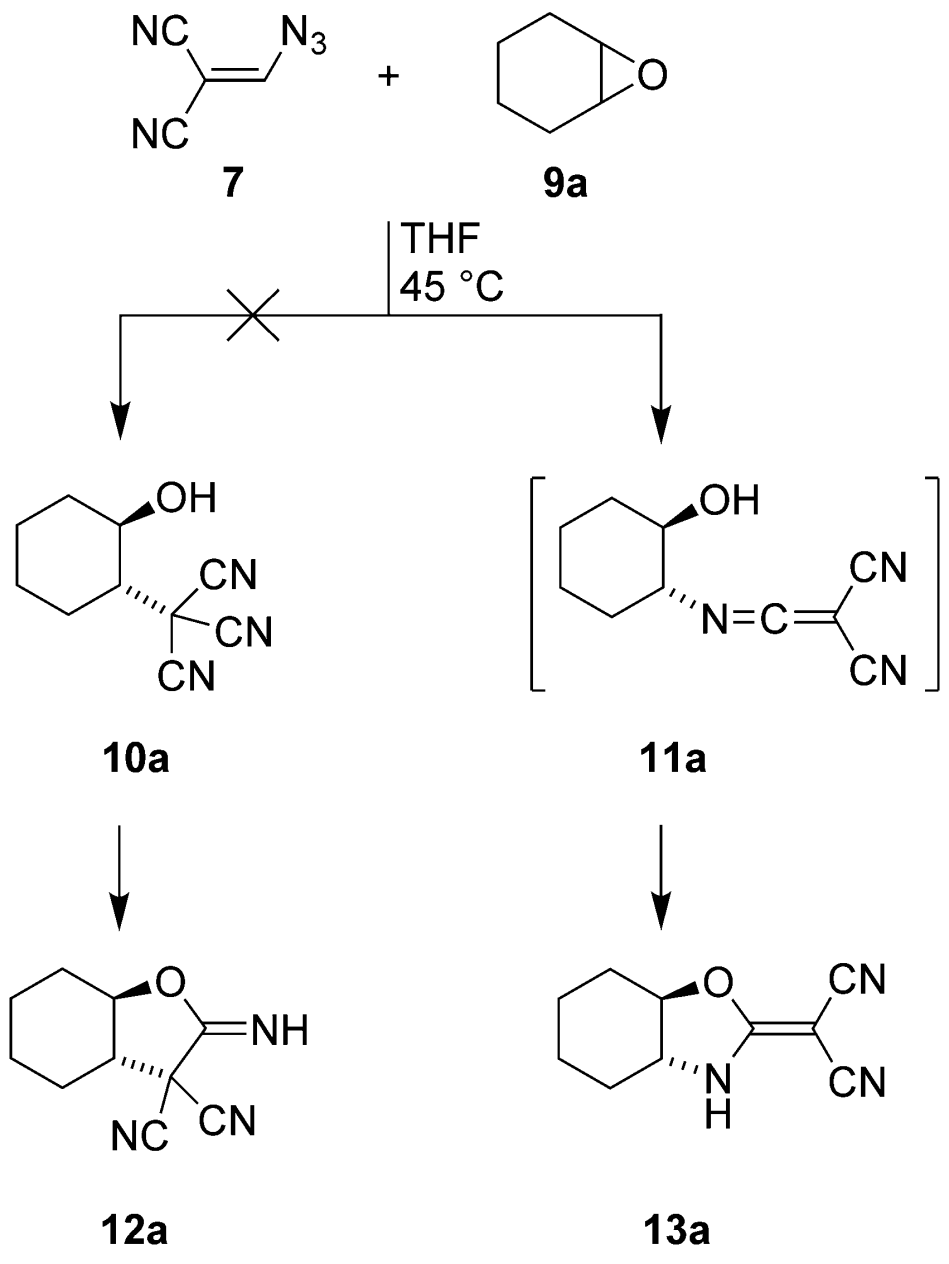
Ring enlargement of **9 a** by treatment with **7** and in situ generation of tricyanomethane (**5**).

At first, we utilized the commercially available cyclohexene oxide (**9 a**) as a substrate that was exposed to the precursor **7** in anhydrous THF at 45 °C (Scheme 2). We expected that **5** resulting from **7** would O‐protonate **9 a**, and the nucleophilic attack of the central carbon atom of the tricyanomethanide counterion would lead to the ring‐opened product **10 a**, which can possibly react to **12 a** by nucleophilic addition of the hydroxy group at a cyano unit. The former assumption was seemingly supported by the well‐known^15^ C‐alkylation of tricyanomethanide salts such as **1 b** with the help of alternative electrophiles,^16^, ^17^, ^18^ for example, primary alkyl halides. But instead of **10 a** or **12 a**, we obtained the isomeric product **13 a** after treatment of **9 a** with **7**. Obviously, protonated **9 a** was attacked by a nitrogen atom, and after ring opening, the resulting ketenimine **11 a** was transformed into the final product **13 a**, which includes a push–pull‐substituted C=C unit. Whereas both rings are *cis*‐fused in the starting compound **9 a**, the ^1^H NMR data of the ring‐expanded product **13 a** indicated a large vicinal coupling of the two axial bridgehead protons with ^3^ *J*=12.0 Hz. Consequently, both rings in **13 a** are *trans*‐fused.

Besides **9 a**, we similarly treated also the epoxides **9 b**–**h** with vinyl azide **7** to obtain the ring‐enlargement products **13** (Table 1). While the parent oxirane (**9 b**) exclusively led to the single product **13 b**,^19^ the reaction of propylene oxide (**9 c**) resulted in the formation of two regioisomeric oxazole derivatives, which were substituted with a methyl group in the 5‐ or in the 4‐position. Both isomers were easily separated by chromatography, and assignment by ^1^H and ^13^C NMR spectroscopy was simple since the adjacent oxygen atom always induced significantly stronger deshielding properties than the amine unit. In the case of styrene oxide (**9 d**), treatment with **7** regioselectively led to **13 d** as the sole product, and the analogous reaction of the unsymmetrical epoxide **9 e** similarly resulted in the exclusive formation of the oxazole derivative **13 e**, which was isolated in 79 % yield. Thus, the well‐known^20^ regiochemistry of acid‐catalyzed epoxide ring opening was also observed for the conversion of **9** into **13** although the involved reagents did not include an acid on the first view. However, slight warming of vinyl azide **7** generated the Brønsted acid cyanoform (**5**), which initiated the desired transformation by O‐protonation of **9**.

**Table 1:** Treatment of epoxides **9** with azide **7** to prepare the ring‐enlargement products **13**.

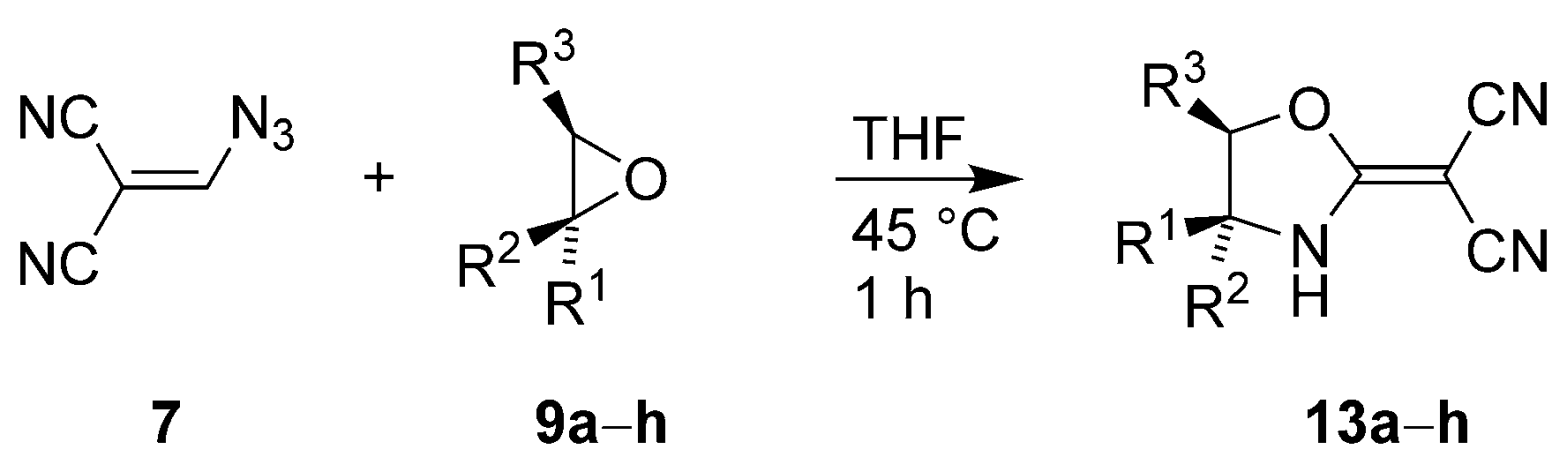							
	Substrate **9**			Product **13**			
	R^1^	R^2^	R^3^	R^1^	R^2^	R^3^	Yield [%]^]a]^
**a**	H	(CH_2_)_4_		H	(CH_2_)_4_		56
**b**	H	H	H	H	H	H	72
**c**	H	H	Me	H	H	Me	25
				Me	H	H	22
**d**	Ph	H	H	Ph	H	H	58
**e**	Ph	Ph	H	Ph	Ph	H	79
**f**	Ph	H	Bz	Ph	H	Bz	27
				H	Ph	Bz	26
**g**	H	Ph	Ph	H	Ph	Ph	46^[b]^
**h**	Ph	H	Ph	H	Ph	Ph	47^[b]^

[a] Isolated yields; after separation of two products in the case of **13 c** and **13 f**. [b] 1,2‐Diphenylethanone and diphenylacetaldehyde were additionally formed and isolated with a total yield of 13–14 %.

**Figure 1:**
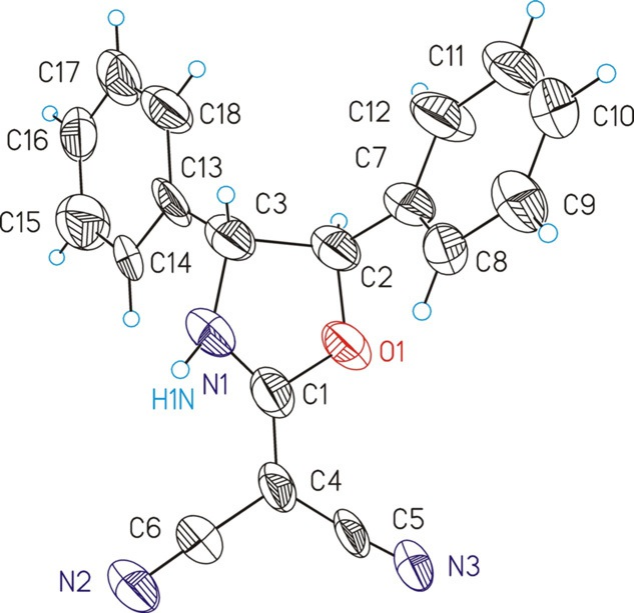
ORTEP (30 % ellipsoid probability) of the molecular structure of **13 g**. A second molecule in the asymmetric unit has been omitted for clarity. Selected bond properties ([Å]/[°]): C1−O1 1.45(3), C1−N1 1.31(3), C1−C4 1.37(3), O1−C2 1.45(3), C2−C3 1.60(3), C3−N1 1.43(2), C4−C5 1.40(3), C5−N3 1.15(3), C4−C6 1.43(3), C6−N2 1.21(3); N1‐C1‐O1 111(2), C1‐N1‐C3 114(2), C5‐C4‐C6 120(2), C7‐C2‐C3‐C13 128(2).

When the epoxide **9 f**, which includes a phenyl group and an electron‐withdrawing benzoyl substituent, was subjected to **7**, the product **13 f** was formed with perfect regioselectivity. However, **13 f** was composed of two stereoisomers isolated with nearly equal yields; the structural assignment of *cis*‐**13 f** and *trans*‐**13 f** was based on ^1^H NMR spectroscopy utilizing vicinal coupling constants and NOE experiments. In the case of *trans*‐4,5‐diphenyloxazole derivative **13 g/h**, the synthesis was successful starting with oxirane **9 g** or alternatively with the stereoisomer **9 h**. Thus, both reactions with **7** are stereoselective, but not stereospecific since the same product (**13 g=13 h**) was formed. The transformations of **9 g** and **9 h** were accompanied by the generation of 1,2‐diphenylethanone and diphenylacetaldehyde (total isolated yield: 13–14 %). These side products obviously resulted from ring opening of the O‐protonated epoxides followed by hydride shift or phenyl migration of the substituted benzyl cation. Similar carbocations were involved in the reactions of **9 d** or **9 e** with **7** which explains the regioselectivity in the formation of **13 d** and **13 e**, respectively. The structure of **13 g** was confirmed not only by the usual spectroscopic characterization, but also by single crystal X‐ray diffraction analysis (Figure 1).

**Scheme 3:**
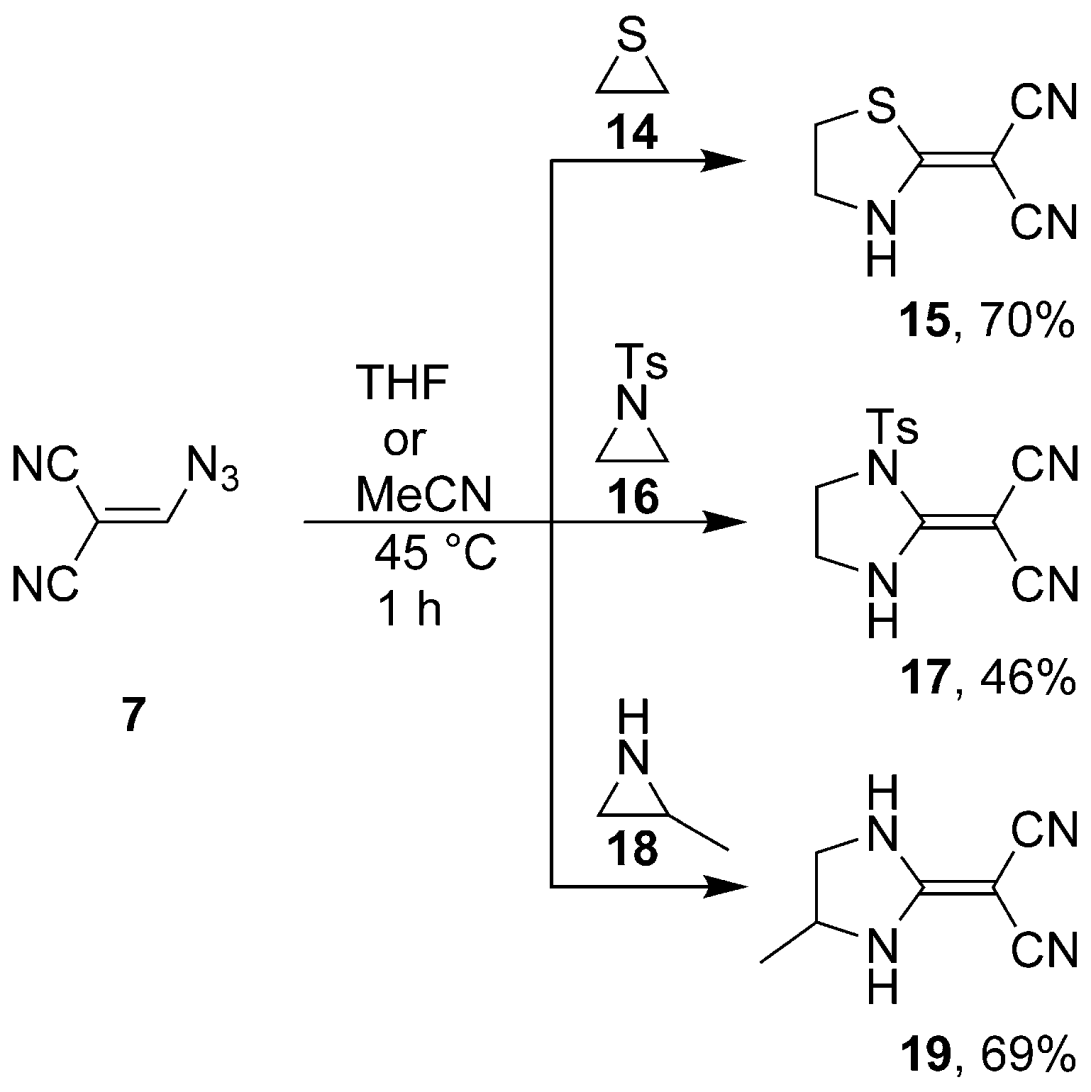
Ring enlargement of **14**, **16**, and **18**.

**Figure 2:**
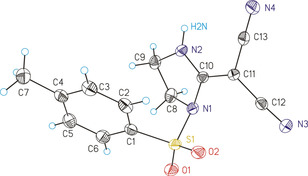
ORTEP (50 % ellipsoid probability) of the molecular structure of **17**. A second molecule in the asymmetric unit has been omitted for clarity. Selected bond properties ([Å]/[°]): S1−N1 1.6929(13), N1−C8 1.4979(18), C8−C9 1.525(2), C9−N2 1.463(2), N2−C10 1.3239(18), C10−N1 1.4121(18), C10−C11 1.388(2), C11−C12 1.418(2), C12−N3 1.147(2), C11−C13 1.421(2), C13−N4 1.1508(19); C8‐N1‐C10 105.08(11), C9‐N2‐C10 112.78(13), N1‐C10‐N2 110.05(13), C12‐C11‐C13 117.06(13), N1‐C8‐C9‐N2 26.89(14).

The ring‐enlargement reaction with the help of vinyl azide **7** was also transferred from oxiranes **9** to thiirane (**14**) and aziridines **16** and **18** to obtain the five‐membered heterocycles **15**,^21^ **17**, and **19**, respectively (Scheme 3). The ^13^C NMR spectrum of imidazole derivative **17**, measured in [D_6_]DMSO at room temperature, showed only a broad signal for both cyano groups, whereas the corresponding spectrum of a solution in [D_6_]acetone exhibited two signals at ambient temperature. When the temperature was raised to 45 °C in the latter case, coalescence of the two signals was observed. Rapid exchange of both cyano groups was obviously initiated by rotation about the push–pull‐substituted C=C bond. The π system of this bond is weakened owing to a dipolar resonance structure, which includes a positive charge, stabilized by the ring nitrogen atoms, and a negative charge delocalized by the cyano groups. The molecular structures of the heterocyclic products **15**, **17**, and **19** were confirmed by the usual spectroscopic characterization and also by single crystal X‐ray diffraction analysis of **17** (Figure 2).

**Scheme 4:**
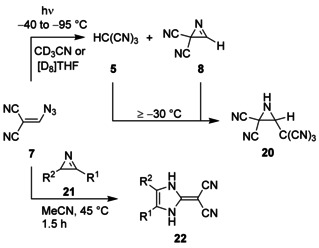
Reaction of different 2*H*‐azirines with cyanoform (**5**); for the key of R^1^ and R^2^, see Table 2.

Finally, we treated the 2*H*‐azirines **21** with the azide **7** to induce the reaction of in situ generated cyanoform (**5**) with these highly strained three‐membered heterocycles (Scheme 4). Since the addition of **5** at the C=N bond of azirine **8**, which was performed by irradiating of **7** at low temperatures and warming the resulting mixture of the photoproducts **5** and **8** to ≥−30 °C, was reported to lead to the unstable aziridine **20**,^8^ we expected analogous final products from substrates **7** and **21**. But the latter starting compounds afforded novel ring‐extended imidazole derivatives **22** instead of aziridines. The products **22 a**–**d** were formed with 56–62 % yield; and in the case of the very unstable^22^ 2*H*‐azirine **21 b**, which does not possess a substituent in the 3‐position, the yield was even slightly higher than that for the transformation of the robust^23^ substrate **21 a**, although the corresponding products **22 a** and **22 b** are identical (Table 2). It turned out that acetonitrile was a better solvent for this ring‐enlargement reaction than tetrahydrofuran since more convenient workup was possible and pure products were obtained easily. The new heterocycles **22** were characterized not only by the usual spectroscopic methods, but also by ^15^N NMR spectra of **22 a** and **22 c**. After assignment of the two NH proton signals of **22 a** by homonuclear NOE experiments, 2D‐^15^N,^1^H shift correlation (HSQC) also facilitated the allocation of the ^15^N NMR signals. In the ^13^C and ^15^N NMR spectra of the unsymmetrical heterocyclic compound **22 a**, both cyano groups led to a single signal because of a rapid rotation about the exocyclic C=C bond. Even at low temperature (−60 °C, 100 MHz, [D_7_]DMF), the ^13^C NMR signal of the two cyano groups was not split into two lines. Apparently, the dipolar resonance structure of **22 a**,^24^ in which the positive charge is delocalized in an aromatic imidazolium ring, is more pronounced than in the case of non‐aromatic heterocycle **19**. This assumption might be supported by the fact that ^13^C NMR chemical shifts of the exocyclic C=C moiety in **22 a** (*δ*=24.5 and 149.3, Δ*δ*=124.8) show a greater difference than those of **19** (Δ*δ*=109.6).

**Table 2:** Treatment of **21 a**–**d** with **7** to produce **22 a**–**d**.

**21/22**	R^1^	R^2^	Yield of **22** [%]^[a]^
**a**	Ph	H	58
**b**	H	Ph	61
**c**	Ph	Ph	56
**d**	Ph	Me	62

[a] Isolated yields.

**Scheme 5:**
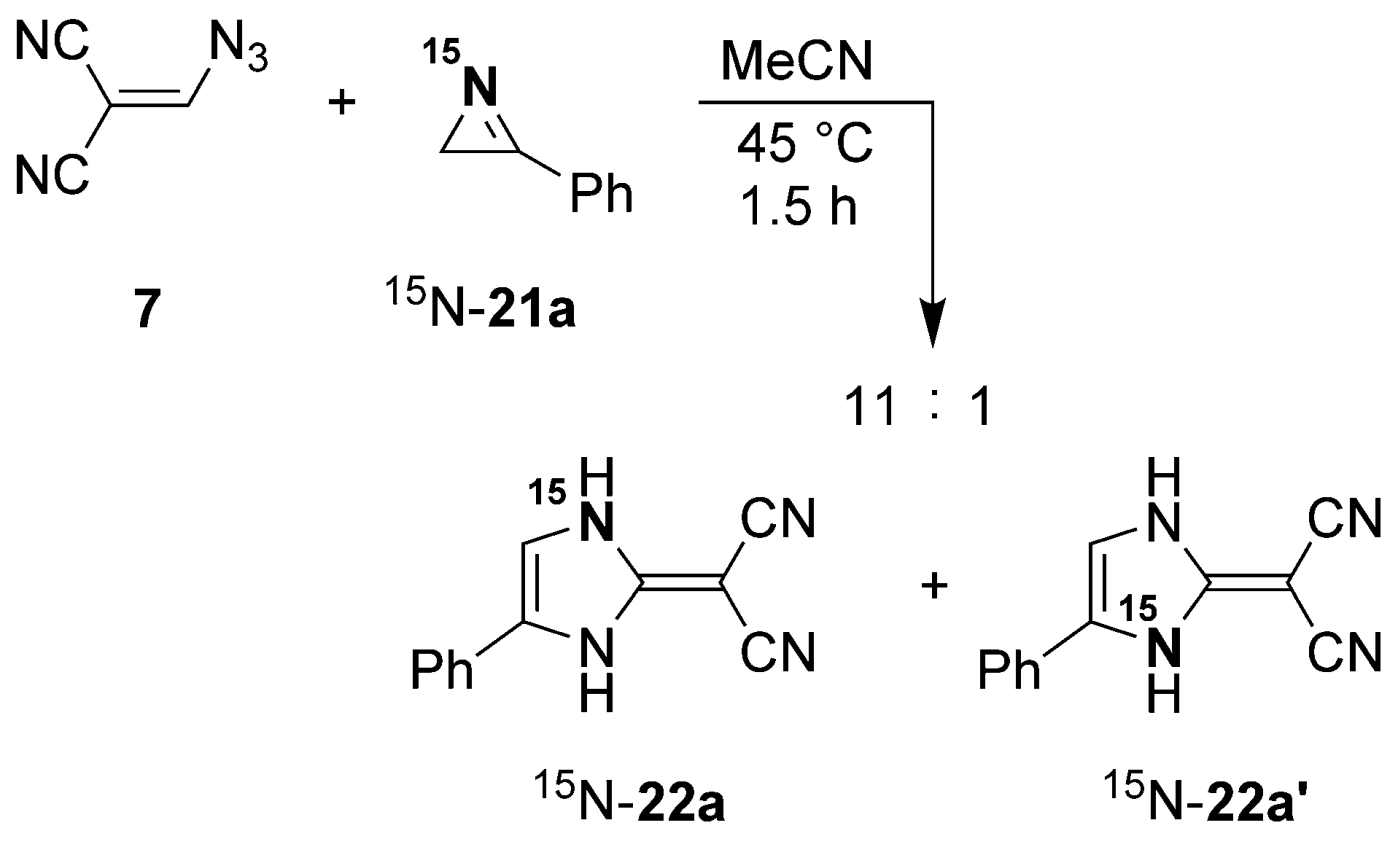
Ring enlargement of ^15^N‐**21 a**.

Whereas the formation of **20** is connected with an addition at the C=N bond of *N*‐protonated **8** and obviously induced by tricyanomethanide, acting as a carbon nucleophile, the genesis of **22** can only be explained by interaction of the protonated *2H*‐azirines **21** with tricyanomethanide, which reacts as a nitrogen nucleophile. Since at least two mechanisms may rationalize the **21**→**22** ring enlargement, we utilized the ^15^N‐labeled substrate ^15^N‐**21 a**,^25^ which was prepared by thermolysis of the corresponding α‐azidostyrene, and included 49 % of the nitrogen label (Scheme 5). After treatment of ^15^N‐**21 a** with **7**, we obtained, besides unlabeled **22 a**, the products ^15^N‐**22 a** and ^15^N‐**22 a′** in a ratio of 11:1. The quantitative analysis of ^15^N‐**22 a** was facilitated by the ^1^H NMR signal of ^15^NH‐1 at *δ*=12.50, which indicated ^1^ *J*(^15^N,^1^H)=97.6 Hz with ^1^H,^1^H triplet splitting (*J=*2.2 Hz), whereas ^15^N‐**22 a′** showed a ^1^H NMR signal of ^15^NH‐3 at *δ*=12.68 with ^1^ *J*(^15^N,^1^H)=96.0 Hz and additional ^1^H,^1^H triplet splitting (*J=*2.2 Hz). These results were supported by the ^15^N NMR spectrum, which included the ^15^NH‐1 signal of ^15^N‐**22 a** at *δ*=−233.6 with ^1^ *J*(^15^N,^1^H)=97.4 Hz and additional long‐range coupling (dd, *J=*4.6 and 3.7 Hz) as well as the ^15^NH‐3 signal of ^15^N‐**22 a′** at *δ*=−238.9 with ^1^ *J*(^15^N,^1^H)=96.4 Hz and triplet splitting with ^3^ *J*=3.4 Hz. The assignment of all ^1^H and ^15^N NMR signals were based not only on the previous allocation of the corresponding NMR signals of **22 a** (see above), but also on additional NOE experiments with ^15^N‐**22 a**/^15^N‐**22 a′**.

**Scheme 6:**
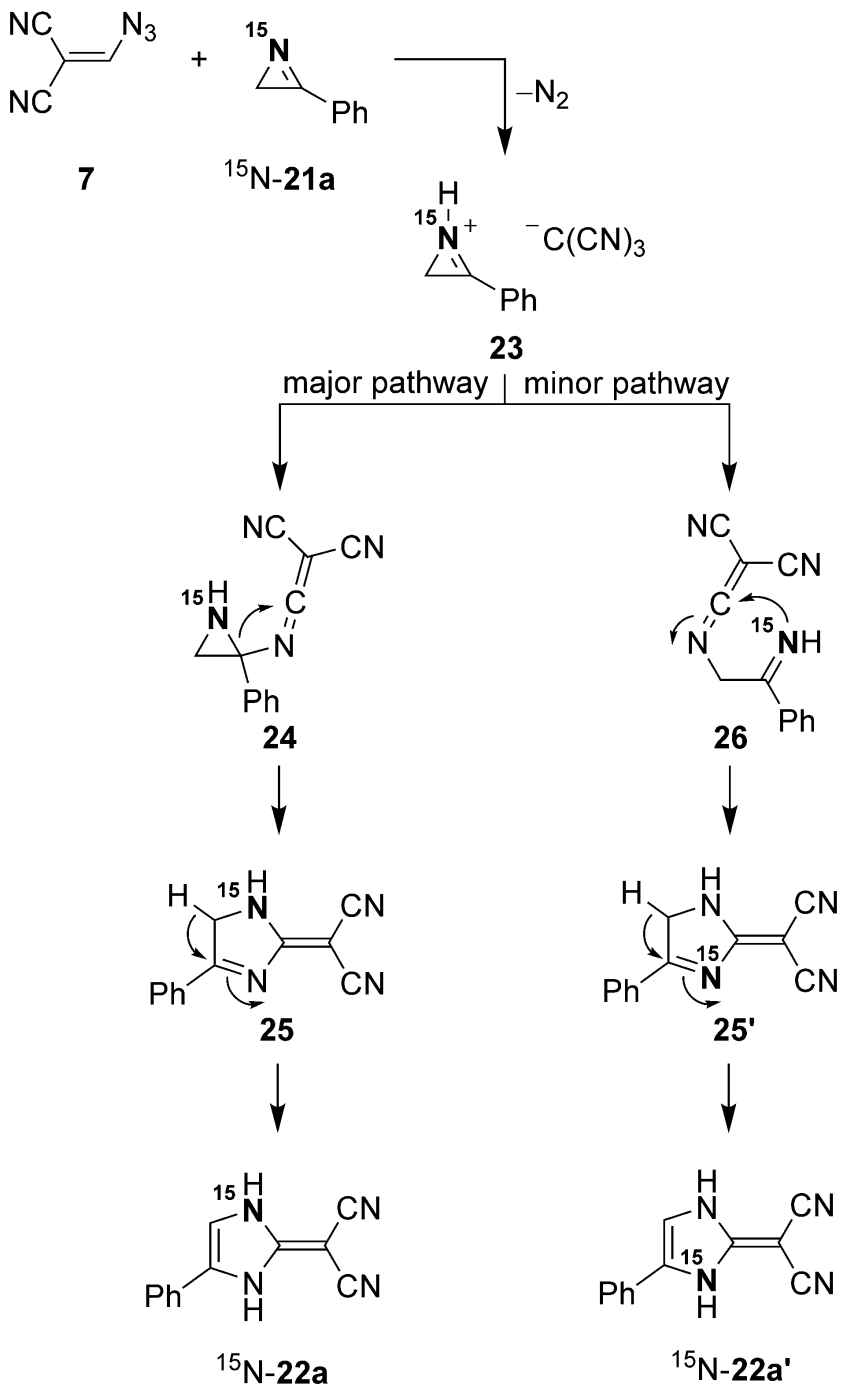
Reaction mechanisms which explain the formation of ^15^N‐**22 a** and ^15^N‐**22 a′** from **7** and ^15^N‐**21 a**.

We assume that formation of tricyanomethane (**5**) from **7** and subsequent *N*‐protonation of ^15^N‐**21 a** with generation of the ion pair **23** are always the first steps in the ring enlargement reaction of ^15^N‐**21 a** (Scheme 6). Thereafter, attack of the nitrogen nucleophile tricyanomethanide at the highly activated C=N unit of **23** leads to the addition product **24**, which undergoes a ring expansion by a 1,3‐migration process. The resulting imidazole derivative **25** tautomerizes to yield the main product ^15^N‐**22 a**. However, a minor pathway with nitrogen‐centered nucleophilic attack of tricyanomethanide at the sp^3^‐hybridized carbon atom achieves ring opening of **23** with formation of **26**; and successional cyclization of this ketenimine intermediate, followed by tautomerism of **25′**, generates ^15^N‐**22 a′**. The sequence ^15^N‐**21 a**→**23**→**26**→**25′** is similar to the mechanisms, which we suggest for the corresponding ring‐enlargement reactions of epoxides **9**, thiirane **14**, and aziridines **16** and **18**.

**Scheme 7:**
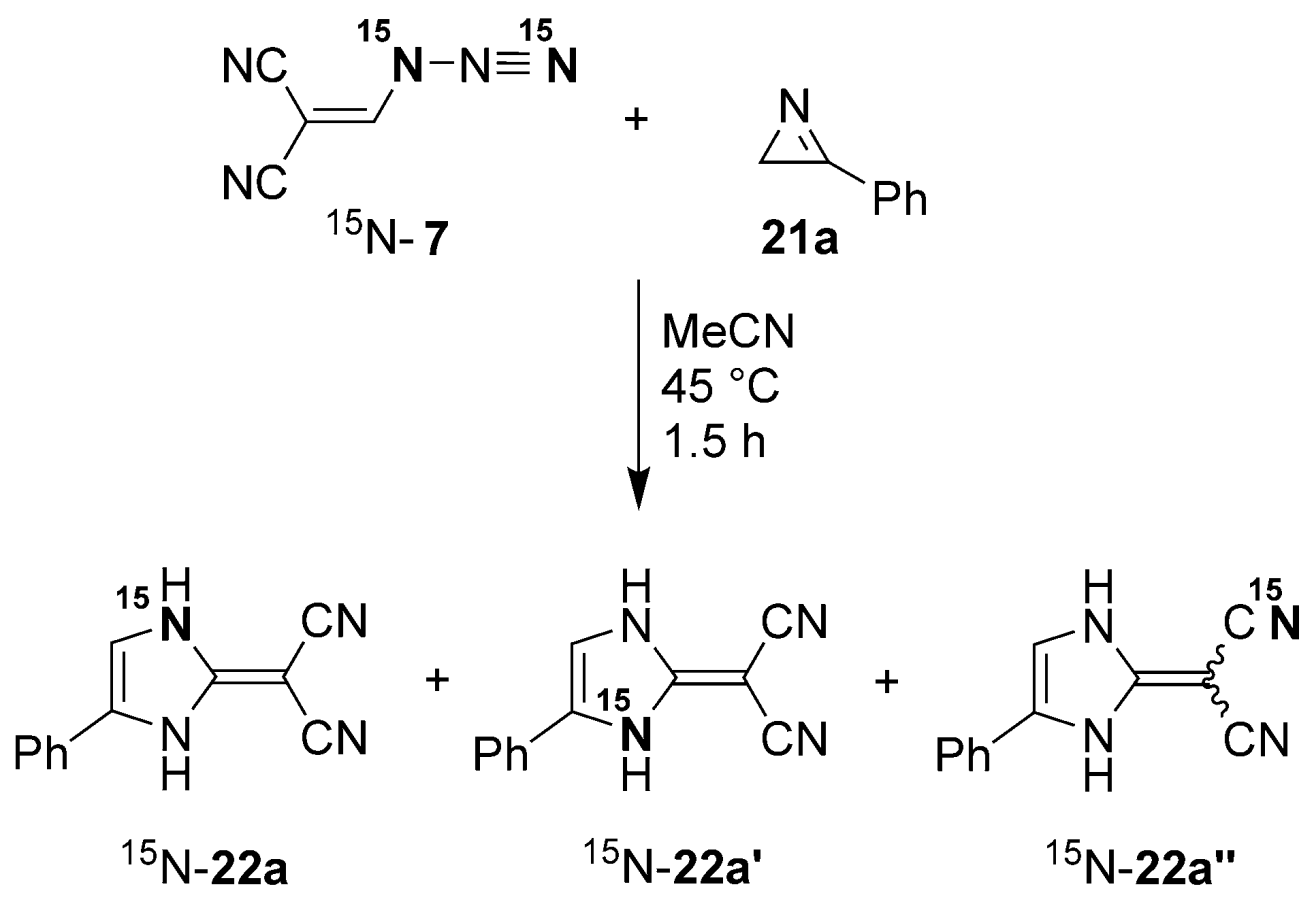
Ring enlargement of **21 a** with the help of ^15^N‐**7**.

In order to confirm the proposed reaction mechanisms of the transformation **7**+**21 a**→**22 a**, we utilized also the ^15^N‐labeled azide ^15^N‐**7**, which was prepared from (chloromethylidene)malonodinitrile and sodium azide that included 98 % of the isotope label at one of the terminal positions (Scheme 7). Since we used a smaller amount of ^15^N‐**7** and because of the label distribution on the three cyano positions of the resulting cyanoform, the experiment with ^15^N‐**7** was significantly less sensitive than that with ^15^N‐**21 a**. As expected, we detected just a small NH signal of ^15^N‐**22 a** besides strong signals of ^15^N‐**22′** and NH‐unlabeled **22 a** and ^15^N‐**22 a′′** in the ^1^H NMR spectrum, which was measured after treating ^15^N‐**7** with **21 a**. The corresponding ^15^N NMR spectrum indicated only ^15^NH‐3 (*δ*=−238.6, dt, ^1^ *J*=96.4 Hz, ^3^ *J*=3.4 Hz) of ^15^N‐**22 a′** and C≡^15^N (*δ*=−113.3, s) of ^15^N‐**22 a′′**. Thus, the transformation of ^15^N‐**7** proved to be a complementary confirmation of the experiment with ^15^N‐**21 a**.

**Scheme 8:**
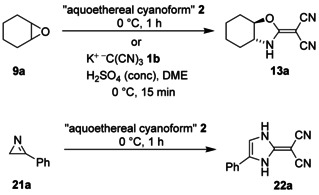
Ring enlargement of **9 a** and **21 a** with cyanoform generated from tricyanomethanide salts.

Ring‐expansion reactions of **9**, **14**, **16**, **18**, and **21** were performed with the aid of cyanoform precursor **7**, which was used under aprotic starting conditions and without competing nucleophiles. Hence, the question arose whether the corresponding ring‐enlargement products can also be prepared if aquoethereal cyanoform (**2**) or similar reagents are utilized. In order to find an answer to this question, we treated epoxide **9 a** with compound **2** and obtained the desired product **13 a** in 75 % yield (Scheme 8). However, **13 a** was accompanied by a small amount of *trans*‐cyclohexane‐1,2‐diol, which was simultaneously generated from **9 a** owing to the presence of water. Thus, the separation and purification of **13 a** was quite tedious if compared to the workup after the transformation **7**+**9 a**→**13 a** and similar reactions. Consequently, we tried anhydrous acidification of **1 b** and added concentrated sulfuric acid to a solution of **1 b** and **9 a** in 1,2‐dimethoxyethane (DME). After optimization of the reactions conditions, we isolated **13 a** by simple washing of the crude product with a limited amount of dichloromethane in 79 % yield. When we exposed the azirine **21 a** to the reagent **2**, we obtained the wanted product **22 a**, but the yield was disappointing low (10 %). By using the procedure with **1 b** and concentrated sulfuric acid in DME, we did not get the desired compound **22 a** from **21 a** at all. Hence, ring enlargement of three‐membered heterocycles with the help of the alternative cyanoform precursor **1 b** is possible, but there are limitations in some cases.

## Conclusions

In summary, we have demonstrated that tricyanomethane (**5**), in situ generated by thermal decay of vinyl azide **7** or by acidification of tricyanomethanide salt **1 b**, can successfully be used to perform ring‐expansion reactions with three‐membered heterocycles. The resulting products are formed via varying reaction mechanisms; however, tricyanomethanide always acts as a nitrogen nucleophile. This is quite different to the known simple alkylation of this nucleophilic species in the presence of alkyl halides, which leads to 1,1,1‐tricyanoalkanes by attack of a carbon nucleophile.^15^ It can be argued that different reaction conditions of ring enlargement and alkylation are the reason for distinct nucleophilic properties of tricyanomethanide. But we will show in the near future that tricyanomethane (**5**), also generated in situ by warming of **7** or by acidification of **1 b**, operates as a carbon nucleophile in Michael addition reactions.

Products of ring expansion, such as **13**, **15**, **17**, **19**, and **22**, include an exocyclic push–pull‐substituted C=C unit, and similar compounds were previously investigated intensively because of their structures, electronic properties, and rotational barriers.^24^, ^26^ Moreover, such substances were utilized for organic synthesis, in particular, as precursors of (other) heterocyclic skeletal structures,^27^ and in some cases, the biological properties were tested, for example, as plant growth regulators^28^ or for histamine H_3_ receptor‐binding affinities.^29^ Several methods are known to prepare these push–pull‐substituted olefinic compounds. In most cases, however, the access required a multi‐step synthesis.^19^, ^21^, ^30^ Thus, the simple cyanoform‐induced transformation of three‐membered heterocycles into ring‐enlargement products, such as **13**, **15**, **17**, **19**, and **22**, offers a new synthetic approach for the desired push–pull‐substituted alkenes.

## Experimental Section

Experimental details, ^1^H, ^13^C, ^15^N NMR spectra, crystal structure data and refinement details are given in the Supporting Information.

## Conflict of interest

The authors declare no conflict of interest.

## Supporting information

As a service to our authors and readers, this journal provides supporting information supplied by the authors. Such materials are peer reviewed and may be re‐organized for online delivery, but are not copy‐edited or typeset. Technical support issues arising from supporting information (other than missing files) should be addressed to the authors.

Supplementary
