# Supplementary material for: Ring Enlargement of Three‐Membered Heterocycles by Treatment with In Situ Formed Tricyanomethane
Source: Chemistry. 2020 Apr 30;26(28):6158–64. doi: 10.1002/chem.202000089 (PMC7318174; doi:10.1002/chem.202000089)
Supplement: Supplementary file 1 — Supplementary [file CHEM-26-6158-s001.pdf]

Chemistry–A European Journal

Supporting Information

Ring Enlargement of Three-Membered Heterocycles by Treatment with In Situ Formed Tricyanomethane**

Klaus Banert,*[a] Madhu Chityala,[a] and Marcus Korb[b]

Supporting Information

Ring Enlargement of Three-Membered Heterocycles by Treatment with In Situ Formed Tricyanomethane**

Klaus Banert,*[a] Madhu Chityala,[a] and Marcus Korb[b]

[a]Organic Chemistry, Chemnitz University of Technology, Strasse der Nationen 62, 09111 Chemnitz (Germany)

[b]The University of Western Australia, Faculty of Science, School of Molecular Scienes, 35 Stirling Highway, Crawley, Perth, Western Australia 6009 (Australia)

Table of Contents

Experimental Procedures

General methods……………………………………………………………S-2

1. Synthesis of azide **7**…………………………………………………………...S-2
2. Reaction of azide **7** with various epoxides and thiirane……………………..S-3
3. Reaction of azide **7** with various aziridines and azirines……………………..S-9
4. Synthesis and reactions of aquoethereal cyanoform **2** solution……..…..….S-17
5. Direct reaction of potassium tricyanomethanide salt (**1b**) with cyclohexene oxide (**9a**) in presence of conc. H_2SO_4…………….…………………………..…S-18
6. NMR spectra…………………………………………………………………...S-19
7. References……………………………………………………………………..S-74

Experimental Procedures

General Methods: Melting points were determined with a Pentakon Dresden Boetius apparatus. FTIR spectra were recorded on Nicolet iS5 spectrophotometer from Thermo Fisher Scientific. Solutions in KBr cuvetts or KBr pellets were used for the IR measurements. ^{1}H NMR spectra were recorded with Unity Inova 400 spectrometer operating at 400 MHz. By using the same spectrometer, ^{13}C NMR (100 MHz) and ^{15}N NMR (40.5 MHz) were recorded. Chemical shifts δ are given in ppm and were referenced to the solvent signals ($CDCl_3$: 7.24 ppm (^{1}H), 77.0 ppm (^{13}C), CD_3CN: 1.94 ppm (^{1}H), 1.32 ppm (^{13}C), DMSO-d_6: 2.50 ppm (^{1}H), 39.52 ppm (^{13}C), acetone-d_6: 2.05 ppm (^{1}H), 29.84 ppm (^{13}C)). The multiplicities of ^{13}C NMR signals were determined with the aid of DEPT-135 experiments. The multiplicities were described using the following abbreviations and their combinations: s (singlet), d (doublet), t (triplet), q (quartet), m (multiplet). ^{1}H NMR yields were measured with solvent as internal standard. Single crystal X-ray diffraction analysis was performed by Dr. M. Korb on an Oxford Gemini S diffractometer. HRMS (ESI) spectra were recorded with a Bruker micrOTOF-QII spectrometer. TLC was carried out with Macherey-Nagel Polygram SIL G/UV254 polyester sheets.

Single crystal X-ray diffraction analysis. Data were collected with an Oxford Gemini S diffractometer with Cu K_α radiation (λ = 1.54184 Å; **13g**) and Mo K_α radiation (λ = 0.71073 Å, **17**) at 110 K. The molecular structures were solved by direct methods using SHELXS-13[S-1] and refined by full-matrix least-squares procedures on F^2 using SHELXL-13.[S-2,S-3] All non-hydrogen atoms were refined anisotropically and a riding model was employed in the treatment of the C-bonded hydrogen atom positions. The N-bonded H atoms in both structures were placed according to residual electron density and refined isotropically. In case of **13g** the N–H distance was restrained using DFIX 0.900(0.02). Compound **17** was refined using the TWIN instruction (BASF = 0.05783). Graphics of the molecular structures have been created by using ORTEP.[S-4]

Crystallographic data have been deposited at the Cambridge Crystallographic Database and is accessible under CCDC numbers: 1974589 (**13g**) and 1974590 (**17**) via www.ccdc.cam.ac.uk/data_request/cif.

*Crystal Data for **13g***: $C_{18}H_{13}N_3O$, M = 287.31 g mol^{-1}, colorless needle, 0.4·0.02·0.02, orthorhombic, *Pna21*, λ = 1.54184 Å, a = 22.424(4) Å, b = 4.6422(10) Å, c = 28.741(4)

Å, V = 2991.8(10) Å^3, Z = 8, ρ_{calcd} = 1.276 Mg m^{-3}, μ = 0.655 mm^{-1}, F_{000} = 1200, T = 110 K, θ range 3.075−62.444°, 9287 reflections collected, 4376 independent reflections (R_{int} =0.2663), *GooF* = 0.759, *R*1 = 0.1233, *wR*2 = 0.2014 ($I>2\sigma(I)$), $|\Delta\rho|_{max}$ = 0.277, 351 parameters, 443 restraints, absolute structure parameter[S-5]: –0.2(10).

Crystal Data for ***17***: $C_{13}H_{12}N_4O_2S$, M = 288.33 g mol^{-1}, colorless block, 0.4·0.2·0.2, monoclinic, $P2_1/c$, λ = 0.71073 Å, a = 21.8963(10) Å, b = 6.8042(2) Å, c = 20.5201(9) Å, β = 117.635(6) °, V = 2708.5(2) Å^3, Z = 8, ρ_{calcd} = 1.414 Mg m^{-3}, μ = 0.246 mm^{-1}, F_{000} = 1200, T = 110.05(10) K, θ range 2.994−26.000°, 33884 reflections collected, 5286 independent reflections (R_{int} =0.0241), *GooF* = 1.025, *R*1 = 0.0295, *wR*2 = 0.0738 ($I>2\sigma(I)$), $|\Delta\rho|_{max}$ = 0.326, 372 parameters, 0 restraints.

1. Synthesis of azide 7: Azide **7** was synthesized by following an analogous procedure from literature.[S-6,S-7]

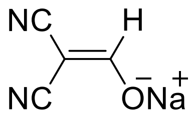

Sodium salt of hydroxymethylidene-malonodinitrile:[S-6] A solution of sodium ethoxide was prepared by dissolving sodium metal (6.90 g, 0.30 g-atoms) in anhydrous ethanol (225 mL). To this warm solution, malononitrile (19.80 g, 0.30 mol), ethyl formate (45.00 g, 49.07 mL, 0.60 mol), and anhydrous ethanol (45 mL) were added successively. The resulting reaction mixture was stirred under reflux conditions for 1.5 h, cooled, and the precipitated sodium salt of hydroxymethylidene-malonodinitrile was collected by filtration. The solid on the filter was washed thoroughly with ether and dried under vacuum. The yield of the precursor was 65% (22.40 g). ^{1}H NMR (400 MHz, DMSO-d$_6$): δ = 8.62 (s, CH). ^{13}C NMR (100.6 MHz, DMSO-d$_6$): δ = 49.30 (s, *C*–CN), 119.13 (s, CN), 122.16 (s, CN), 181.91 (d, CH).

Note: Major quantity of the product was precipitated out upon addition of diethyl ether.

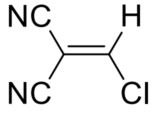

1-Chloro-2,2-dicyanoethylene:[S-6] A slurry of dry sodium salt of hydroxymethylidene-malonodinitrile (11.60 g, 0.10 mol) in anhydrous CH_2Cl_2 (78.6 mL) was treated with phosphorous oxychloride ($POCl_3$) (15.30 g, 9.3 mL, 0.10 mol) in one portion. The resulting reaction mixture was stirred under reflux for 4 h, cooled, and filtered. The solid on the filter was washed with fresh CH_2Cl_2, and the combined filtrate was concentrated by rotavap at reduced pressure.

The resulting residue was distilled to provide 32% (3.60 g) of 1-chloro-2,2-dicyanoethylene, b.p. 73 °C (10 mm). ^{1}H NMR (400 MHz, $CDCl_3$): δ = 7.76 (s, CH). ^{13}C NMR (100.6 MHz, $CDCl_3$): δ = 93.43 (s, *C*–CN), 108.71 (s, CN), 109.91 (s, CN), 154.41 (d, CH).

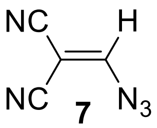

7

2-(Azidomethylidene)malonodinitrile (7):[S-7] A suspension of sodium azide (0.77 g, 11.84 mmol) in anhydrous CH_3CN (10.0 mL) was left to stir at 0 °C. To this suspension, a solution of 1-chloro-2,2-dicyanoethylene (1.12 g, 9.94 mmol) in anhydrous CH_3CN (4.0 mL) was added dropwise. The reaction mixture was stirred at 0 °C for 1.5 h and then filtered. The solid on the filter was extracted with diethyl ether, and the combined filtrate was concentrated in vacuum at room temperature with a protecting shield in front of the rotavap. The isolated yield of **7** was 99.5% (1.18 g). ^{1}H NMR (400 MHz, CD_3CN): δ = 8.22 (s, CH). ^{13}C NMR (100.6 MHz, CD_3CN): δ = 71.81 (s, *C*–CN), 110.97 (s, CN), 113.22 (s, CN), 163.41 (d, CH). UV (in CH_3CN): The molar extinction coefficient of **7**, $\varepsilon \approx 18.6518\text{x}10^3$ $M^{-1}cm^{-1}$ (λ = 281.6 nm, *A* (absorbance) = 2.02, d = 1 cm, c = $0.1083\text{x}10^{-3}$ M).

2. Reaction of azide 7 with various epoxides and thiirane:

General procedure-I: To a solution of azide **7** (0.84 mmol to 4.19 mmol) in anhydrous THF (7 mL), was added a solution of the corresponding epoxide (0.84 mmol to 8.39 mmol) in anhydrous THF (3 mL). The resulting reaction mixture was stirred at 45 °C for 1 h. Progress of the reaction was monitored by TLC (*n*-hexane:ethyl acetate = 1:1). After completion of the reaction, the solvent was removed under vacuum and the resulting crude product was purified by column chromatography eluting with *n*-hexane/EtOAc. In some cases, where the product is insoluble or very less soluble in CH_2Cl_2, to the crude residue obtained after rotavap was added minimum amount of CH_2Cl_2, filtered, and washed the solid with minimum amount of CH_2Cl_2 and dried under vacuum (using excess amount of CH_2Cl_2 will lower the yield of the product if it is partially soluble in CH_2Cl_2).

Note-1: Reaction of one equivalent of azide **7** with two equivalents of an epoxide or vice versa did not affect the yield of the product much. In most of the cases, excess amount of azide **7** was used.

Note-2: Always avoid addition of pure liquid strained compound to the solid azide **7**. Because, the azide **7** is highly reactive towards strained compounds and will lead to explosion. The best way is the addition of solution of strained component to the solution of azide **7** or vice versa at RT or at 0 ^{o}C (if necessary, depending on the reactivity of strained component).

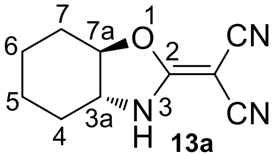

13a
56%

Reaction of azide 7 with cyclohexene oxide (**9a**)**:** Following the general procedure-I, the azide **7** (500.0 mg, 4.19 mmol) was treated with cyclohexene oxide **9a** (824.0 mg, 8.39 mmol). The product was purified by using CH_2Cl_2 as described in general procedure-I. Light yellow coloured solid **13a** (445.0 mg, 56%), m.p. 246–247 ^{o}C (recrystallized from MeOH). IR (KBr): ṽ = 3216 (br), 2943 (m), 2203 (s), 1588 (s) cm^{-1}. ^{1}H NMR (400 MHz, DMSO-d$_6$): δ = 1.25 (qt, ^{3}J = 4.0 Hz, ^{2}J = 12.8 Hz, 1H, 5-H$_a$), 1.33 (qt, ^{3}J = 3.8 Hz, ^{2}J = 12.6 Hz, 1H, 6-H$_a$), 1.43 (qd, ^{3}J = 4.0 Hz, ^{2}J = 12.0 Hz, 1H, 4-H$_a$), 1.69 (qd, ^{3}J = 4.0 Hz, ^{2}J = 12.0 Hz, 1H, 7-H$_a$), 1.72 (br d, ^{2}J ≈ 11.6 Hz, 1H, 5-H$_e$), 1.83 (qd, ^{3}J ≈ 4.0 Hz, ^{2}J = 12.4 Hz, 1H, 6-H$_e$), 2.07 (qd, ^{3}J = 4.0 Hz, ^{2}J = 12.0 Hz, 1H, 4-H$_e$), 2.22 (qd, ^{3}J = 4.0 Hz, ^{2}J = 12.0 Hz, 1H, 7-H$_e$), 3.43 (td, ^{3}J = 4.0 Hz, ^{3}J = 12.0 Hz, 1H, 3a-H$_a$), 4.13 (td, ^{3}J = 4.0 Hz, ^{3}J = 12.0 Hz, 1H, 7a-H$_a$), 10.25 (s, 1H, NH). ^{13}C NMR (100.6 MHz, DMSO-d$_6$): δ = 22.75 (t, C-6), 23.06 (t, C-5), 27.60 (t, C-7), 27.83 (t, C-4), 33.55 (s, *C*-CN), 61.54 (d, C-3a), 88.56 (d, C-7a), 115.05 (s, CN), 115.39 (s, CN), 173.67 (s, C-2). HRMS (ESI): *m/z* calcd. for $C_{10}H_{12}N_3O$ ([M+H]$^+$): 190.0980, found: 190.0975; calcd. for $C_{10}H_{11}N_3ONa$ ([M+Na]$^+$): 212.0800, found: 212.0794.

Assignment of the NMR signals was done by using gCOSY, gHSQCAD, and gHMBCAD

Note: H$_a$-axial proton, H$_e$-equatorial proton.

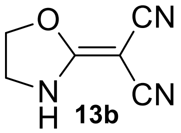

13b
72%

Reaction of azide 7 with ethylene oxide (**9b**)**:** Following the general procedure-I, the azide **7** (200.0 mg, 1.68 mmol) was treated with ethylene oxide **(9b)** gas (380.9 mg, 8.64 mmol) dissolved in THF at –15 ^{o}C, and the reaction mixture was stirred at RT. The product was purified by column chromatography eluting with *n*-hexane/EtOAc. Light yellow colored solid **13b** (163.3 mg, 72%), m.p. 163–164 ^{o}C (recrystallized from CD_3CN). The product **13b** was known in literature.[S-8] IR (KBr): ṽ = 2217 (s), 1632 (s) cm^{-1}. ^{1}H

NMR (400 MHz, CD_3CN): δ = 3.77 (t, J = 8.6 Hz, 2H, CH_2–N), 4.70 (t, J = 8.6 Hz, 2H, CH_2–O), 7.55 (s, 1H, NH). ^{13}C NMR (100.6 MHz, CD_3CN): δ = 33.62 (s, *C*–CN), 44.67 (t, CH_2–N), 72.75 (t, CH_2–O), 115.78 (s, CN), 116.29 (s, CN), 174.70 (s, *C*=C(CN)$_2$). HRMS (ESI): *m/z* calcd. for $C_6H_6N_3O$ ([M+H]$^+$): 136.0511, found: 136.0505; calcd. for $C_6H_5N_3ONa$ ([M+Na]$^+$): 158.0330, found: 158.0325.

Reaction of azide 7 with 2-methyloxirane (9c): Azide **7** (200.0 mg, 1.68 mmol) was treated with 2-methyloxirane **(9c)** (195.1 mg, 3.36 mmol) following the general procedure-I and it produced two regioisomeric products. The two regioisomers were separated by column chromatography eluting with *n*-hexane/EtOAc.

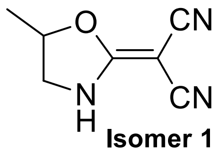

Isomer 1
25%

Isomer 1: White colored solid (62.6 mg, 25%), m.p. 150–151 ^oC (recrystallized from EtOAc/*n*-hexane). IR (KBr): ṽ = 3270 (br), 2219 (s), 1609 (s) cm^{-1}. ^1H NMR (400 MHz, $CDCl_3$): δ = 1.53 (d, J = 6.4 Hz, 3H, CH_3), 3.39 (t, J = 8.6 Hz, 1H, CH_2), 3.91 (t, J = 9.0 Hz, 1H, CH_2), 5.09 (tq, J = 6.4 Hz, J = 8.2 Hz, 1H, CH), 8.02 (s, 1H, NH). ^{13}C NMR (100.6 MHz, $CDCl_3$): δ = 19.74 (q, CH_3), 33.90 (s, *C*–CN), 49.94 (t, CH_2), 81.18 (d, CH), 114.07 (s, CN), 116.11 (s, CN), 173.35 (s, *C*=C(CN)$_2$). HRMS (ESI): *m/z* calcd. for $C_7H_7N_3O$ [M]$^+$: 149.0589, found: 149.0584; calcd. for $C_7H_7N_3ONa$ ([M+Na]$^+$): 172.0487, found: 172.0481; calcd. for $C_7H_7N_3OK$ ([M+K]$^+$): 188.0226, found: 188.0221.

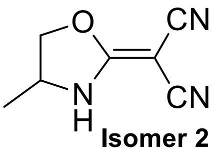

Isomer 2
22%

Isomer 2: White colored solid (55.1 mg, 22%), m.p. 130–131 ^oC (recrystallized from EtOAc/*n*-hexane). IR (KBr): ṽ = 3235 (br), 2220 (s), 1606 (s) cm^{-1}. ^1H NMR (400 MHz, $CDCl_3$): δ = 1.35 (d, J = 6.0 Hz, 3H, CH_3), 4.21 (t, J = 7.4 Hz, 1H, CH_2), 4.26 (sext, J = 6.6 Hz, 1H, CH), 4.76 (t, J = 7.8 Hz, 1H, CH_2), 8.35 (s, 1H, NH). ^{13}C NMR (100.6 MHz, $CDCl_3$): δ = 19.57 (q, CH_3), 33.65 (s, *C*–CN), 52.50 (d, CH), 77.09 (t, CH_2), 113.97 (s, CN), 115.91 (s, CN), 173.16 (s, *C*=C(CN)$_2$). HRMS (ESI): *m/z* calcd. for $C_7H_7N_3O$ [M]$^+$: 149.0589, found: 149.0584; calcd. for $C_7H_8N_3O$ ([M+H]$^+$): 150.0667, found: 150.0662; calcd. for $C_7H_7N_3ONa$ ([M+Na]$^+$): 172.0487, found: 172.0481.

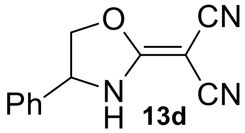

13d
58%

Reaction of azide 7 with 2-phenyloxirane (9d): Azide **7** (100.0 mg, 0.84 mmol) was reacted with 2-phenyloxirane **(9d)** (100.9 mg, 0.84 mmol) following the general procedure-I. The crude

product **13d** was purified by using CH_2Cl_2 as described in general procedure-I. Light yellow colored solid (103.0 mg, 58%), m.p. 180–181 °C. IR (KBr): ṽ = 3175 (br), 2226 (s), 1612 (s) cm^{-1}. ^{1}H NMR (400 MHz, DMSO-d$_6$): δ = 4.54 (dd, ^2J = 8.8 Hz, ^3J = 2.4 Hz, 1H, CH_2), 5.07 (dd, ^2J = ^3J = 9.0 Hz, 1H, CH_2), 5.30 (dd, ^3J = 9.0 Hz, ^3J = 2.4 Hz, 1H, CH), 7.35–7.46 (m, 5H, Ar-H), 10.83 (s, 1H, NH). ^{13}C NMR (100.6 MHz, DMSO-d$_6$): δ = 31.64 (s, *C*–CN), 59.45 (d, CH), 77.64 (t, CH_2), 115.43 (s, CN), 115.56 (s, CN), 126.68 (d, Ar-CH), 128.67 (d, Ar-CH$_{para}$), 128.95 (d, Ar-CH), 138.44 (s, Ar-C$_{ipso}$), 172.28 (s, *C*=C(CN)$_2$). Elemental analysis: $C_{12}H_9N_3O$ (211.22): calcd. C 68.24, H 4.29, N 19.89; found: C 68.02, H 4.40, N 19.36%.

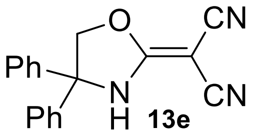

13e
79%

Reaction of azide 7 with 2,2-diphenyloxirane (13e): Azide **7** (100.0 mg, 0.84 mmol) was treated with 2,2-diphenyloxirane **(9e)**[S-9] (329.8 mg, 1.68 mmol) following the general procedure-I. The crude product **13e** was purified by column chromatography eluting with *n*-hexane/EtOAc. Light yellow colored solid (190.6 mg, 79%), m.p 184–185 °C (recrystallized from $CDCl_3$). IR (KBr): ṽ = 3225 (br), 2221 (m), 1612 (s) cm^{-1}. ^{1}H NMR (400 MHz, $CDCl_3$): δ = 5.12 (s, 2H, CH_2), 7.25 (dq, J = 8.7 Hz, J = 2.1 Hz, 4H, Ar-H), 7.39–7.45 (m, 6H, Ar-H), 9.42 (s, 1H, NH). ^{13}C NMR (100.6 MHz, $CDCl_3$): δ = 34.79 (s, *C*–CN), 71.88 (s, *C*–Ph), 81.56 (t, CH_2), 113.74 (s, CN), 115.11 (s, CN), 125.99 (d, Ar-CH), 128.73 (d, Ar-CH$_{para}$), 129.13 (d, Ar-CH), 140.28 (s, Ar-C$_{ipso}$), 172.70 (s, *C*=C(CN)$_2$). HRMS (ESI): *m/z* calcd. for $C_{18}H_{14}N_3O$ ([M+H]$^+$): 288.1137, found: 288.1131; calcd. for $C_{18}H_{13}N_3ONa$ ([M+Na]$^+$): 310.0956, found: 310.0951.

Reaction of azide 7 with *trans*-1,3-diphenyl-2,3-epoxypropan-1-one (9f): Following the general procedure-I, the azide **7** (200.0 mg, 1.68 mmol) was treated with *trans*-1,3-diphenyl-2,3-epoxypropan-1-one **(9f)** (188.3 mg, 0.84 mmol) and it produced two stereo isomers of **13f**. The two stereo isomers were separated by column chromatography eluting with *n*-hexane/EtOAc.

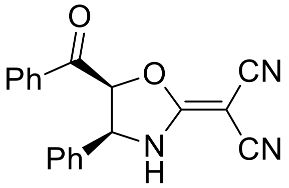

***cis*-Isomer**
27%

***cis*-Isomer:** Light yellow colored solid (71.5 mg, 27%), m.p. 217–218 °C (recrystallized from CD_3CN). IR (KBr): ṽ = 3172 (br), 2223 (s), 1693 (m), 1623 (s) cm^{-1}. ^{1}H NMR (400 MHz, CD_3CN): δ = 5.74 (d, J = 9.6 Hz, 1H, CH–N), 6.70 (d, J = 9.6 Hz, 1H, CH–O), 7.00 (dd, J = 8.0 Hz, J = 1.6 Hz, 2H, Ar-H),

7.07–7.13 (m, 3H, Ar-H), 7.34 (t, J = 8.0 Hz, 2H, Ar-H), 7.51–7.56 (m, 3H, Ar-H), 8.25 (s, 1H, NH). ^{13}C NMR (100.6 MHz, CD_3CN): δ = 35.01 (s, *C*–CN), 63.37 (d, CH–N), 86.99 (d, CH–O), 115.30 (s, CN), 115.56 (s, CN), 128.74 (d, Ar-CH), 129.05 (d, Ar-CH), 129.29 (d, Ar-CH), 129.59 (d, Ar-CH), 130.12 (d, Ar-CH_{para}), 134.24 (s, Ar-C_{ipso}), 135.11 (d, Ar-CH_{para}), 135.28 (s, Ar-C_{ipso}), 174.16 (s, *C*=C(CN)$_2$), 192.00 (s, C=O). HRMS (ESI): *m/z* calcd. for $C_{19}H_{14}N_3O_2$ ([M+H]$^+$): 316.1086, found: 316.1081; calcd. for $C_{19}H_{13}N_3O_2Na$ ([M+Na]$^+$): 338.0905, found: 338.0900.

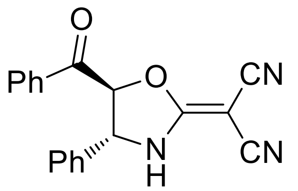
***trans*-Isomer**
26%

***trans*-Isomer:** Light yellow colored solid (68.6 mg, 26%), m.p. 169–170 °C (recrystallized from $CDCl_3$). IR (KBr): ṽ = 3173 (br), 2221 (s), 1696 (m), 1625 (s) cm^{-1}. ^{1}H NMR (400 MHz, $CDCl_3$): δ = 5.47 (d, J = 5.6 Hz, 1H, CH–N), 5.81 (d, J = 5.6 Hz, 1H, CH–O), 7.30 (dd, J = 7.6 Hz, J = 2.0 Hz, 2H, Ar-H), 7.43–7.52 (m, 5H, Ar-H), 7.65 (tt, J = 7.5 Hz, J = 1.1 Hz, 1H, Ar-H), 7.90 (dd, J = 8.4 Hz, J = 1.2 Hz, 2H, Ar-H), 8.24 (s, 1H, NH). ^{13}C NMR (100.6 MHz, $CDCl_3$): δ = 35.35 (s, *C*–CN), 61.60 (d, CH–N), 88.62 (d, CH–O), 112.96 (s, CN), 114.88 (s, CN), 126.29 (d, Ar-CH), 129.24 (d, Ar-CH), 129.29 (d, Ar-CH), 129.77 (d, Ar-CH), 129.95 (d, Ar-CH_{para}), 132.80 (s, Ar-C_{ipso}), 135.20 (d, Ar-CH_{para}), 136.11 (s, Ar-C_{ipso}), 172.38 (s, *C*=C(CN)$_2$), 189.74 (s, C=O). HRMS (ESI): *m/z* calcd. for $C_{19}H_{14}N_3O_2$ ([M+H]$^+$): 316.1086, found: 316.1081; calcd. for $C_{19}H_{13}N_3O_2Na$ ([M+Na]$^+$): 338.0905, found: 338.0900.

Reaction of azide 7 with *cis*-2,3-diphenyloxirane (9g):

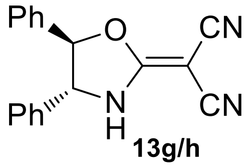
13g/h

Following the general procedure-I, the azide **7** (200.0 mg, 1.68 mmol) was treated with *cis*-2,3-diphenyloxirane **(9g)** (405.6 mg, 2.06 mmol). The product **13g/h** was separated from the by-products 1,2-diphenylethanone and 2,2-diphenylacetaldehyde (isolated together, 35.1 mg, 13%) by column chromatography eluting with *n*-hexane/EtOAc. Light yellow colored solid (182.2 mg, 46%), m.p. 173–174 °C (recrystallized from $CDCl_3$). IR (KBr): ṽ = 3159 (br), 2208 (m), 1630 (s) cm^{-1}. ^{1}H NMR (400 MHz, $CDCl_3$): δ = 5.08 (d, ^3J = 8.0 Hz, 1H, CH–N), 5.59 (d, ^3J = 8.0 Hz, 1H, CH–O), 7.27–7.33 (m, 4H, Ar-H), 7.46–7.48 (m, 6H, Ar-H), 8.79 (s, 1H, NH). ^{13}C NMR (100.6 MHz, $CDCl_3$): δ = 34.60 (s, *C*–CN), 67.62 (d, CH–N), 92.35 (d, CH–O), 113.54 (s, CN), 115.37 (s, CN), 126.15 (d, Ar-CH), 126.22 (d, Ar-CH), 129.24 (d, Ar-CH), 129.51 (d, Ar-CH), 129.65 (d, Ar-CH_{para}), 130.06 (d, Ar-CH_{para}), 134.53 (s, Ar-C_{ipso}), 135.50 (s, Ar-C_{ipso}), 172.98

(s, *C*=C(CN)2). HRMS (ESI): *m/z* calcd. for $C_{18}H_{14}N_3O$ ([M+H]$^+$): 288.1137, found: 288.1131.

Reaction of azide 7 with *trans*-2,3-diphenyloxirane (9h): Following the general procedure-I, azide **7** (164.0 mg, 1.37 mmol) was treated with *trans*-2,3-diphenyl-oxirane **(9h)** (405.6 mg, 2.06 mmol). The same *trans* product **13g/h** and the side products 1,2-diphenylethanone and 2,2-diphenylacetaldehyde (isolated together, 37.8 mg, 14%) were obtained as shown above, and were separated by column chromatography eluting with *n*-hexane/EtOAc. Light yellow colored solid (186.2 mg, 47%).

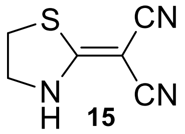

15
70%

Reaction of azide 7 with ethylene sulfide (14): Azide **7** (200.0 mg, 1.68 mmol) was treated with ethylene sulfide **(14)** (302.9 mg, 5.03 mmol) following the general procedure-I. The crude product **15** was purified by column chromatography eluting with *n*-hexane/EtOAc. Light yellow colored solid (177.7 mg, 70%), m.p. 218–219 ^oC (recrystallized from EtOAc). The product **15** is reported in literature.[S-10] IR (KBr): $\tilde{\nu}$ = 3197 (br), 2212 (s), 1575 (s) cm^{-1}. ^1H NMR (400 MHz, CD_3CN): δ = 3.47 (t, *J* = 7.4 Hz, 2H, CH_2), 3.92 (t, *J* = 7.6 Hz, 2H, CH_2), 7.45 (s, 1H, NH). ^{13}C NMR (100.6 MHz, CD_3CN): δ = 32.40 (t, CH_2) 44.89 (s, *C*–CN), 51.85 (t, CH_2), 115.44 (s, CN), 117.12 (s, CN), 178.90 (s, *C*=C(CN)2). HRMS (ESI): *m/z* calcd. for $C_6H_6N_3S$ ([M+H]$^+$): 152.0282, found: 152.0277; calcd. for $C_6H_5N_3SNa$ ([M+Na]$^+$): 174.0102, found: 174.0096.

3. Reaction of azide 7 with various aziridines and 2*H*-azirines:

General Procedure-II: To a solution of azide **7** (1.68 mmol to 2.28 mmol) in anhydrous CH_3CN (7 mL) was added a solution of the corresponding aziridine/2*H*-azirine (0.84 mmol to 3.36 mmol) in anhydrous CH_3CN (3 mL). The resulting reaction mixture was stirred at 45 ^oC for 1.5 h.

In the case of reaction of azide **7** with aziridines, progress of the reaction was monitored by TLC, and after completion of the reaction time, the solvent was removed under vacuum. The crude product was purified by column chromatography eluting with *n*-hexane/EtOAc.

Whereas in the reactions of azide **7** with 2*H*-azirines, the solid product was precipitated out during the reaction time, and this first fraction of the product was

collected by filtration after completion of the reaction time and washed with minimum amount of CH_2Cl_2 (using excess of CH_2Cl_2 will lower the yield) to remove impurities, if any. The filtrate was evaporated under reduced pressure, and to the residue obtained was added limited amount of CH_2Cl_2 and filtered to get second fraction of the product. The product on the filter was washed with CH_2Cl_2, and both the fractions were combined and dried under vacuum.

Note-1: Reaction of one equivalent of azide **7** with two equivalents of an aziridine/2*H*-azirine or vice versa did not affect the yield of the product much. In most cases, the azide **7** was used in excess.

Note-2: In the case of reactions of azide **7** with aziridines/2*H*-azirines, also avoid the addition of pure liquid aziridine/2*H*-azirine substrate to the solid azide **7**. Because, the azide **7** is being highly reactive towards strained compounds it will lead to explosion. Always, add the solution of strained compound to the solution of azide **7** or vice versa at RT or at 0 ^oC.

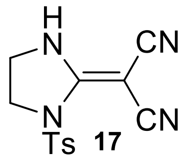
17
46%

Reaction of azide 7 with *N*-tosylaziridine (16): Azide **7** (200.0 mg, 1.68 mmol) was treated with *N*-tosylaziridine **(16)** (165.6 mg, 0.84 mmol) in anhydrous CH_3CN (10 mL) following the general procedure-II. The crude product **17** was purified by column chromatography eluting with *n*-hexane/EtOAc. Light yellow colored solid (111.4 mg, 46%), m.p. 162–163 ^oC (recrystallized from EtOAc). IR (KBr): $\tilde{\nu}$ = 3164 (br), 2223 (m), 1605 (s) cm^{-1}. ^1H NMR (400 MHz, DMSO-d6): δ = 2.43 (s, 3H, CH_3), 3.09 (t, J = 7.8 Hz, 2H, CH_2), 4.11 (t, J = 7.8 Hz, 2H, CH_2), 7.52 (d, J = 8.4 Hz, 2H, Ar-H), 7.86 (d, J = 8.4 Hz, 2H, Ar-H), 9.78 (s, 1H, NH). ^{13}C NMR (100.6 MHz, DMSO-d6): δ = 21.13 (q, CH_3), 39.76 (s, *C*–CN), 42.29 (t, CH_2), 50.08 (t, CH_2), 115.80 (br s, CN), 127.34 (d, Ar-CH), 130.43 (d, Ar-CH), 133.83 (s, Ar-C), 145.91 (s, Ar-C), 162.37 (s, *C*=C(CN)2). Elemental analysis: $C_{13}H_{12}N_4O_2S$ (288.32): calcd. C 54.15, H 4.20, N 19.43, S 11.12; found: C 54.27, H 4.33, N 19.04, S 11.25%.

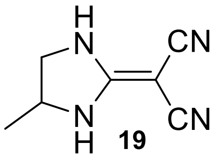
19
69%

Reaction of azide 7 with 2-methylaziridine (18): Azide **7** (200.0 mg, 1.68 mmol) was treated with 2-methylaziridine **(18)** (191.7 mg, 3.36 mmol) in anhydrous CH_3CN (10 mL) following the general procedure-II. The crude product **19** was purified by column chromatography eluting with *n*-hexane/EtOAc. The product **19** is a white solid (171.0

mg, 69%), m.p. 46–47 °C. IR (KBr): ṽ = 3161 (br), 2221 (m), 1603 (s) cm^{-1}. ^{1}H NMR (400 MHz, $CDCl_3$): *δ* = 1.26 (d, *J* = 6.4 Hz, 3H, CH_3), 3.30–3.51 (m, 3H, CH_2, CH), 7.36 (s, 1H, NH), 7.40 (s, 1H, NH). ^{13}C NMR (100.6 MHz, $CDCl_3$): *δ* = 17.60 (q, CH_3), 50.11 (s, *C*–CN), 55.56 (t, CH_2), 56.11 (d, CH), 113.86 (s, CN), 115.57 (s, CN), 160.25 (s, *C*=C(CN)$_2$). HRMS (ESI): *m/z* calcd. for $C_7H_7N_4$ ([M–H]$^+$): 147.0671, found: 147.0665.

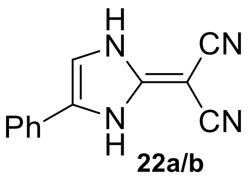

22a/b

Reaction of azide 7 with 3-phenyl-2*H*-azirine (21a): Following the general procedure-II, azide **7** (200.0 mg, 1.68 mmol) was treated with 3-phenyl-2*H*-azirine **(21a)**[S-11] (98.4 mg, 0.84 mmol) in anhydrous CH_3CN (10 mL). The product **22a** was purified as described in general procedure-II. The product **22a** is a brown colored powder (101.4 mg, 58%), looks like dust (not crystalline), and hence no sharp m.p. was observed. It started decomposing at 270 °C. IR (KBr): ṽ = 3021 (w), 2210 (s), 2162 (s), 1648 (m), 1603 (m) cm^{-1}. ^{1}H NMR (400 MHz, DMSO-d$_6$): *δ* = 7.30 (tt, *J* = 7.2 Hz, *J* = 1.5 Hz, 1H, Ar-H$_{para}$), 7.40 (td, *J* = 7.6 Hz, *J* = 1.6 Hz, 2H, Ar-H$_{meta}$), 7.53 (t, *J* = 2.0 Hz, 1H, CH), 7.74 (dd, *J* = 8.0 Hz, *J* = 1.2 Hz, 2H, Ar-H$_{ortho}$), 12.49 (s, 1H, 1-NH), 12.68 (s, 1H, 3-NH). ^{13}C NMR (100.6 MHz, DMSO-d$_6$): *δ* = 24.46 (s, *C*–CN), 112.64 (d, CH), 119.50 (s, CN), 124.85 (d, Ar-CH$_{ortho}$), 127.70 (s, *C*–Ph), 127.90 (d, Ar-CH$_{para}$), 128.67 (d, Ar-CH$_{meta}$), 129.71 (s, Ar-C$_{ipso}$), 149.25 (s, *C*=C(CN)$_2$). ^{15}N NMR (40.5 MHz, DMSO-d$_6$): *δ* = –238.58 (d, ^{1}J = 96.1 Hz, N-3), –233.45 (d, ^{1}J = 96.8 Hz, N-1), –113.33 (s, 2N, CN). HRMS (ESI): *m/z* calcd. for $C_{12}H_9N_4$ ([M+H]$^+$): 209.0827, found: 209.0822; calcd. for $C_{12}H_8N_4Na$ ([M+Na]$^+$): 231.0647, found: 231.0641; calcd. for $C_{12}H_8N_4K$ ([M+K]$^+$): 247.0386, found: 247.0381.

Reaction of azide 7 with 2-phenyl-2*H*-azirine (21b):

2-Phenyl-2*H*-azirine **(21b)** was synthesized as follows by known literature methods with some modifications and then reacted with azide **7**.

Synthesis of (2-azido-1-iodoethyl)benzene: Followed an analogous procedure[S-12a] from literature with some modifications.

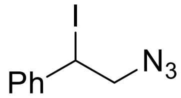

To a suspension of freshly distilled styrene (4.10 g, 40.00 mmol), sodium azide (2.60 g, 40.00 mmol), and sodium iodide (6.00 g, 40.00 mmol) in anhydrous methanol (40 mL) at 0 °C, was added dropwise a

solution of CAN (cerium ammonium nitrate) (46.00 g, 42.00 mmol) in anhydrous methanol (200 mL). The reaction mixture was stirred at 0 ^{o}C for 3 h and then saturated $NaHSO_3$ (200 mL) solution was added. The reaction mixture was extracted with CH_2Cl_2 (6x100 mL). The organic phase was washed with distilled water (200 mL) followed by brine (200 mL). The organic phase was dried over $MgSO_4$, and then the solvent was removed under vacuum at RT (the flask was wrapped with aluminium foil and shielding protector was used in front of rotavap). The crude product (orange colored oily liquid) was purified by filtration through silica gel and eluting with Et_2O:*n*-hexane = 1:10. The resulting product was used for the next step without further purification. The yield of the product was 75% (8.00 g). ^{1}H NMRs (400 MHz, $CDCl_3$): δ = 3.92 (d, ^{3}J = 7.6 Hz, 2H, CH_2), 5.14 (t, ^{3}J = 7.8 Hz, 1H, CH), 7.29–7.35 (m, 3H, Ar-H), 7.41 (m, 2H, Ar-H). ^{13}C NMR (100.6 MHz, $CDCl_3$): δ = 27.89 (d, CH), 58.60 (t, CH_2), 127.49 (d, Ar-CH), 128.72 (d, Ar-CH_{para}), 128.93 (d, Ar-CH) 140.14 (s, Ar-C_{ipso}).

Synthesis of (2-azidovinyl)benzene: (2-Azidovinyl)benzene is a known molecule in literature.[S-12b]

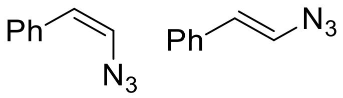

To a solution of (2-azido-1-iodoethyl)benzene (obtained above) (8.00 g, 0.03 mol) in anhydrous methanol (50 mL), was added dropwise a solution of potassium hydroxide (10.20 g, 0.18 mol) in anhydrous methanol (100 mL) over 15 min. The reaction mixture was stirred at 70 ^{o}C for 3 h, cooled, and water (300 mL) was added. The reaction mixture was extracted with $CHCl_3$ (3x200 mL). The organic phase was dried over $MgSO_4$ and the solvent was removed under vacuum at RT (shielding protector was used in front of rotavap). The crude product was purified by column chromatography eluting with *n*-hexane. The mixture of *cis* and *trans* isomers was stored in refrigerator. The yield of *cis* and *trans* isomers together was 58% (2.50 g).

***cis*-Isomer:** ^{1}H NMR (400 MHz, CD_3CN): δ = 5.72 (d, J = 8.8 Hz, 1H, CH), 6.50 (d, J = 8.4 Hz, 1H, CH), 7.20–7.25 (m, 3H, Ar-H), 7.61 (d, J = 7.8 Hz, 2H, Ar-H). ^{13}C NMR (100.6 MHz, CD_3CN): δ = 117.83 (d, CH), 126.87 (d, Ar-CH), 128.07 (d, CH), 129.22 (d, Ar-CH_{para}), 129.73 (d, Ar-CH) 135.81 (s, Ar-C_{ipso}).

***trans*-Isomer:** ^{1}H NMR (400 MHz, CD_3CN): δ = 6.27 (d, J = 14.0 Hz, 1H, CH), 6.90 (d, J = 14.0 Hz, 1H, CH), 7.29–7.35 (m, 5H, Ar-H). ^{13}C NMR (100.6 MHz, CD_3CN): δ

= 119.87 (d, CH), 126.71 (d, Ar-CH), 128.18 (d, Ar-CH_{para}), 128.34 (d, CH), 129.68 (d, Ar-CH) 136.28 (s, Ar-C_{ipso}).

Synthesis of 2-phenyl-2*H*-azirine (21b): 2-Phenyl-2*H*-azirine **(21b)** is a known molecule in literature.[S-12c,d]

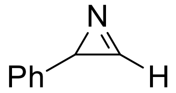

A solution of (2-azidovinyl)benzene (obtained above) (100.0 mg, 0.69 mmol) in CD_3CN (0.7 mL) in NMR tube was photolyzed at –40 °C for 1.5 h. NMR data of the sample were measured to confirm the complete conversion of precursor to the azirine **21b**. ^{1}H NMR yield of the azirine **21b** was 80% (64.6 mg). Solution of azirine **21b** in CD_3CN was directly used to react with azide **7**. ^{1}H NMR (400 MHz, CD_3CN): δ = 2.81 (d, J = 2.4 Hz, 1H, 2-H), 7.10 (m, 2H, Ar-H), 7.26–7.35 (m, 3H, Ar-H), 10.07 (d, J = 2.4 Hz, 1H, 3-H). ^{13}C NMR (100.6 MHz, CD_3CN): δ = 29.05 (d, C-2), 126.87 (d, Ar-CH), 127.96 (d, Ar-CH_{para}), 129.18 (d, Ar-CH), 142.55 (s, Ar-C_{ipso}), 160.81 (d, C-3).

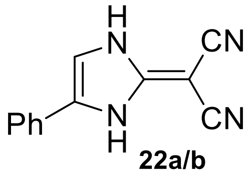

22a/b

Reaction of azide 7 with 2-phenyl-2*H*-azirine (21b): Azide **7** (196.5 mg, 1.65 mmol) in anhydrous CH_3CN (10 mL) was treated with a solution of 2-phenyl-2*H*-azirine **(21b)** (64.6 mg, 0.55 mmol) (obtained above directly from the photolysis of (2-azidovinyl)benzene in CD_3CN) under inert atmospheric conditions following the general procedure-II. The product was purified as described in general procedure-II. In this reaction, the same product **22a/b** (70.0 mg, 61%) was obtained as in the case of reaction of **7** with **21a**.

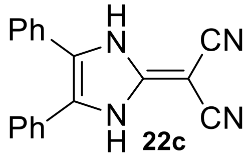

22c
56%

Reaction of azide 7 with 2,3-diphenyl-2*H*-azirine (21c): Following the general procedure-II, the azide **7** (200.0 mg, 1.68 mmol) was treated with 2,3-phenyl-2*H*-azirine **(21c)** (162.2 mg, 0.84 mmol) in anhydrous CH_3CN (10 mL). The product **22c** was purified as described in general procedure-II. The product **22c** is a green colored powder (133.4 mg, 56%), looks like dust (not crystalline), and hence no sharp m.p. was observed. It started decomposing at 245 °C. IR (KBr): $\tilde{\nu}$ = 3019 (w), 2216 (s), 2168 (s), 1651 (m), 1607 (s) cm^{-1}. ^{1}H NMR (400 MHz, DMSO-d_6): δ = 7.36 (s, 10H, Ar-H), 12.83 (s, 2H, NH). ^{13}C NMR (100.6 MHz, DMSO-d_6): δ = 24.34 (s, *C*–CN), 119.46 (s, CN), 125.16 (s, *C*–Ph or Ar-C_{ipso}), 127.87 (s, *C*–Ph or Ar-C_{ipso}), 128.45 (d, Ar-CH), 128.50 (d, Ar-CH), 128.57 (d, Ar-C_{para}), 148.64 (s, *C*=C(CN)$_2$). ^{15}N NMR

(40.5 MHz, DMSO-d_6): δ = –232.22 (dd, ^1J = 96.2 Hz, J = 2.7 Hz, NH), –113.37 (s, 2N, CN). HRMS (ESI): *m/z* calcd. for $C_{18}H_{13}N_4$ ([M+H]$^+$): 285.1140, found: 285.1135; calcd. for $C_{18}H_{12}N_4Na$ ([M+Na]$^+$): 307.0960, found: 307.0954.

Reaction of azide 7 with 2-methyl-3-phenyl-2*H*-azirine (21d): Following the general procedure-II, azide **7** (272.4 mg, 2.28 mmol) was treated with 2-methyl-3-phenyl-2*H*-azirine **(21d)** (150.0 mg, 1.14 mmol) in anhydrous CH_3CN (10 mL). The product **22d** was purified as described in general procedure-II. The product **22d** is a yellowish brown colored powder (159.3 mg, 62%), looks like dust and hence no sharp m.p. was observed. It started decomposing at 260 °C. IR (KBr): ṽ = 3009 (w), 2171 (s), 1649 (m) cm^{-1}. ^{1}H NMR (400 MHz, DMSO-d_6): δ = 2.22 (s, 3H, CH_3), 7.33 (t, J = 7.0 Hz, 1H, Ar-H_{para}), 7.43 (t, J = 7.4 Hz, 2H, Ar-H_{meta}), 7.49 (d, J = 7.2 Hz, 2H, Ar-H_{ortho}), 12.37 (s, 1H, NH), 12.49 (s, 1H, NH). ^{13}C NMR (100.6 MHz, DMSO-d_6): δ = 9.90 (q, CH_3), 23.98 (s, *C*–CN), 119.74 (s, CN), 121.80 (s, *C*–CH_3 or *C*–Ph), 124.33 (s, *C*–CH_3 or *C*–Ph), 127.16 (d, Ar-CH), 127.69 (d, Ar-CH_{para}), 128.21 (s, Ar-C_{ipso}), 128.57 (d, Ar-CH), 147.60 (s, *C*=C(CN)$_2$). Elemental analysis: $C_{13}H_{10}N_4$ (222.24): calcd. C 70.26, H 4.54, N 25.21; found: C 69.82, H 4.54, N 25.24%.

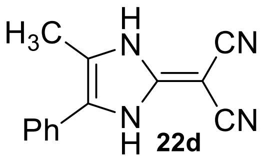

22d
62%

Reaction of azide 7 ^{15}N-labeled 3-phenyl-2*H*-azirine (^{15}N-21a):

^{15}N-Labeled 3-phenyl-2*H*-azirine **(^{15}N-21a)** was synthesized by an analogous procedure[S-11] from literature and then reacted with azide **7**.

A solution of bromine (326.4 mg, 4.08 mmol) in absolute CCl_4 (5 mL) was added to a cooled (15–20 °C) solution of distilled styrene (212.2 mg, 2.03 mmol) in anhydrous CCl_4 (5 mL). The reaction mixture was stirred for 2 h, and then the solvent was removed under vacuum to get the crystalline (1,2-dibromoethyl)benzene with an yield of 99%. (1,2-Dibromoethyl)benzene was dissolved in anhydrous DMSO (10 mL), and to this solution terminal ^{15}N-labeled sodium azide (200.0 mg, 3.07 mmol) was added at 15–20 °C under inert atmosphere. The reaction mixture became thick with precipitated (1-azido-2-bromoethyl)benzene and was stirred for another 13 h at RT. The reaction mixture was cooled to 12 °C, and was treated with a solution of NaOH (80.0 mg, 2.00 mmol) in H_2O (1 mL), and then allowed to stir at RT for another 24 h. The reaction mixture was poured into 2% $NaHCO_3$ (15 mL) solution and extracted with CH_2Cl_2. The organic phase was dried over $MgSO_4$ and then

evaporated to yield the crude product as red oil. The crude oil was purified by passing through a column of alumina using petroleum ether as an eluent. The eluate was evaporated to get the residual pale yellow oil of mixture of ^{15}N-labeled and unlabeled (1-azidovinyl)benzene (207.0 mg, 70%). Next, this mixture was dissolved in anhydrous toluene (30 mL) and the solution was refluxed for 4 h. Removal of the solvent yielded a mixture of ^{15}N-labeled and unlabeled 3-phenyl-2*H*-azirines ^{15}N-**21a** and **21a** (90.0 mg, 54%). The substrates ^{15}N-**21a** and ^{15}N-labeled (1-azidovinyl)-benzene were known in literature.[S-13]

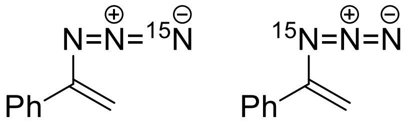

^{15}N-labeled (1-azidovinyl)benzene: ^{1}H NMR (400 MHz, $CDCl_3$): δ = 4.98 (dd, *J* = 2.4 Hz, *J* = 1.2 Hz, 1H, CH_2), 5.46 (dd, *J* = 4.4 Hz, *J* = 2.2 Hz, 1H, CH_2), 7.36–7.40 (m, 3H, Ar-H), 7.58–7.61 (m, 2H, Ar-H). ^{13}C NMR (100.6 MHz, $CDCl_3$): δ = 97.84 (t, CH_2), 125.47 (d, Ar-CH), 128.34 (d, Ar-CH), 129.01 (d, Ar-CH), 134.18 (s, Ar-C_{ipso}), 144.97 (s, *C*–Ph). ^{15}N decoupled NMR (40.5 MHz, $CDCl_3$): δ = –287.44 (s, α-^{15}N), –146.26 (s, γ-^{15}N).

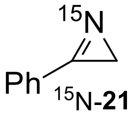

^{15}N-**21a**

^{15}N-labeled 3-phenyl-2*H*-azirine (^{15}N-21a): ^{1}H NMR (400 MHz, $CDCl_3$): δ = 1.74 (d, *J* = 2.8 Hz, 2H, CH_2), 7.47–7.56 (m, 3H, Ar-H), 7.85 (dd, *J* = 7.8 Hz, *J* = 1.8 Hz, 2H, Ar-H). ^{13}C NMR (100.6 MHz, $CDCl_3$): δ = 19.49 (t, CH_2), 125.25 (s, Ar-C_{ipso}), 128.86 (d, Ar-CH), 129.37 (d, Ar-CH), 132.73 (d, Ar-CH_{para}), 165.53 (s, *C*–Ph). ^{15}N NMR (40.5 MHz, $CDCl_3$): δ = –120.80 (s, C=^{15}N).

Azide **7** (200.0 mg, 1.68 mmol) was treated with a mixture of ^{15}N-labeled and unlabeled 3-phenyl-2*H*-azirine **(^{15}N-21a+21a)** (90.0 mg, 0.77 mmol) (obtained above) in anhydrous CH_3CN following the general procedure-II. The resulting product was purified as described in general procedure-II. The product is a mixture of two ^{15}N-labeled components (^{15}N-**22a** and ^{15}N-**22a'**) and the unlabeled component **22a**. The isolated yield of light brown colored solid mixture of **22a**+^{15}N-**22a**+^{15}N-**22a'** was 35% (56.0 mg). The ratio of ^{15}N-**22a**/^{15}N-**22a'** = 11. ^{1}H NMR (400 MHz, DMSO-d_6): δ = 7.29 (t, *J* = 7.4 Hz, 1H, Ar-H_{para}), 7.39 (t, *J* = 7.6 Hz, 2H, Ar-H_{meta}), 7.52 (t, *J* = 2.0 Hz, 1H, CH), 7.73 (d, *J* = 7.6 Hz, 2H, Ar-H_{ortho}), 12.50 (dt, ^{1}J = 97.6 Hz, *J* = 2.2 Hz, 1H, ^{15}NH **(^{15}N-22a)**), 12.50 (s, 1H, 1-NH), 12.68 (dt, ^{1}J = 96.0 Hz, *J* = 2.2 Hz, 1H, ^{15}NH **(^{15}N-22a')**), 12.68 (s, 1H, 3-NH). ^{13}C NMR (100.6 MHz, DMSO-d_6): δ = 24.49 (s, *C*–

CN), 112.68 (d, CH), 119.56 (s, CN), 124.86 (d, Ar-CH*ortho*), 127.72 (s, *C*–Ph), 127.94 (d, Ar-CH*para*), 128.71 (d, Ar-CH*meta*), 129.74 (s, Ar-C*ipso*), 149.23 (s, *C*=C(CN)2). ^{15}N NMR (40.5 MHz, DMSO-d6): δ = –238.85 (dt, ^{1}J = 96.4 Hz, ^{3}J = 3.4 Hz, ^{15}N-3 **(**^{15}N-**22a'))**, –233.56 (ddd, ^{1}J = 97.4 Hz, J = 4.6 Hz, J = 3.7 Hz, ^{15}N-1 **(**^{15}N-**22a))**.

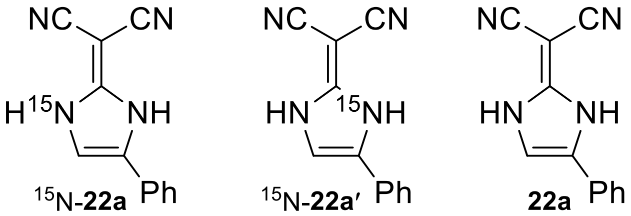

^{15}N-**22a+**^{15}N-**22a′+22a = 35%**
ratio of ^{15}N-**22a/**^{15}N-**22a′** = 11:1

Reaction of ^{15}N-labeled azide ^{15}N-**7 with 3-phenyl-2*H*-azirine (21a):**

^{15}N-Labeled azide ^{15}N-**7** (100.0 mg, 0.84 mmol) was treated with 3-phenyl-2*H*-azirine **(21a)** (196.8 mg, 1.68 mmol) in anhydrous CH_3CN (10 mL) following the general procedure-II. The resulting product was purified as described in general procedure-II. The product is a mixture of three ^{15}N-labeled components (^{15}N-**22a**, ^{15}N-**22a'**, and ^{15}N-**22a''**) and the unlabeled component **22a**. The isolated yield of light brown colored solid mixture of **12a**+^{15}N-**22a**+^{15}N-**22a'**+^{15}N-**22a''** was 33% (46.0 mg). The ratio of ^{15}N-**22a/**^{15}N-**22a'** = 1/22. ^{1}H NMR (400 MHz, DMSO-d6): δ = 7.29 (t, J = 7.4 Hz, 1H, Ar-H*para*), 7.39 (t, J = 7.6 Hz, 2H, Ar-H*meta*), 7.53 (br s, 1H, CH), 7.73 (d, J = 7.6 Hz, 2H, Ar-H*ortho*), 12.51 (br d, ^{1}J = 96.4 Hz, 1H, 1-^{15}NH **(**^{15}N-**22a))**, 12.51 (s, 1H, 1-NH), 12.69 (br d, ^{1}J = 96.4 Hz, 1H, 3-^{15}NH **(**^{15}N-**22a'))**, 12.69 (s, 1H, 3-NH). ^{13}C NMR (100.6 MHz, DMSO-d6): δ = 24.51 (s, *C*–CN), 112.70 (d, CH), 119.58 (s, CN), 124.88 (d, Ar-CH*ortho*), 127.74 (s, *C*–Ph), 127.94 (d, Ar-CH*para*), 128.72 (d, Ar-CH*meta*), 129.75 (s, Ar-C*ipso*), 149.24 (s, *C*=C(CN)2). ^{15}N coupled NMR (40.5 MHz, DMSO-d6): δ = –238.64 (dt, ^{1}J = 96.4 Hz, ^{3}J = 3.4 Hz, ^{15}N-3 **(**^{15}N-**22a'))**, –113.25 (s, C^{15}N **(**^{15}N-**22a''))**, the very low intense ^{15}N-1 signal of ^{15}N-**22a** was not observed.

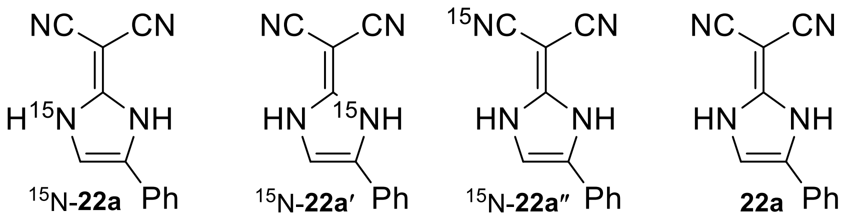

^{15}N-**22a** ^{15}N-**22a′** ^{15}N-**22a″** **22a**

^{15}N-**22a**+^{15}N-**22a′**+^{15}N-**22a″**+**22a** = 33%
ratio of ^{15}N-**22a**/^{15}N-**22a′** = 1:22

4. Synthesis and reactions of aquoethereal cyanoform 2 solution:

Aquoethereal cyanoform **2** solution was synthesized by following the literature[S-14] with some modifications.

Potassium tricyanomethanide salt (**1b**) [S-15,S-16] (2.00 g, 15.48 mmol) was dissolved in distilled water (5 mL). Diethyl ether (17 mL) was added to the top of the aqueous solution, resulting in two distinct layers. To this solution at 0 °C, was added conc. H_2SO_4 (1.52 g, 0.82 mL, 15.48 mmol) dropwise. After stirring the resulting reaction mixture at 0 °C for 1 h, an additional yellow layer appeared between the upper ether layer and the lower aqueous layer. The lower aqueous phase was separated; the middle and upper phases were used to react with epoxides and 2*H*-azirines in the next step.

Reaction of aquoethereal cyanoform 2 with cyclohexene oxide (9a):

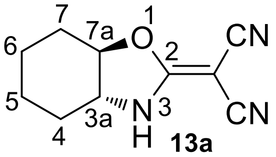

13a
75%

To the aquoethereal cyanoform **2** solution (obtained above), was added cyclohexene oxide (**9a**) (5.53 g, 5.71 mL, 56.43 mmol) dropwise at 0 °C. The product **13a** precipitated out during the addition of cyclohexene oxide (**9a**). The resulting reaction mixture was stirred at 0 °C for 1.5 h, filtered, and the solid product was thoroughly washed with diethyl ether. The crude compound is a mixture of the desired product **13a** and cyclohexane-1,2-diol. These two were separated by column chromatography eluting with *n*-hexane/EtOAc = 7:3. The yield of light yellow colored solid **13a** was 75% (2.20 g).

Reaction of aquoethereal cyanoform 2 with 3-phenyl-2*H*-azirine (21a):

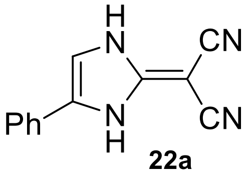

22a
10%

Aquoethereal cyanoform **2** solution was prepared by treating potassium tricyanomethanide salt (**1b**) (400.0 mg, 3.10 mmol)

in water (4 mL) with conc. H_2SO_4 (285.6 mg, 155.2 μL, 2.91 mmol) as described above. To this solution at 0 ^oC, 3-phenyl-2*H*-azirine **(21a)** (181.4 mg, 1.55 mmol) was added dropwise and stirred for 1.5 h. As the reaction progressed the product was precipitated out and after completion of the reaction, the mixture was filtered, and the solid on the filter was washed with limited amount of CH_2Cl_2. The yield of light brown colored solid **22a** was 10% (32.0 mg).

5. Direct reaction of potassium tricyanomethanide salt (1b) with cyclohexene oxide (9a) in presence of conc. H_2SO_4:

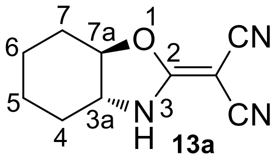

13a
79%

To a solution of potassium tricyanomethanide salt (**1b**) (100.0 mg, 0.77 mmol) in DME (dimethoxyethane) (10 mL) was added cyclohexene oxide (**9a**) (151.9 mg, 1.54 mmol). To this solution at 0 ^oC, was added conc. H_2SO_4 (100.0 mg, 1.01 mmol) dropwise. After addition of a drop of conc. H_2SO_4, the reaction mixture was turned to yellow. Stirring was continued for another 15 min. The reaction mixture was filtered, and the filtrate was concentrated under reduced pressure. To the residue obtained, a small amount of CH_2Cl_2 was added, filtered, and washed with little amount of CH_2Cl_2 and dried under vacuum. The yield of light yellow colored solid product **13a** was 79% (116.0 mg).

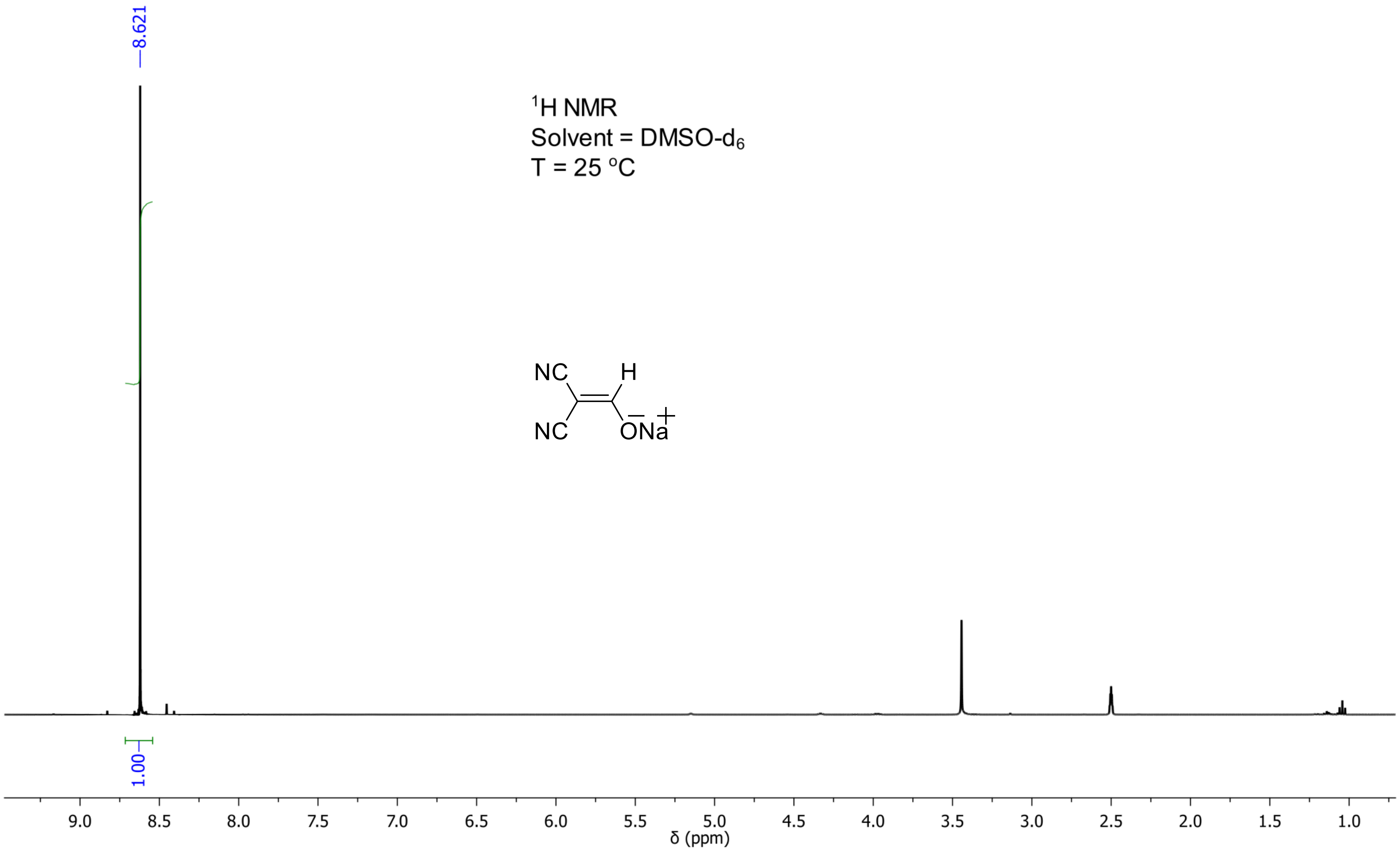

8.621
1H NMR
Solvent = DMSO-d6
T = 25 °C
NC
H
NC
ONa
1.00
9.0
8.5
8.0
7.5
7.0
6.5
6.0
5.5
5.0
4.5
4.0
3.5
3.0
2.5
2.0
1.5
1.0
δ (ppm)

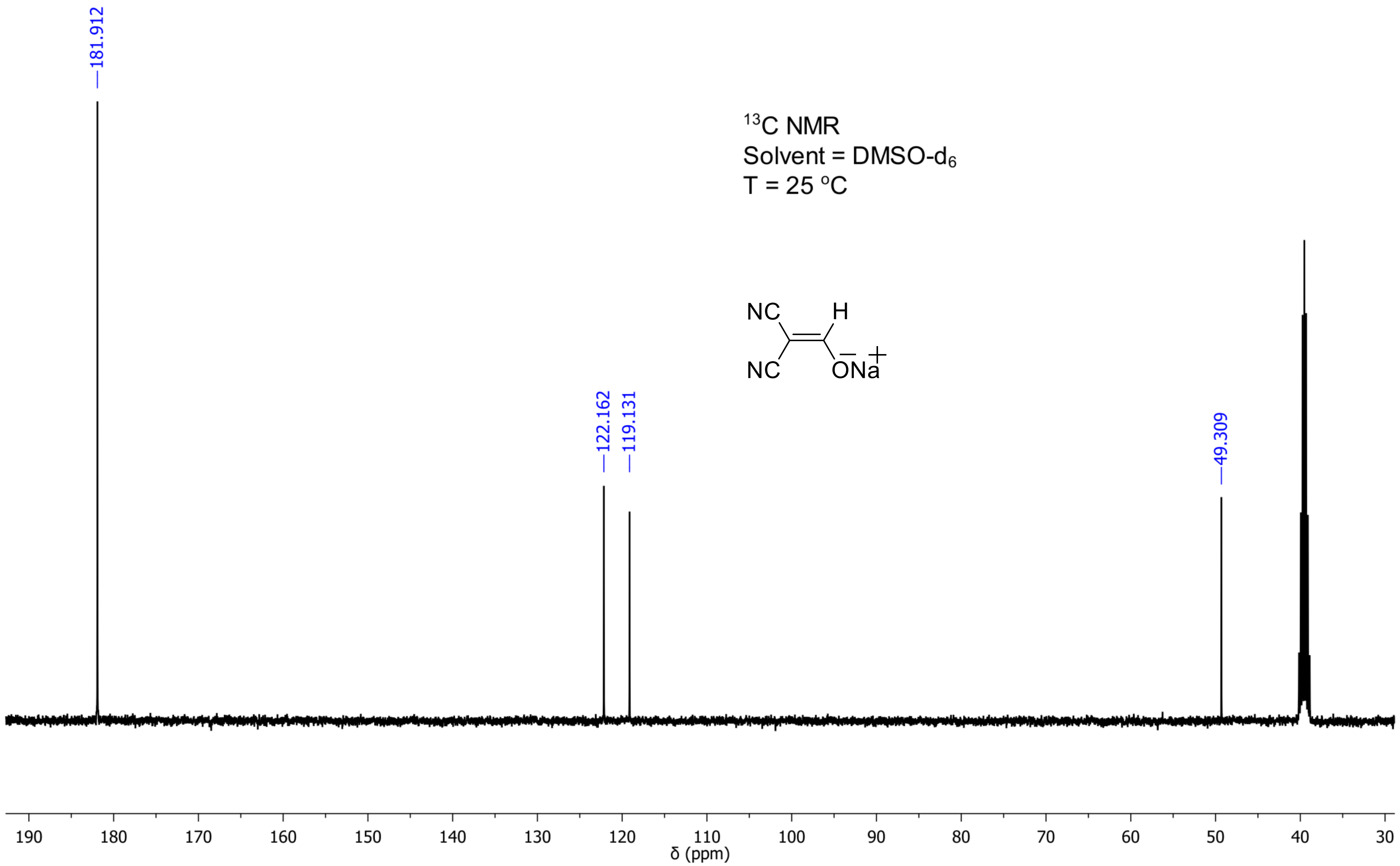

181.912
^{13}C NMR
Solvent = DMSO-d$_6$
T = 25 °C
NC
H
NC
ONa
122.162
119.131
49.309
190
180
170
160
150
140
130
120
110
100
90
80
70
60
50
40
30
δ (ppm)

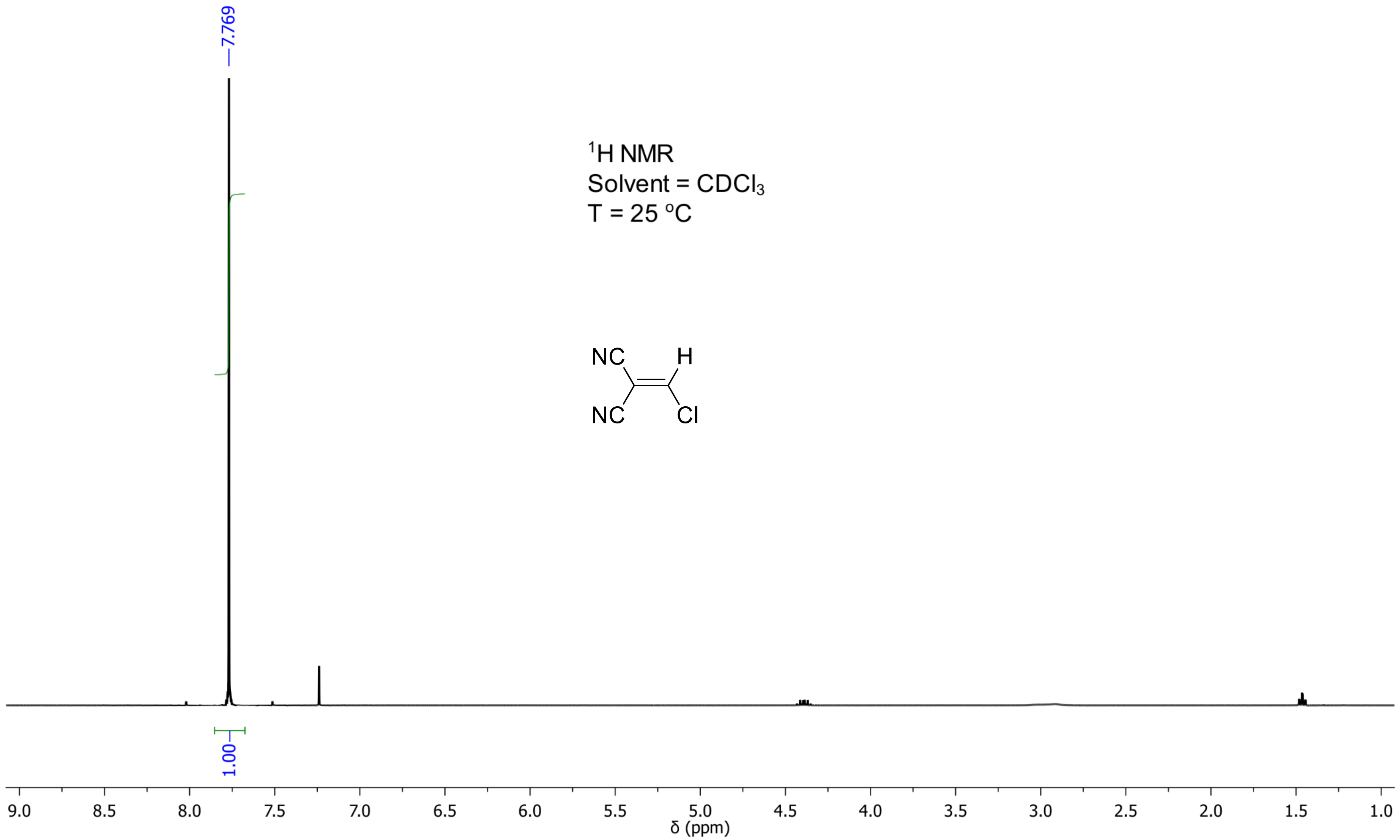
7.769
1H NMR
Solvent = CDCl3
T = 25 °C
NC
H
NC
Cl
1.00
9.0
8.5
8.0
7.5
7.0
6.5
6.0
5.5
5.0
4.5
4.0
3.5
3.0
2.5
2.0
1.5
1.0
δ (ppm)

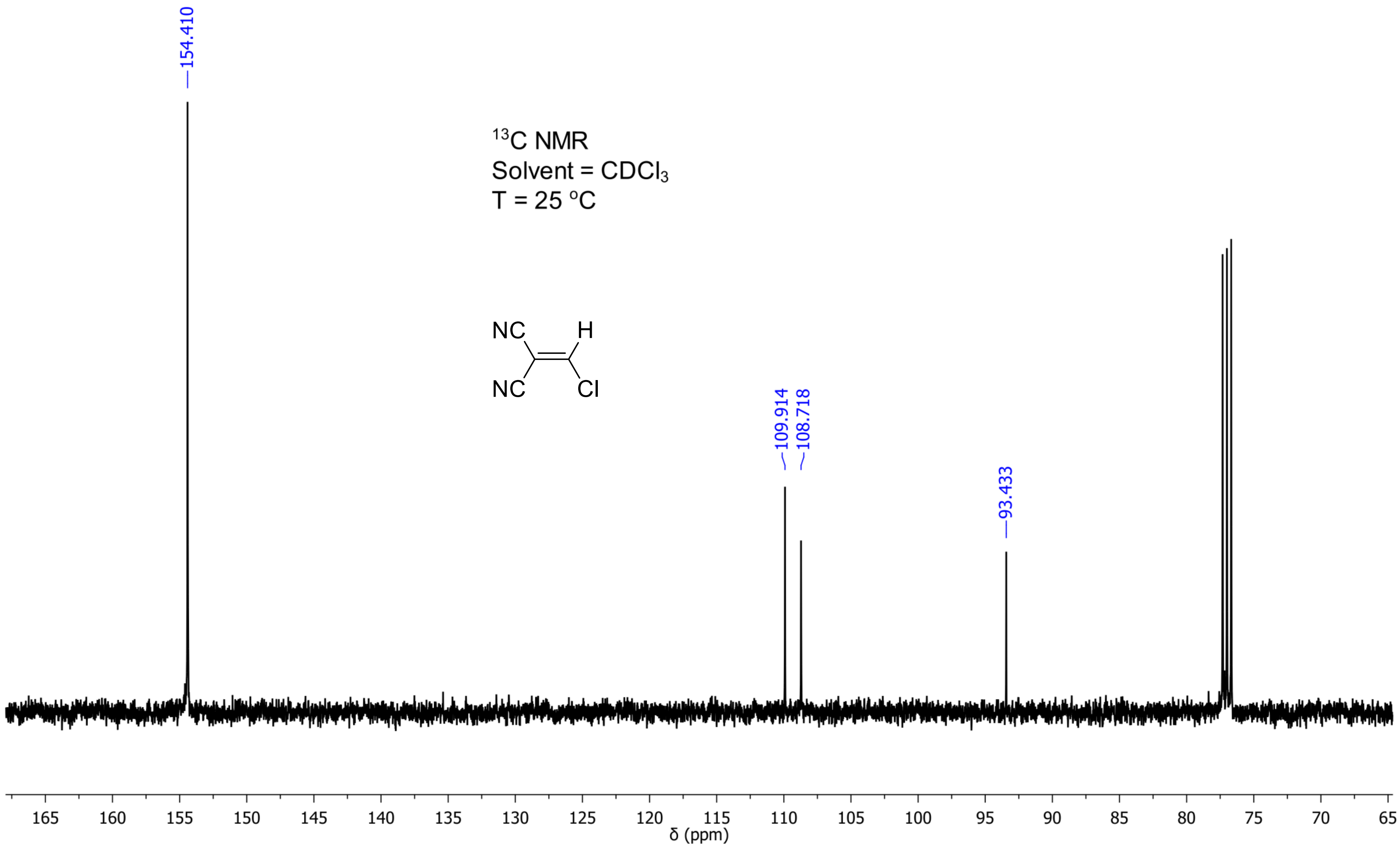

154.410
^{13}C NMR
Solvent = CDCl$_3$
T = 25 °C
NC
H
NC
Cl
109.914
108.718
93.433
165
160
155
150
145
140
135
130
125
120
115
110
105
100
95
90
85
80
75
70
65
δ (ppm)

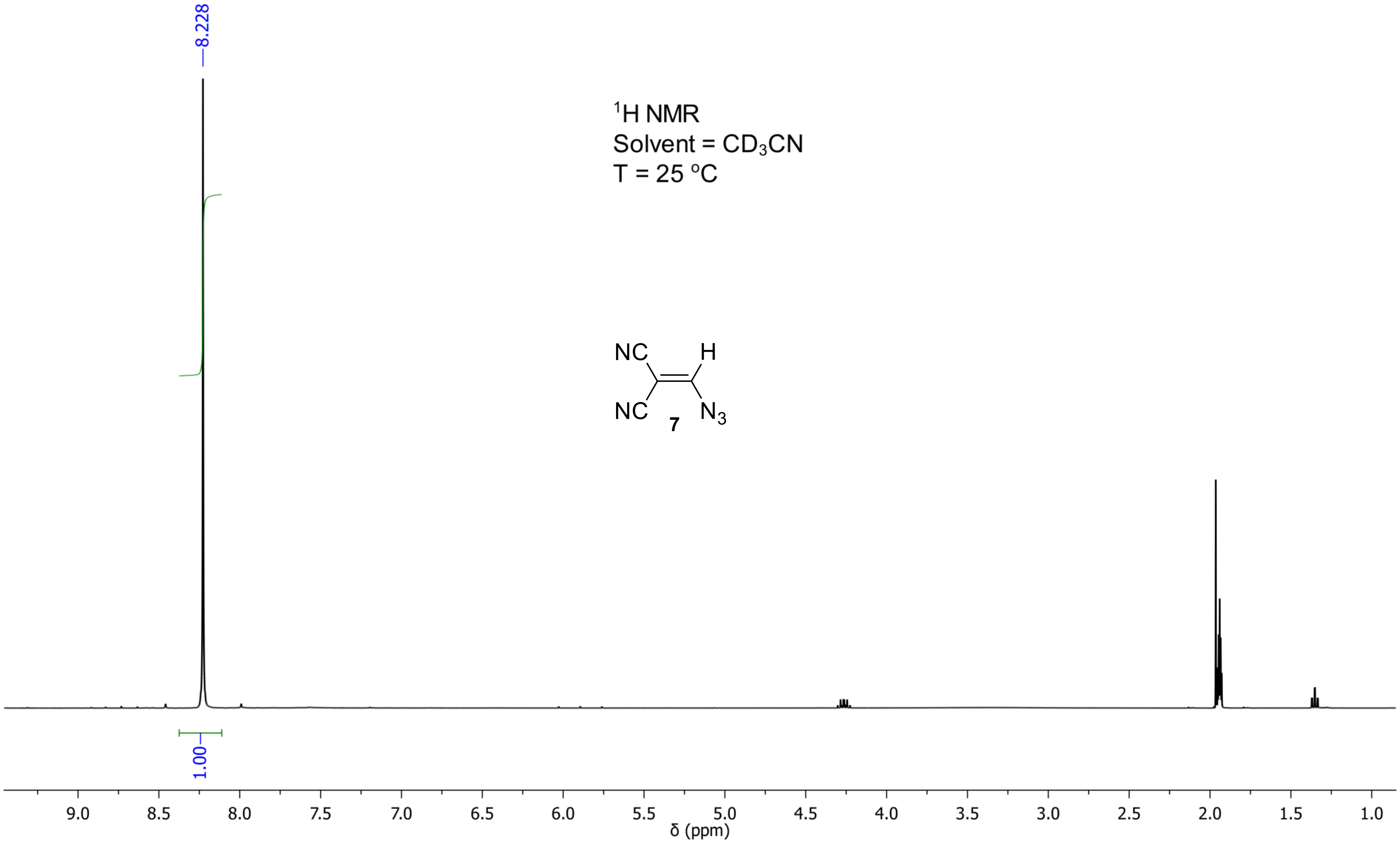

^{1}H NMR
Solvent = CD_3CN
T = 25 °C

7

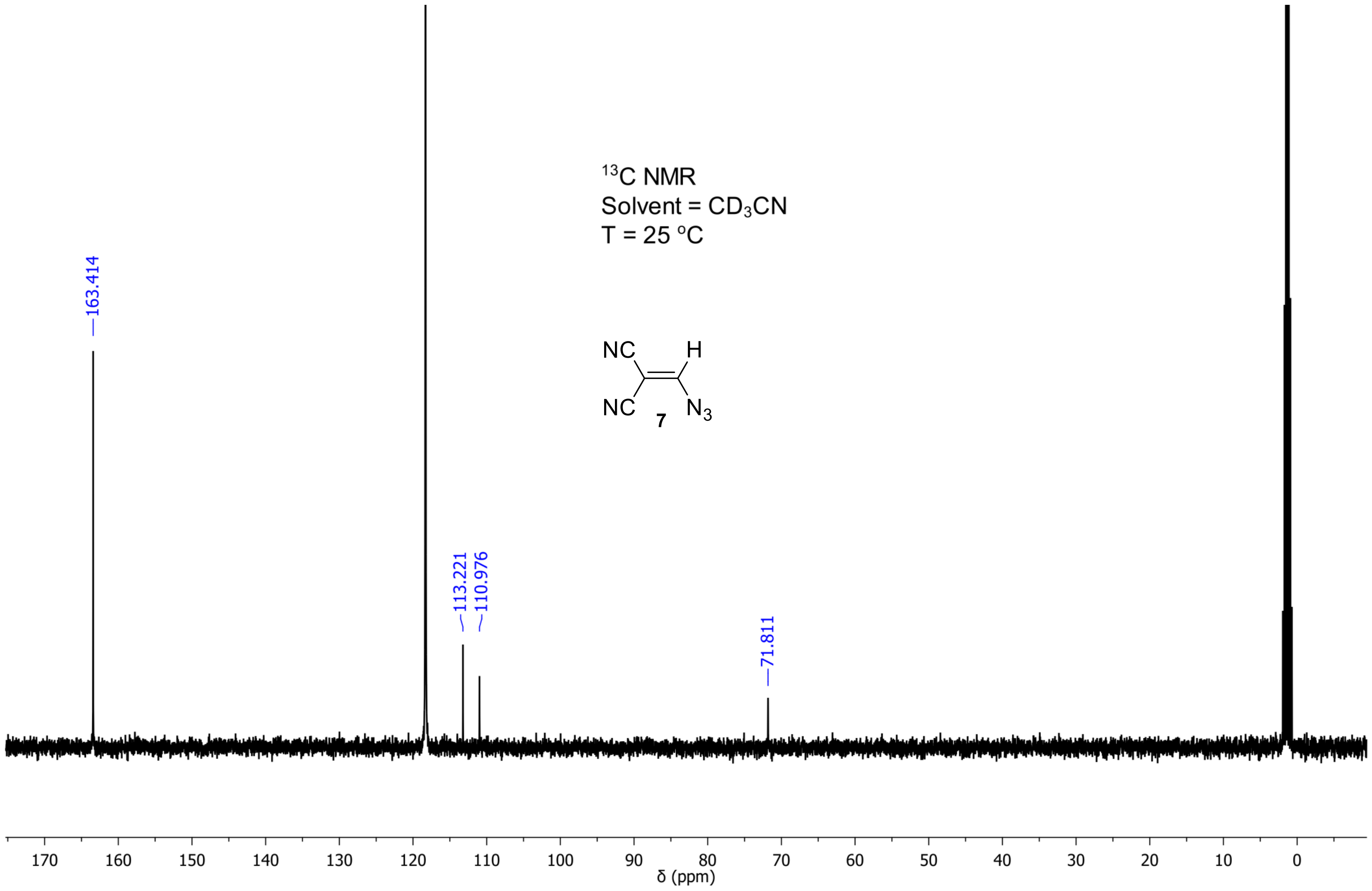

13C NMR
Solvent = CD3CN
T = 25 °C
NC
H
NC
N3
7
163.414
113.221
110.976
71.811
170
160
150
140
130
120
110
100
90
80
70
60
50
40
30
20
10
0
δ (ppm)

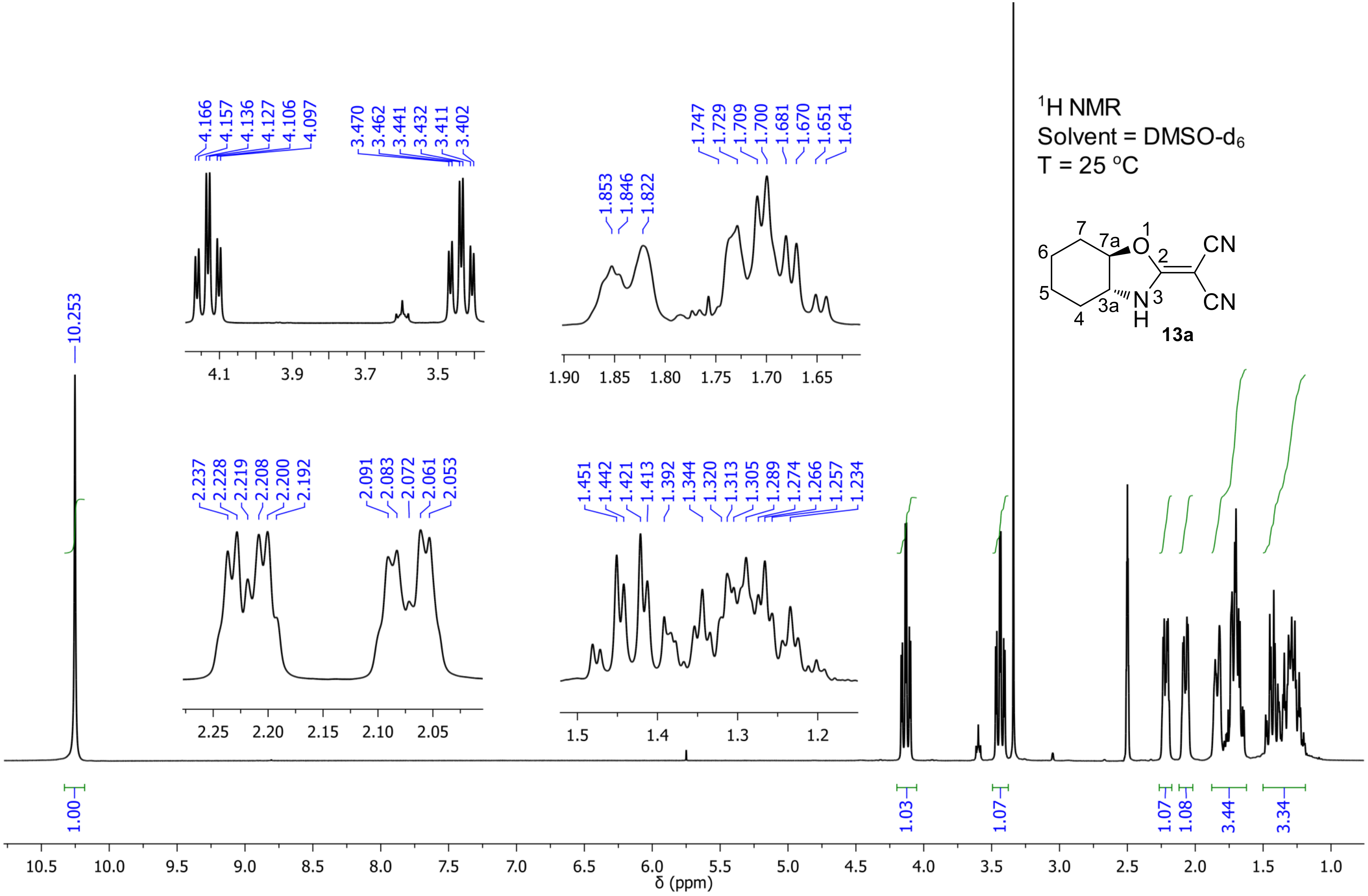

^{1}H NMR
Solvent = DMSO-d_6
T = 25 °C
13a
CN
CN
O
N
H
10.253
4.166 4.157 4.136 4.127 4.106 4.097
3.470 3.462 3.441 3.432 3.411 3.402
1.853 1.846 1.822
1.747 1.729 1.709 1.700 1.681 1.670 1.651 1.641
2.237 2.228 2.219 2.208 2.200 2.192
2.091 2.083 2.072 2.061 2.053
1.451 1.442 1.421 1.413 1.392 1.344 1.320 1.313 1.305 1.289 1.274 1.266 1.257 1.234
1.00 1.03 1.07 1.07 1.08 3.44 3.34
δ (ppm)

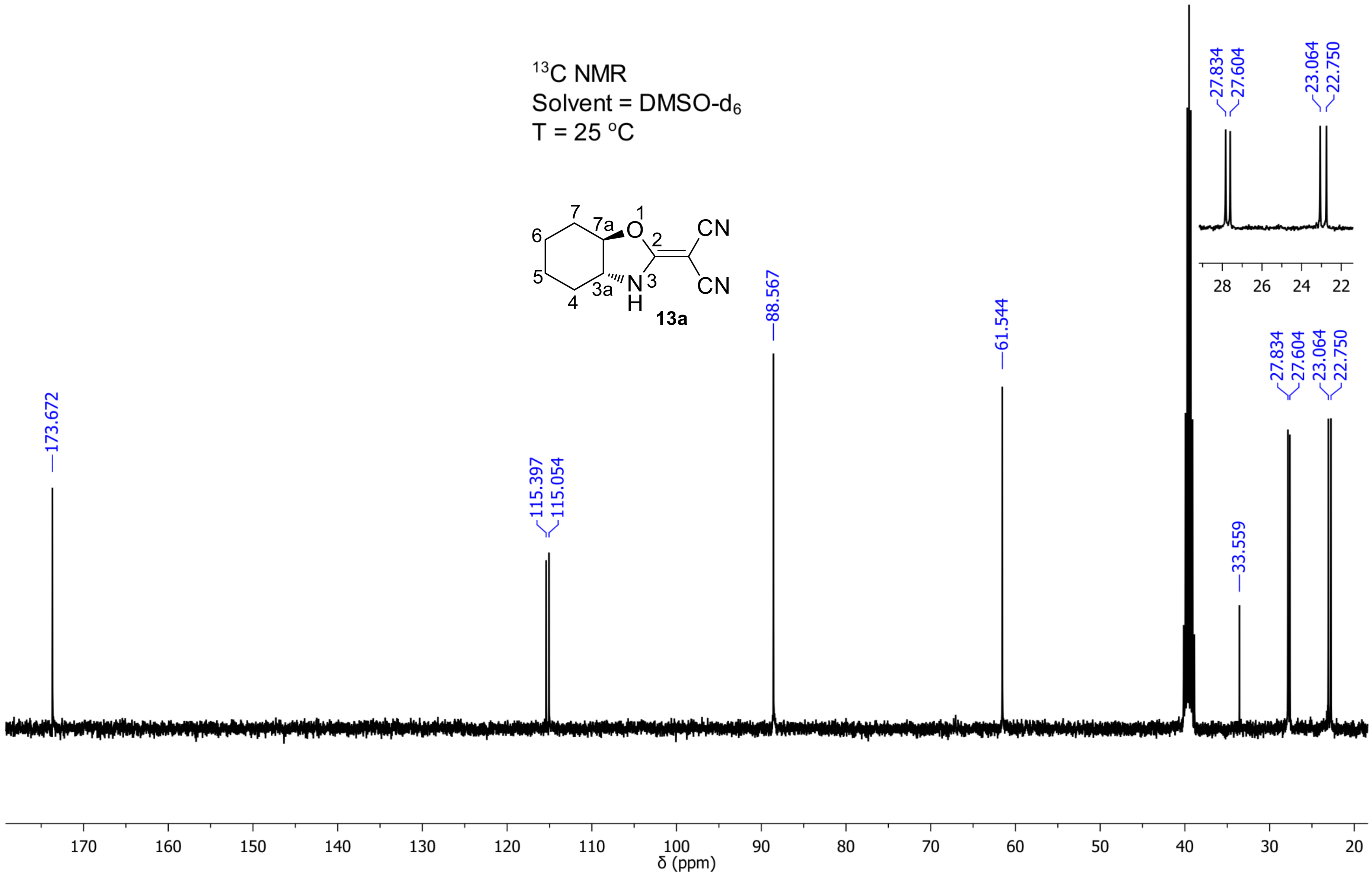

13C NMR
Solvent = DMSO-d6
T = 25 °C
7
7a
O
1
6
2
CN
5
3a
N
3
CN
4
H
13a
173.672
115.397
115.054
88.567
61.544
33.559
27.834
27.604
23.064
22.750
28
26
24
22
170
160
150
140
130
120
110
100
90
80
70
60
50
40
30
20
δ (ppm)

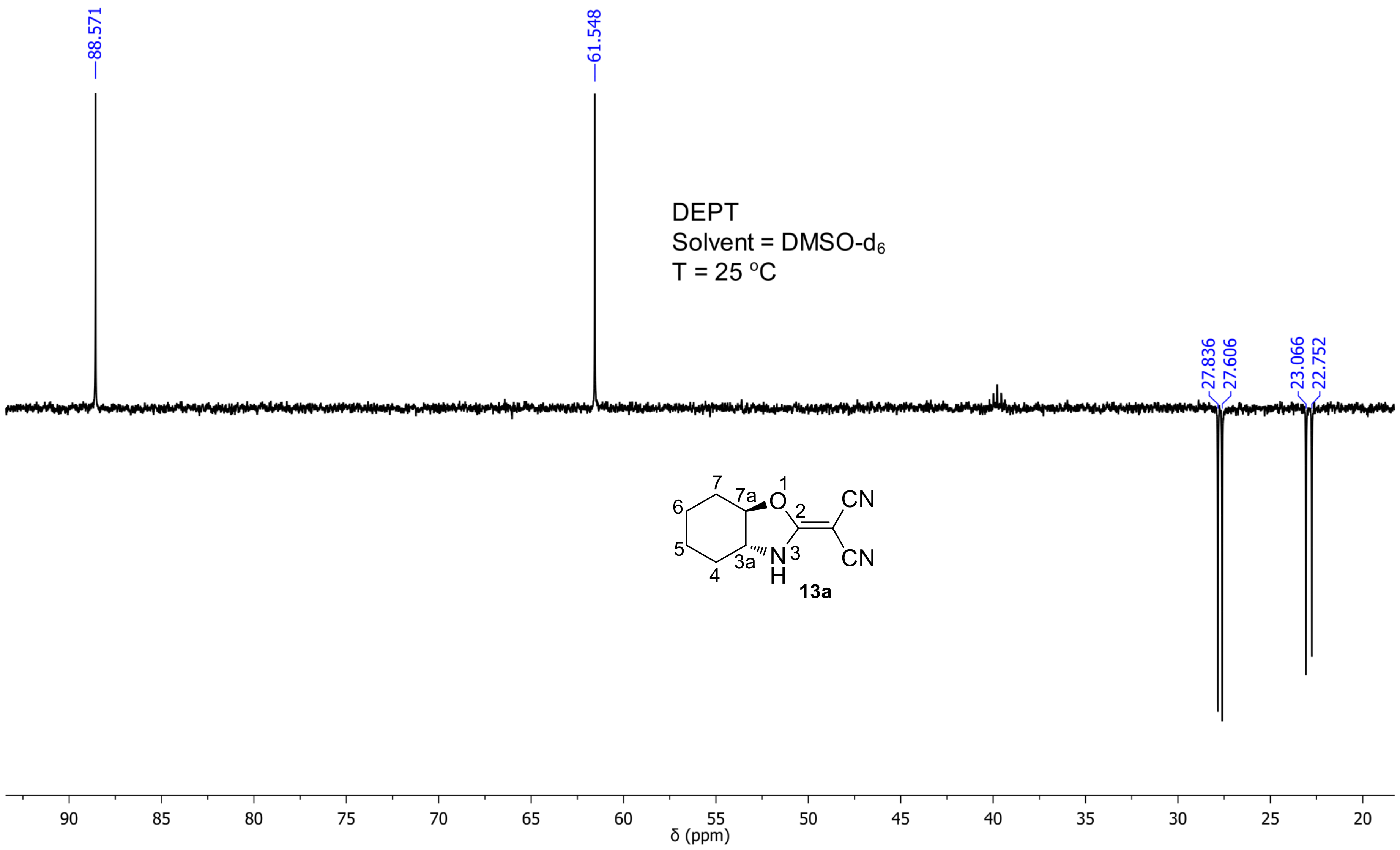
88.571
61.548
27.836
27.606
23.066
22.752
DEPT
Solvent = DMSO-d_6
T = 25 °C
7
1
7a
O
2
CN
6
5
3a
N
3
CN
H
4
13a
90
85
80
75
70
65
60
55
50
45
40
35
30
25
20
δ (ppm)

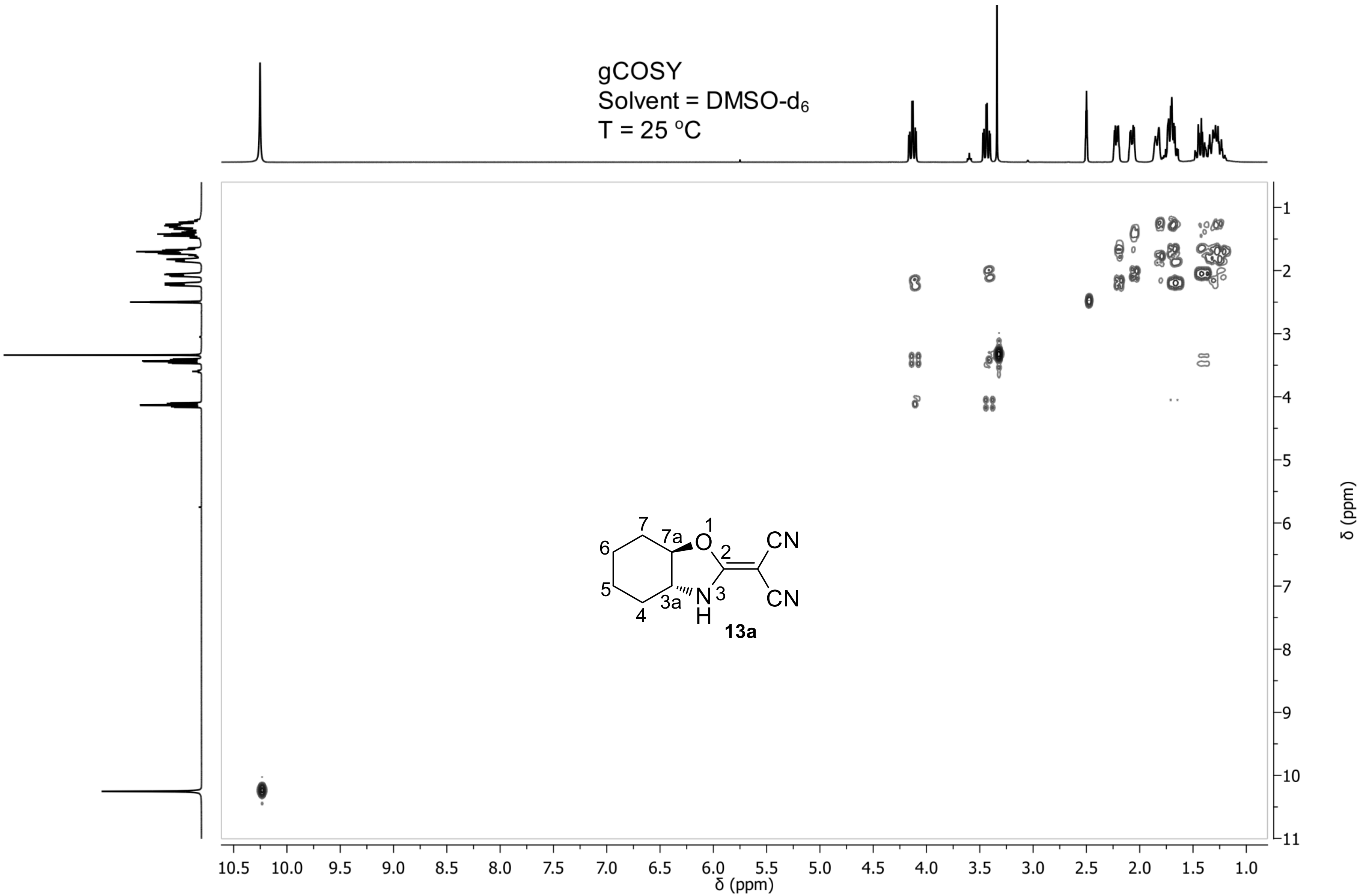
gCOSY
Solvent = DMSO-d6
T = 25 °C
7
7a
1
6
O
2
CN
5
N
3
3a
H
CN
4
13a
δ (ppm)
δ (ppm)

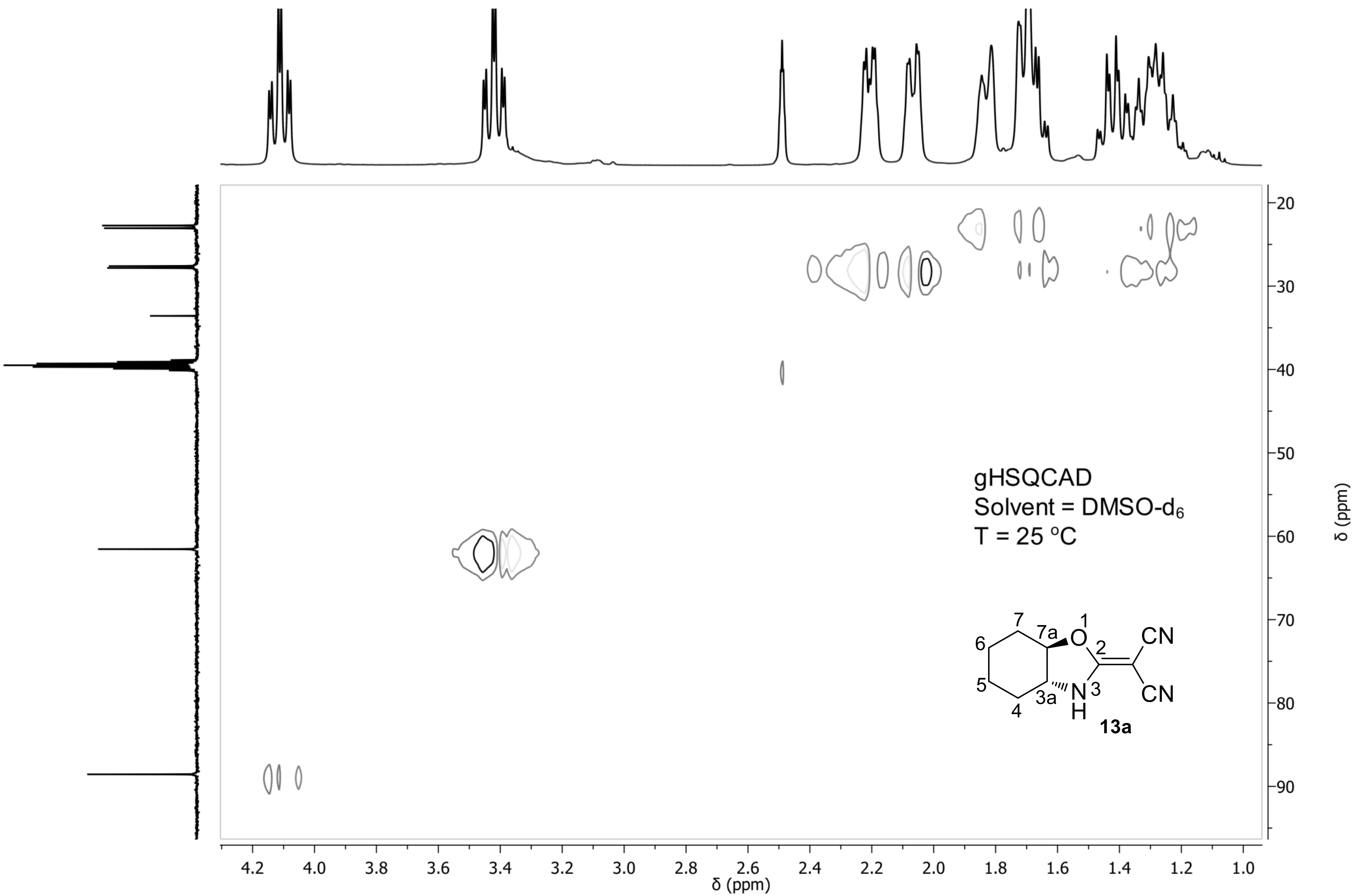
gHSQCAD
Solvent = DMSO-d6
T = 25 °C
7
7a
O
1
2
CN
6
5
3a
N
3
CN
H
4
13a
4.2
4.0
3.8
3.6
3.4
3.2
3.0
2.8
2.6
2.4
2.2
2.0
1.8
1.6
1.4
1.2
1.0
δ (ppm)
20
30
40
50
60
70
80
90
δ (ppm)

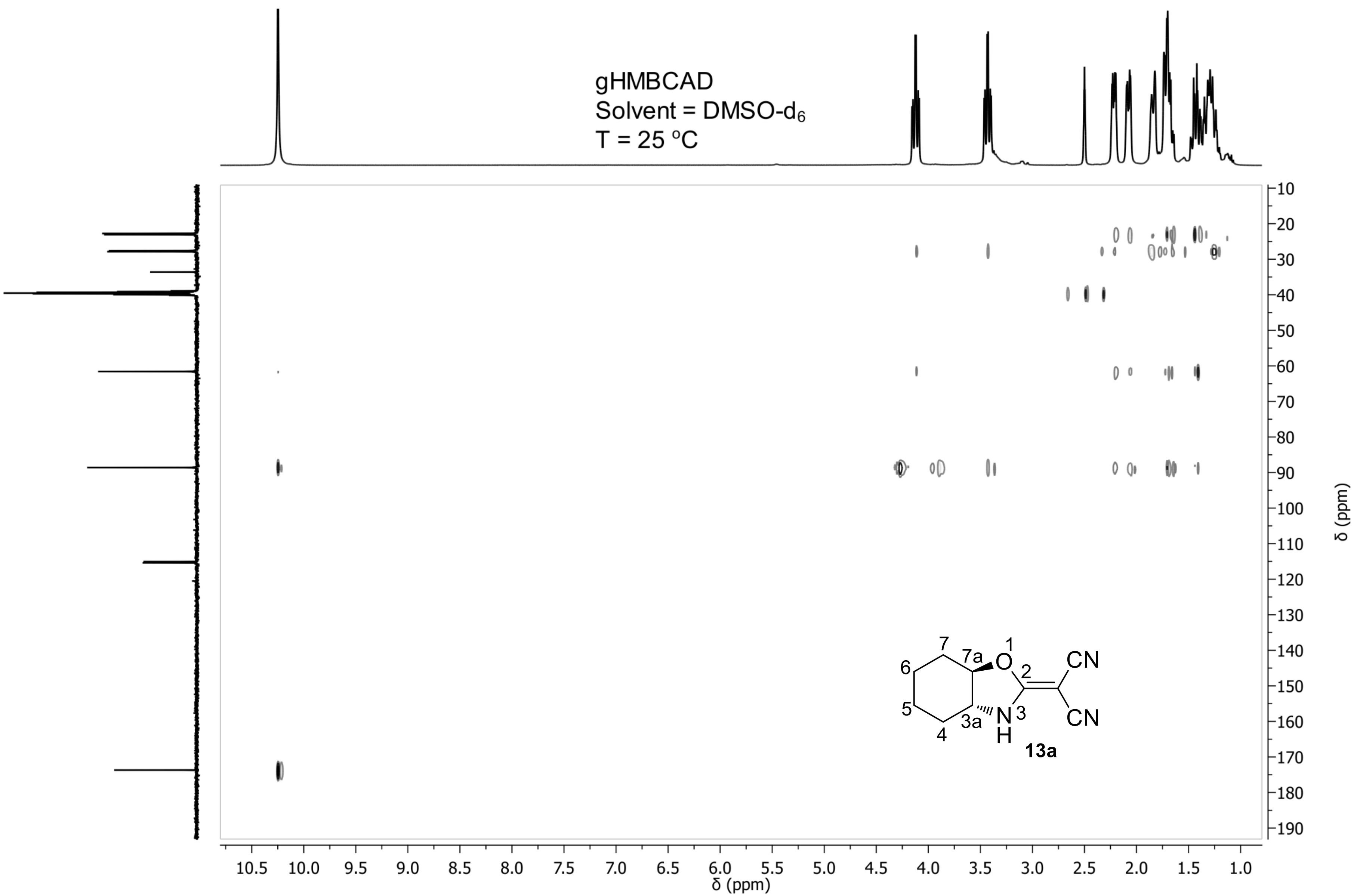
gHMBCAD
Solvent = DMSO-d6
T = 25 °C
13a
7
7a
O
1
6
2
CN
5
3a
N
3
CN
4
H
δ (ppm)
δ (ppm)
10.5 10.0 9.5 9.0 8.5 8.0 7.5 7.0 6.5 6.0 5.5 5.0 4.5 4.0 3.5 3.0 2.5 2.0 1.5 1.0
10 20 30 40 50 60 70 80 90 100 110 120 130 140 150 160 170 180 190

^{1}H NMR
Solvent = CD_3CN
T = 25 °C

13b

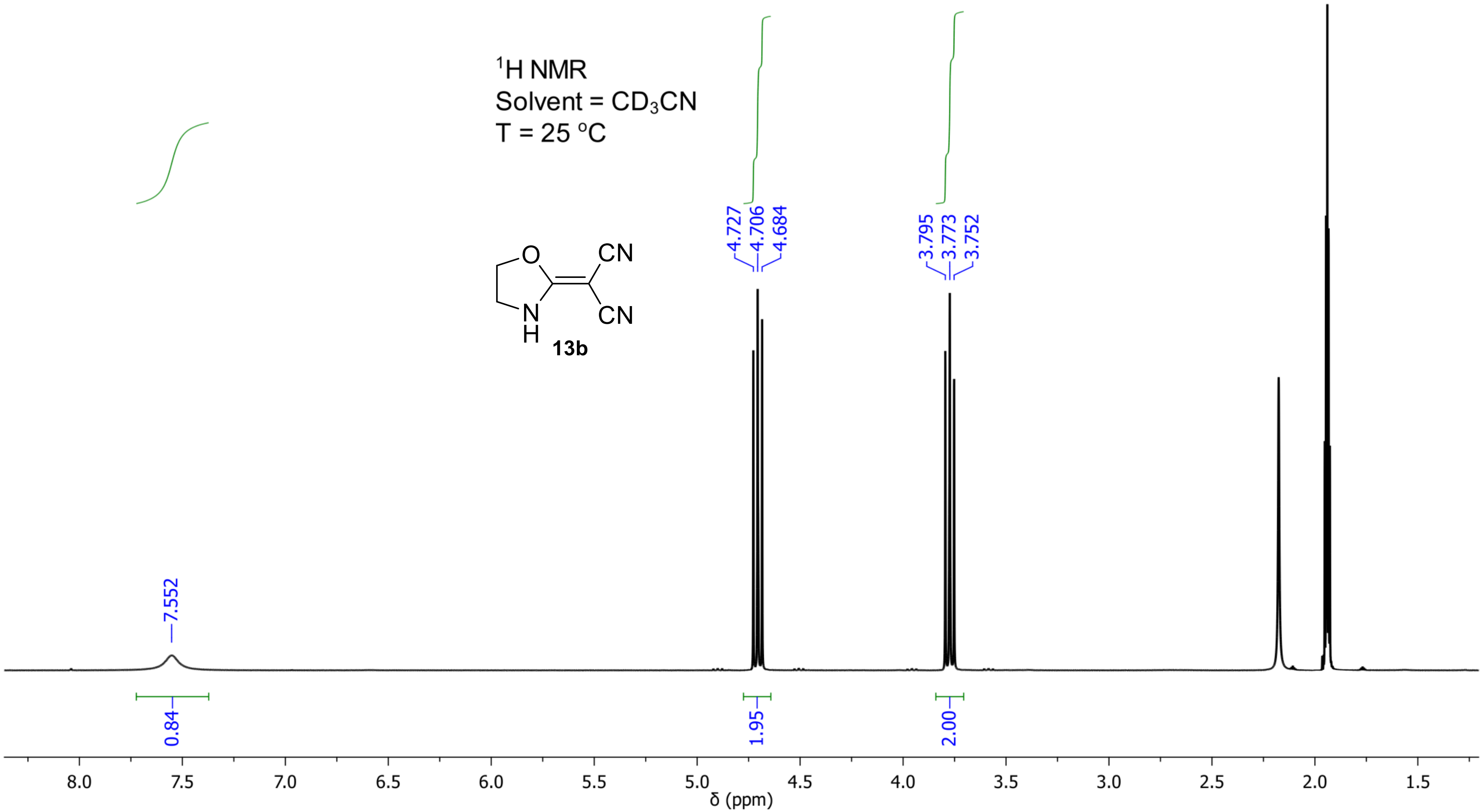

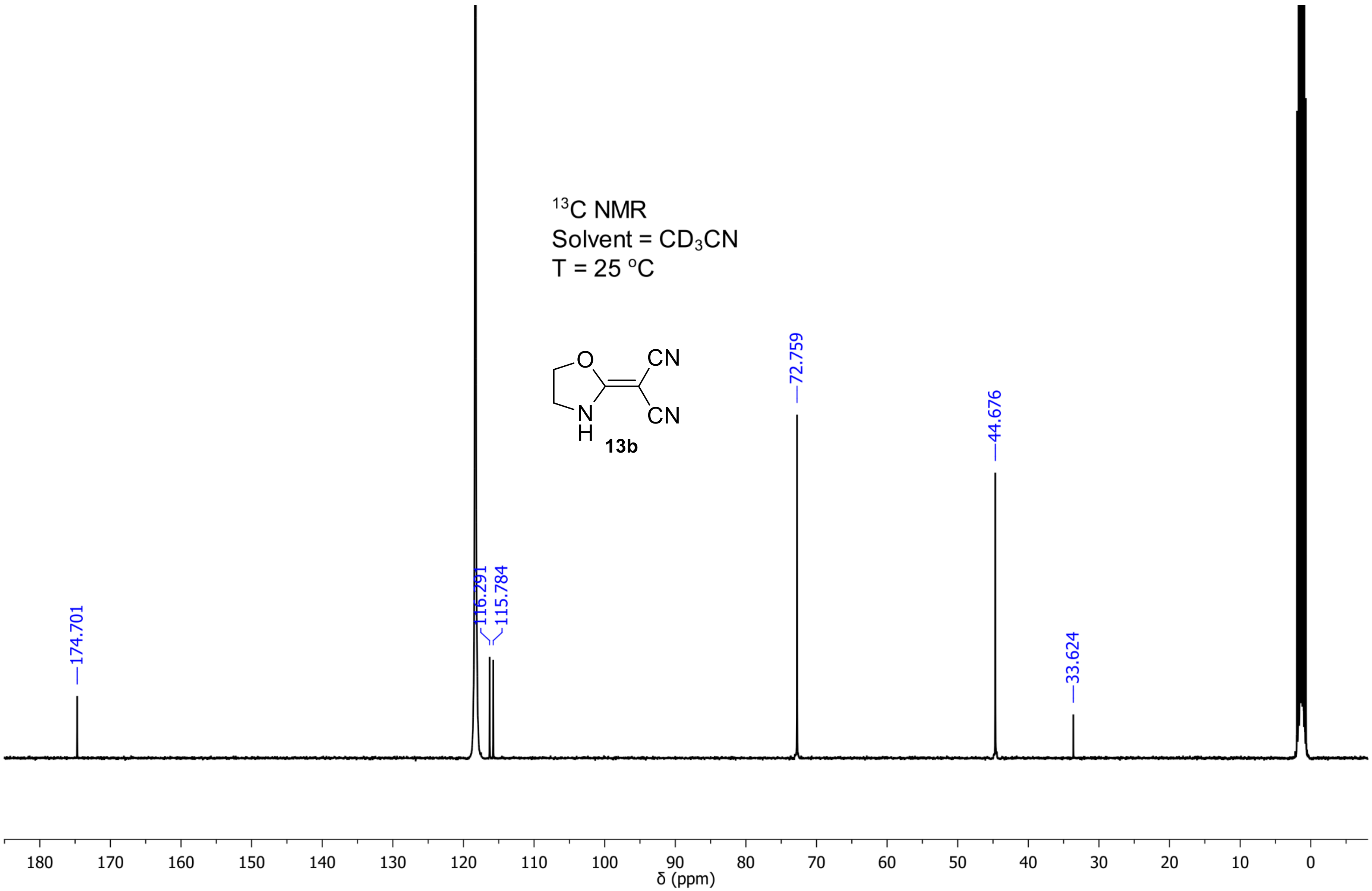

^{13}C NMR
Solvent = CD_3CN
T = 25 °C
O
N
H
CN
CN
13b
174.701
116.291
115.784
72.759
44.676
33.624
180
170
160
150
140
130
120
110
100
90
80
70
60
50
40
30
20
10
0
δ (ppm)

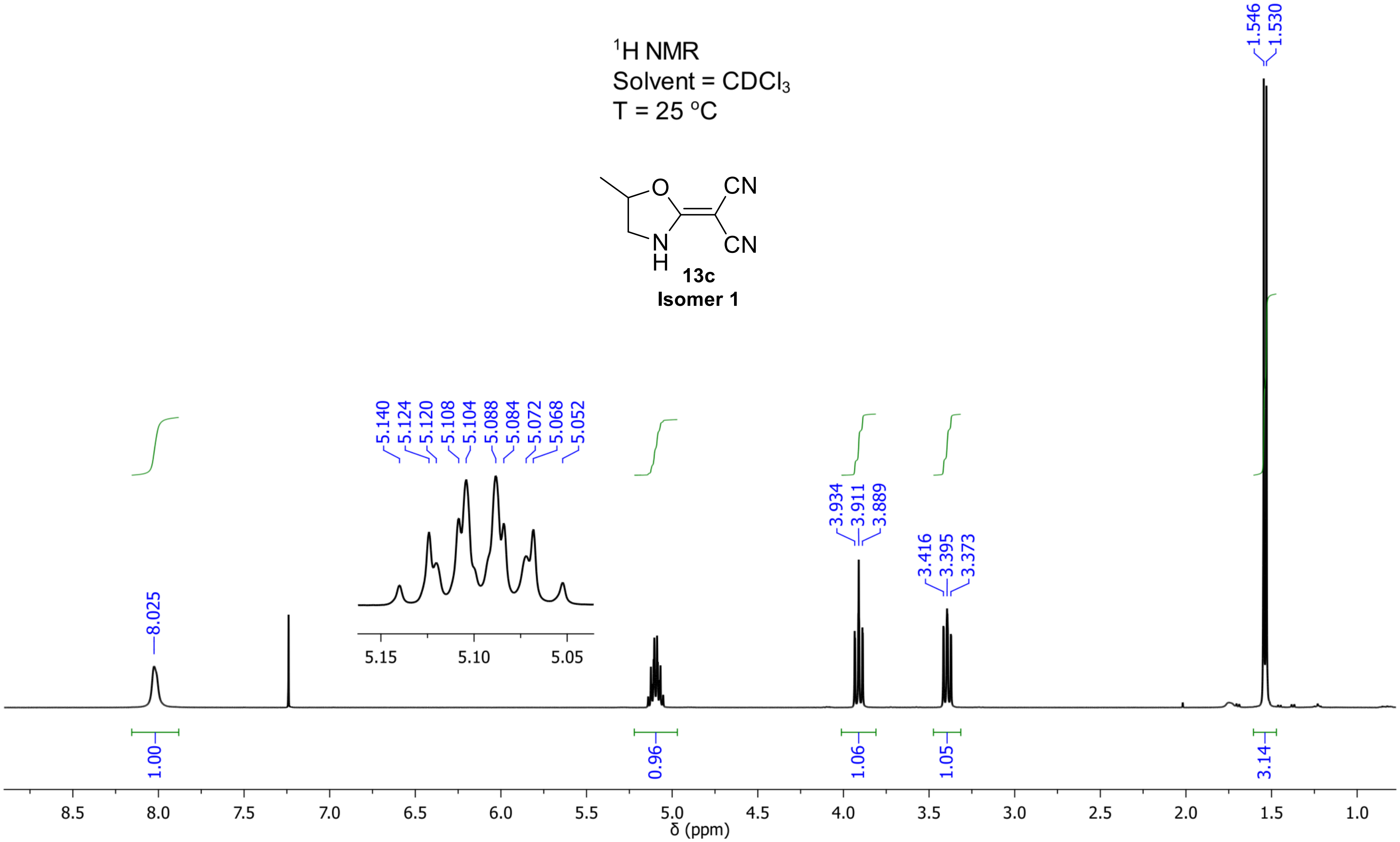

1H NMR
Solvent = CDCl3
T = 25 °C
O
CN
N
H
CN
13c
Isomer 1
8.025
5.140
5.124
5.120
5.108
5.104
5.088
5.084
5.072
5.068
5.052
3.934
3.911
3.889
3.416
3.395
3.373
1.546
1.530
1.00
0.96
1.06
1.05
3.14
5.15
5.10
5.05
8.5
8.0
7.5
7.0
6.5
6.0
5.5
5.0
4.5
4.0
3.5
3.0
2.5
2.0
1.5
1.0
δ (ppm)

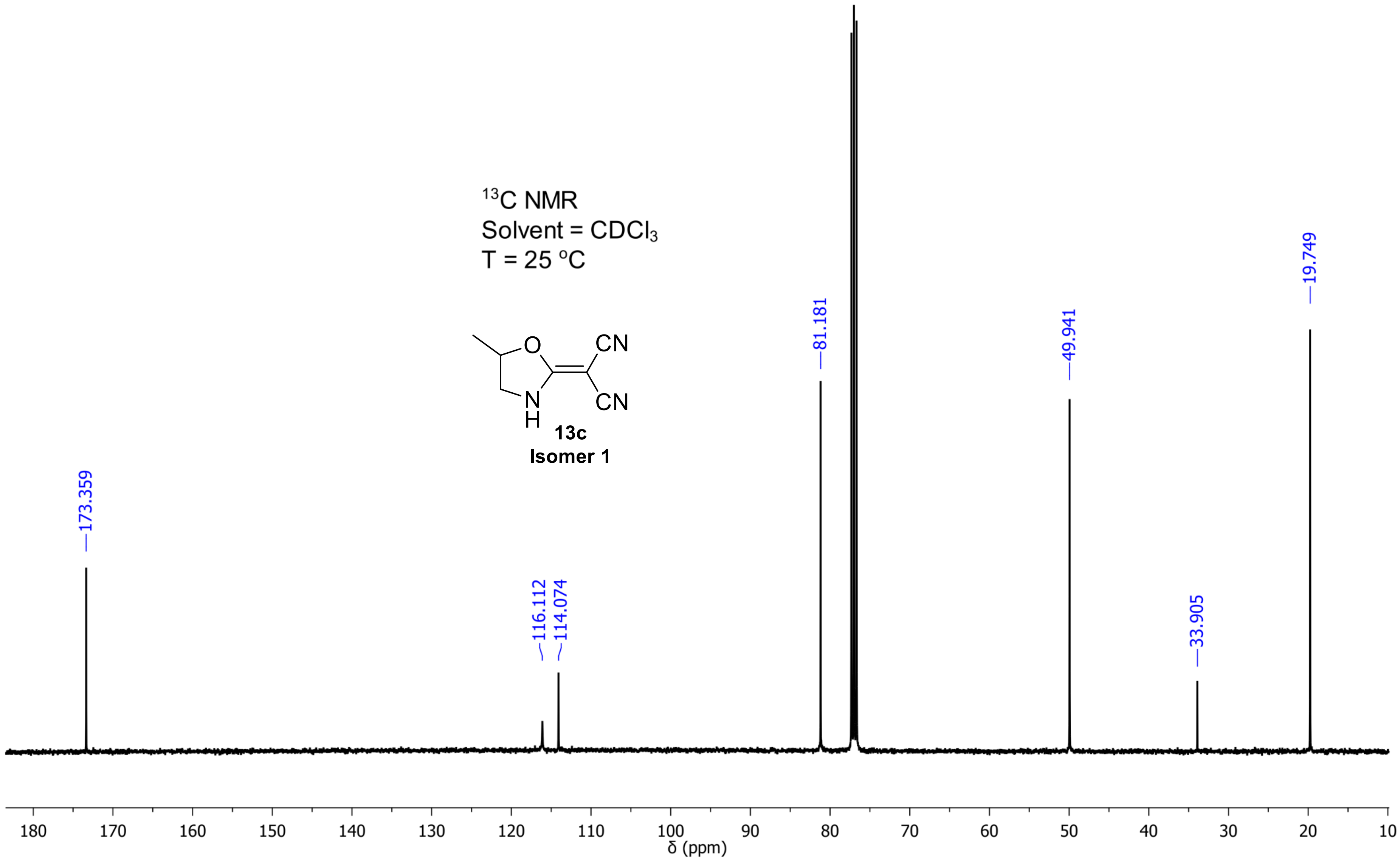
^{13}C NMR
Solvent = $CDCl_3$
T = 25 °C
O
CN
N
H
CN
13c
Isomer 1
173.359
116.112
114.074
81.181
49.941
33.905
19.749
180 170 160 150 140 130 120 110 100 90 80 70 60 50 40 30 20 10
δ (ppm)

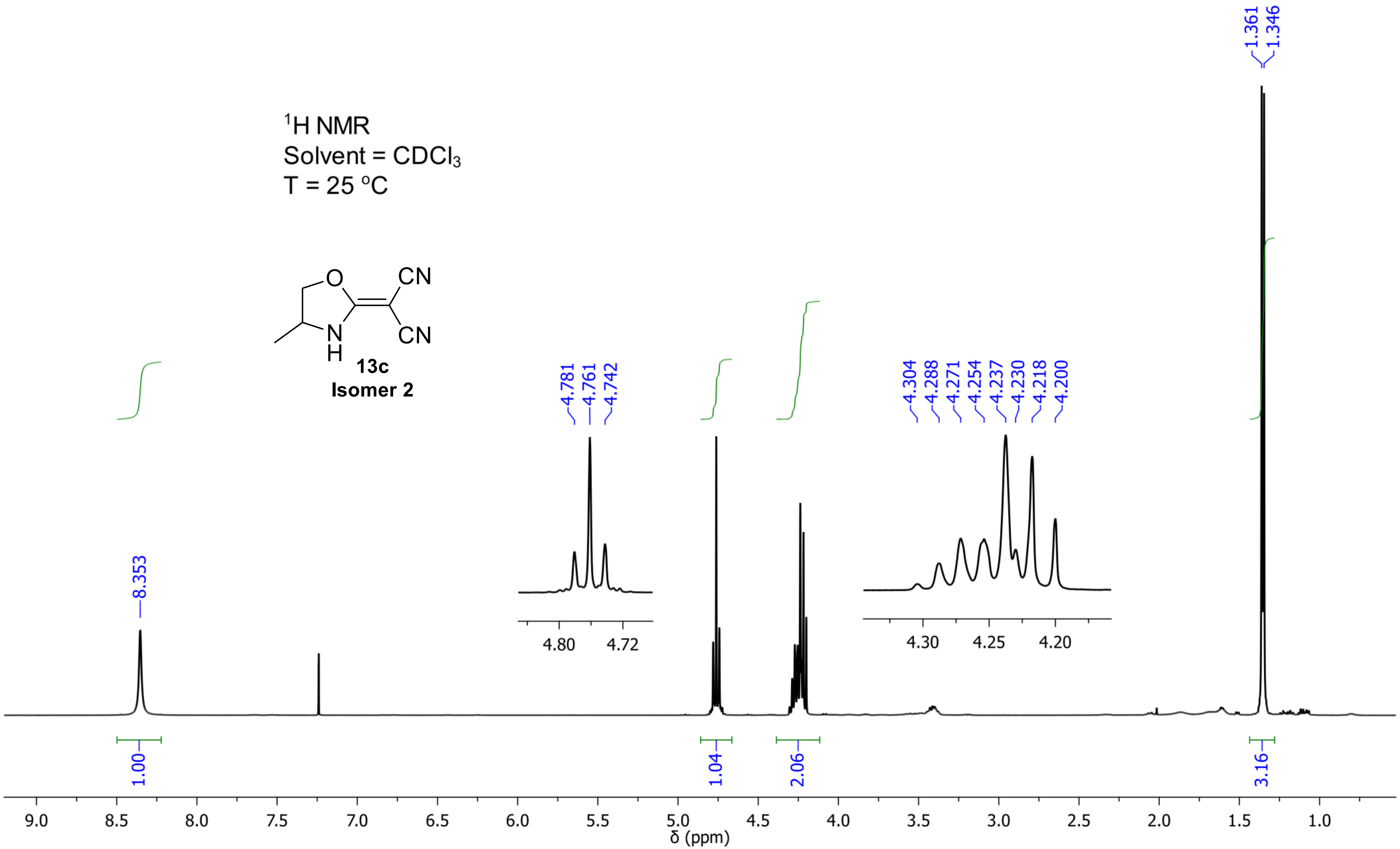

1H NMR
Solvent = CDCl3
T = 25 °C
13c
Isomer 2
8.353
4.781
4.761
4.742
4.304
4.288
4.271
4.254
4.237
4.230
4.218
4.200
1.361
1.346
1.00
1.04
2.06
3.16
4.80
4.72
4.30
4.25
4.20
9.0
8.5
8.0
7.5
7.0
6.5
6.0
5.5
5.0
4.5
4.0
3.5
3.0
2.5
2.0
1.5
1.0
δ (ppm)

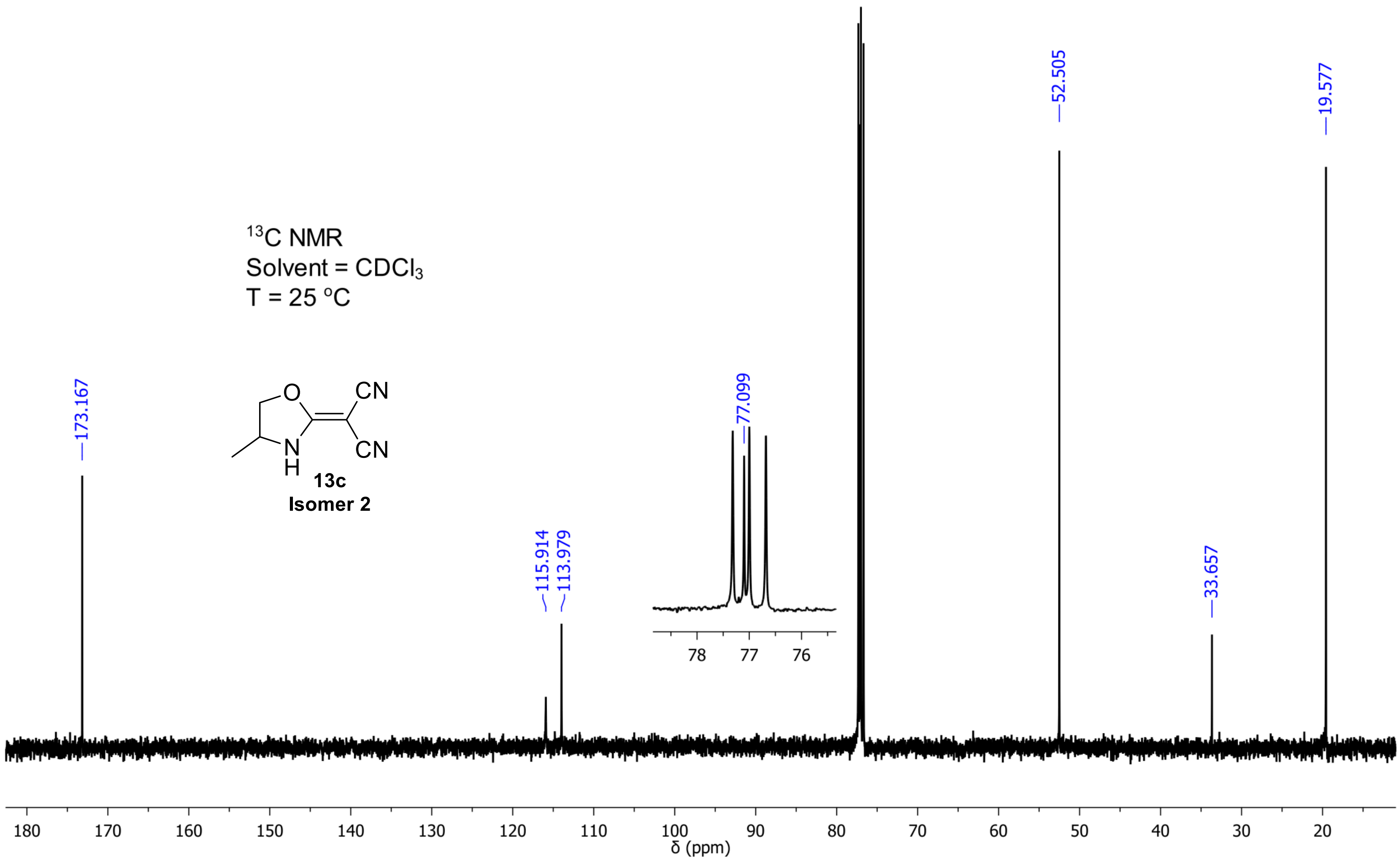

^{13}C NMR
Solvent = $CDCl_3$
T = 25 °C
O
CN
N
H
CN
13c
Isomer 2
173.167
115.914
113.979
77.099
52.505
33.657
19.577
78
77
76
180
170
160
150
140
130
120
110
100
90
80
70
60
50
40
30
20
δ (ppm)

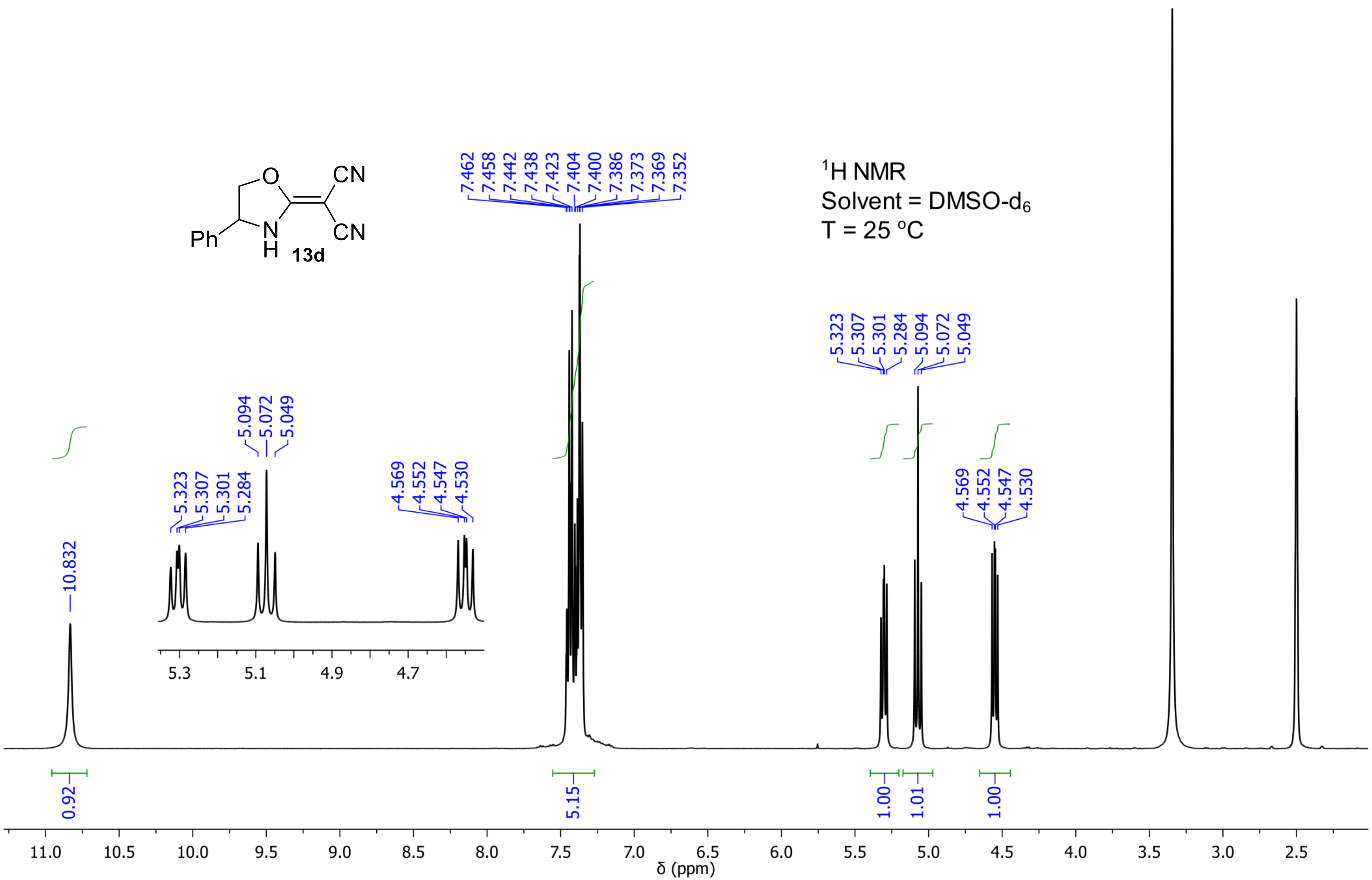
O
CN
Ph
N
H
CN
13d
7.462
7.458
7.442
7.438
7.423
7.404
7.400
7.386
7.373
7.369
7.352
1H NMR
Solvent = DMSO-d6
T = 25 °C
10.832
5.323
5.307
5.301
5.284
5.094
5.072
5.049
4.569
4.552
4.547
4.530
0.92
5.15
1.00
1.01
1.00
δ (ppm)

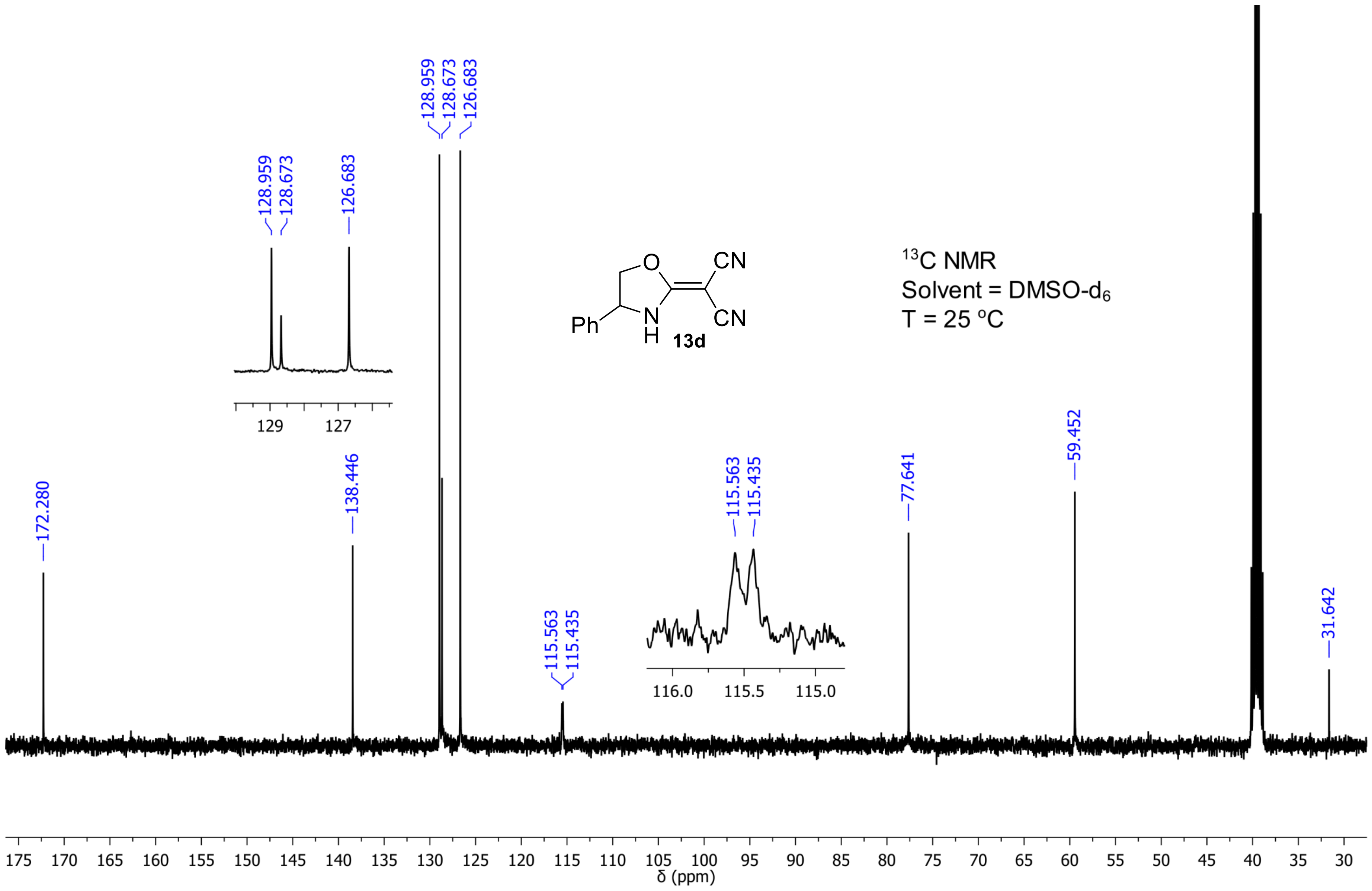

172.280
138.446
128.959
128.673
126.683
129
127
115.563
115.435
116.0
115.5
115.0
77.641
59.452
31.642
O
CN
Ph
N
H
CN
13d
13C NMR
Solvent = DMSO-d6
T = 25 °C
175 170 165 160 155 150 145 140 135 130 125 120 115 110 105 100 95 90 85 80 75 70 65 60 55 50 45 40 35 30
δ (ppm)

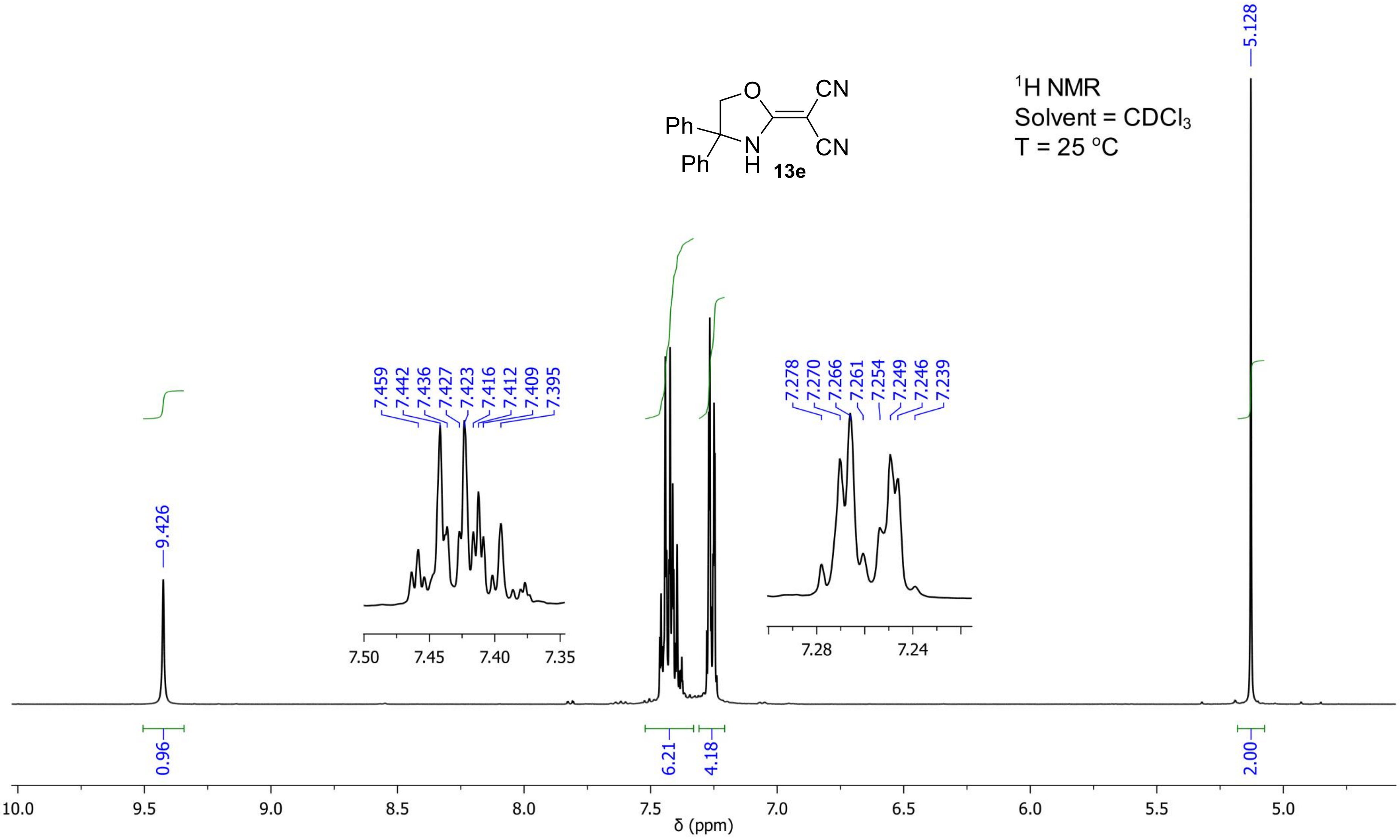
Ph
Ph
O
N
H
CN
CN
13e
^{1}H NMR
Solvent = $CDCl_3$
T = 25 °C
5.128
9.426
7.459
7.442
7.436
7.427
7.423
7.416
7.412
7.409
7.395
7.50
7.45
7.40
7.35
7.278
7.270
7.266
7.261
7.254
7.249
7.246
7.239
7.28
7.24
0.96
6.21
4.18
2.00
10.0
9.5
9.0
8.5
8.0
7.5
7.0
6.5
6.0
5.5
5.0
δ (ppm)

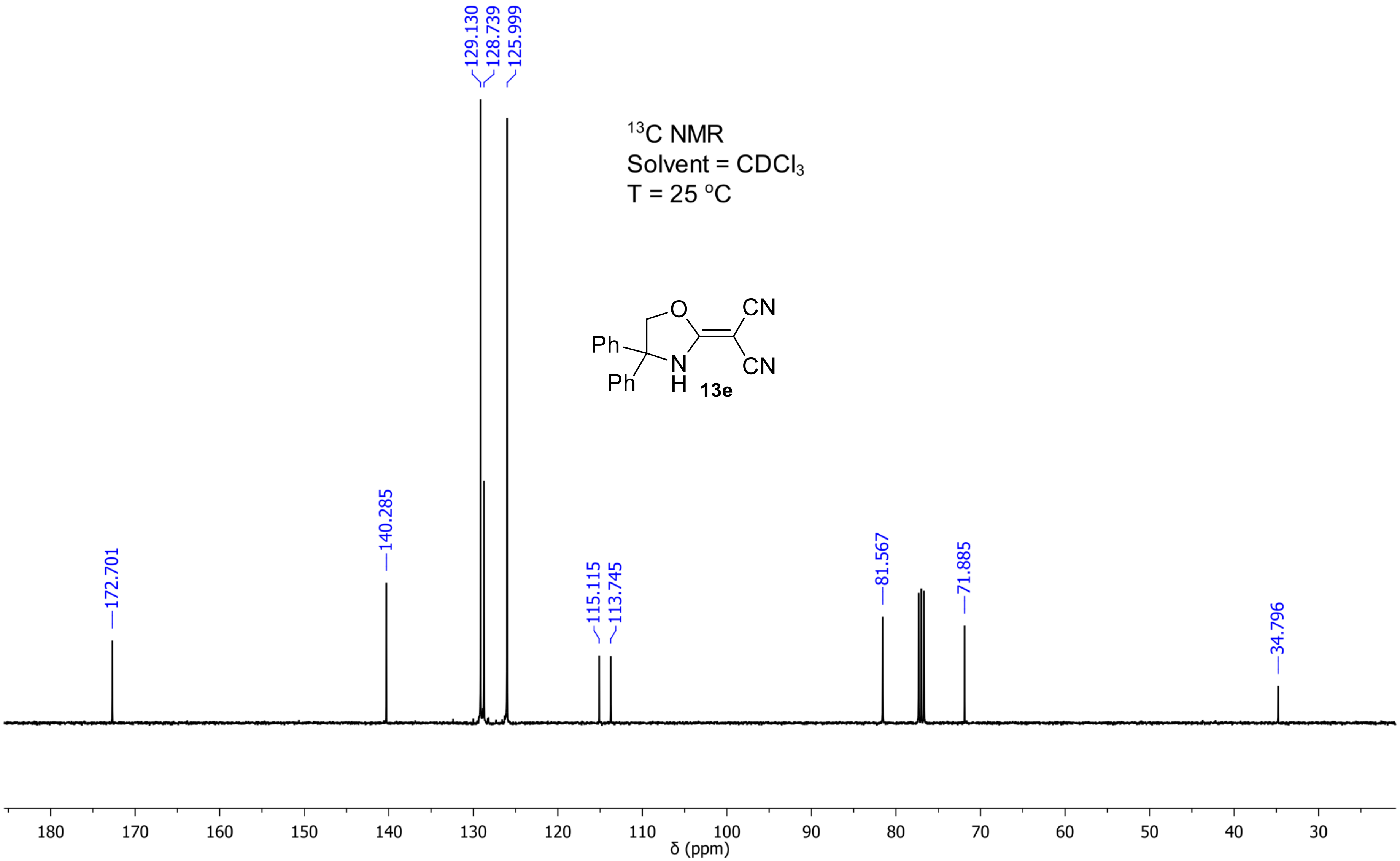

129.130
128.739
125.999
13C NMR
Solvent = CDCl3
T = 25 °C
O
CN
Ph
N
CN
Ph
H
13e
172.701
140.285
115.115
113.745
81.567
71.885
34.796
180
170
160
150
140
130
120
110
100
90
80
70
60
50
40
30
δ (ppm)

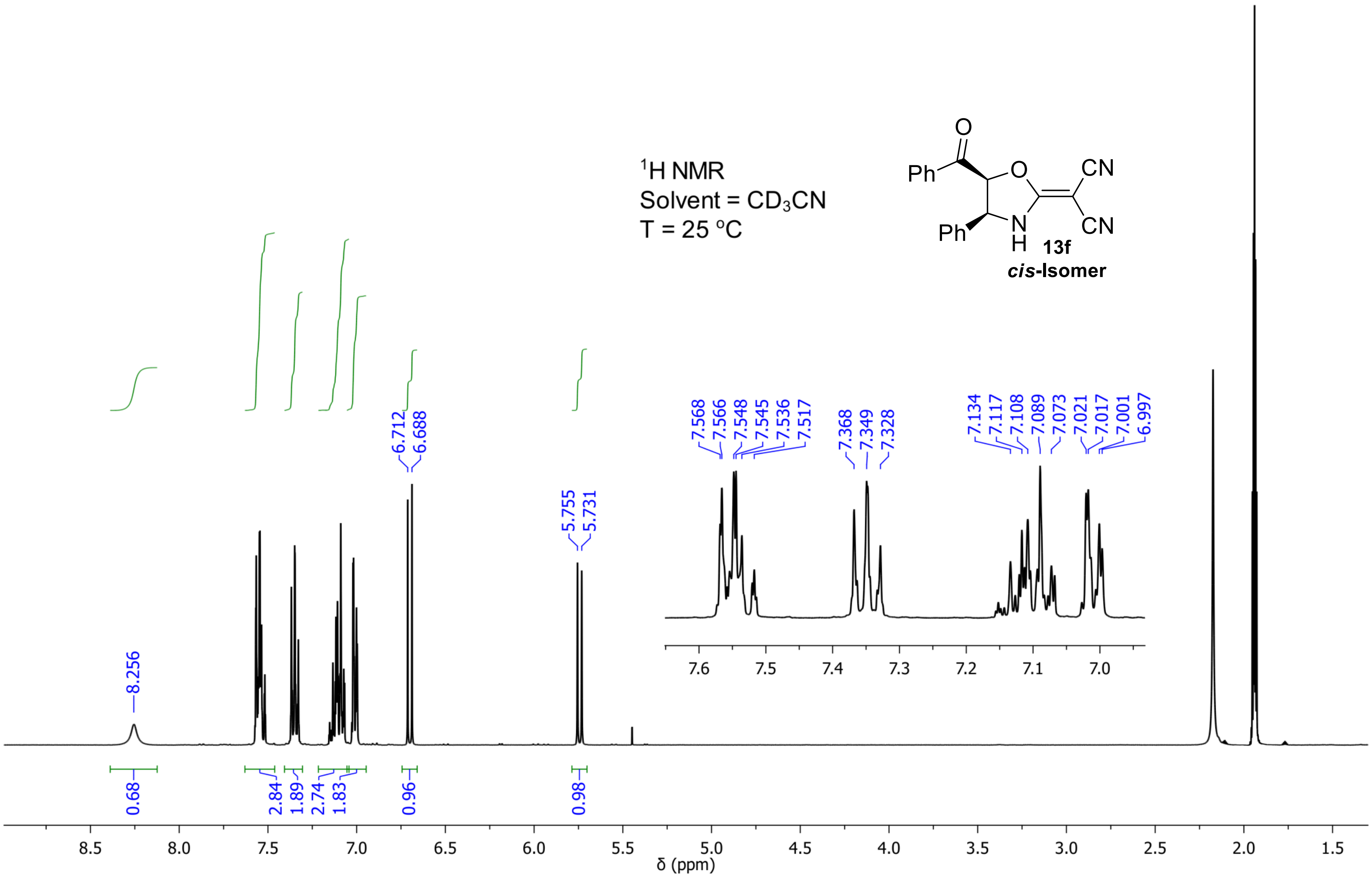

^{1}H NMR
Solvent = CD3CN
T = 25 °C
13f
cis-Isomer
δ (ppm)

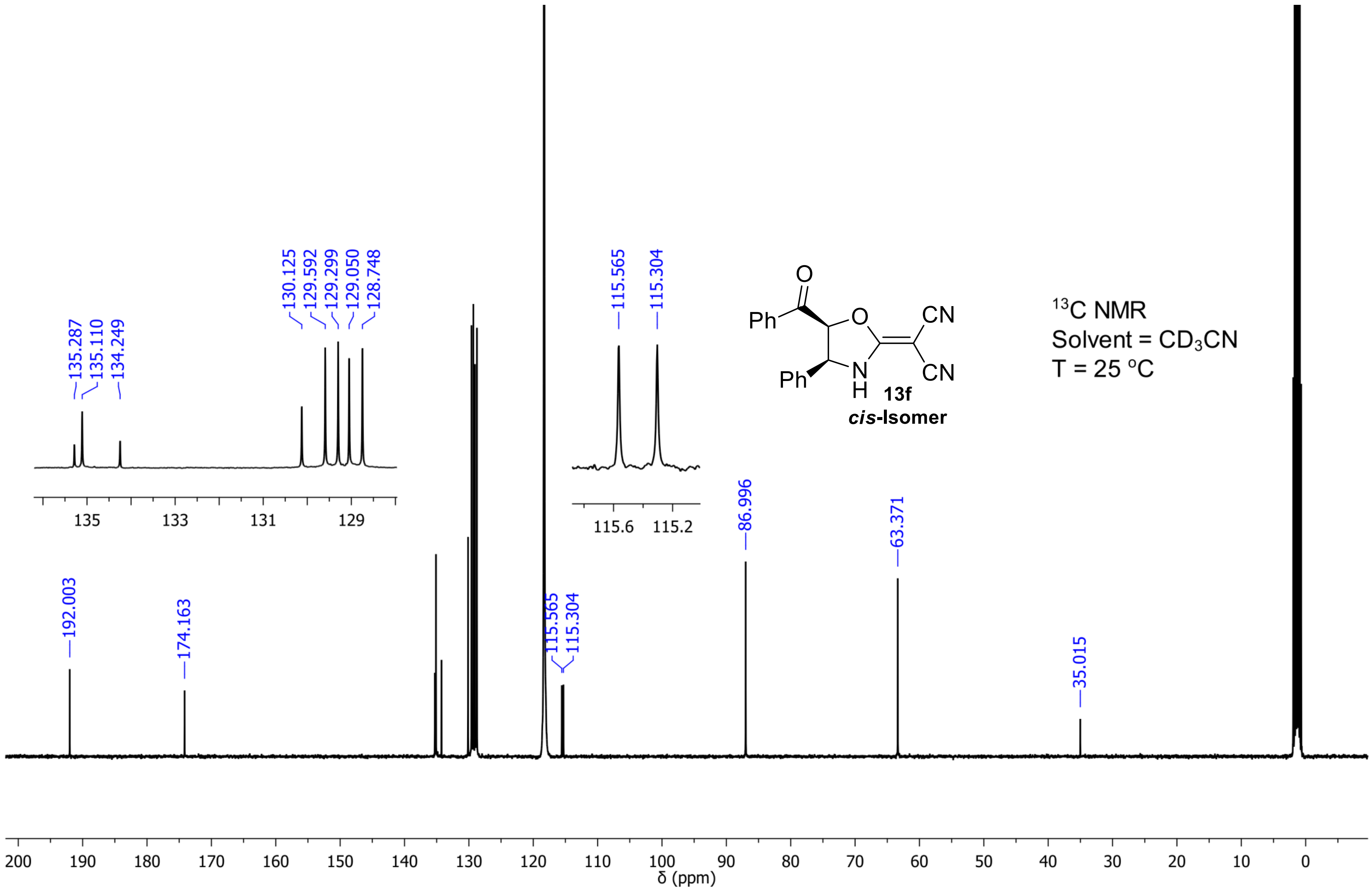

135.287
135.110
134.249
130.125
129.592
129.299
129.050
128.748
135
133
131
129
115.565
115.304
115.6
115.2
O
Ph
O
CN
CN
Ph
N
H
13f
cis-Isomer
13C NMR
Solvent = CD3CN
T = 25 °C
192.003
174.163
115.565
115.304
86.996
63.371
35.015
200
190
180
170
160
150
140
130
120
110
100
90
80
70
60
50
40
30
20
10
0
δ (ppm)

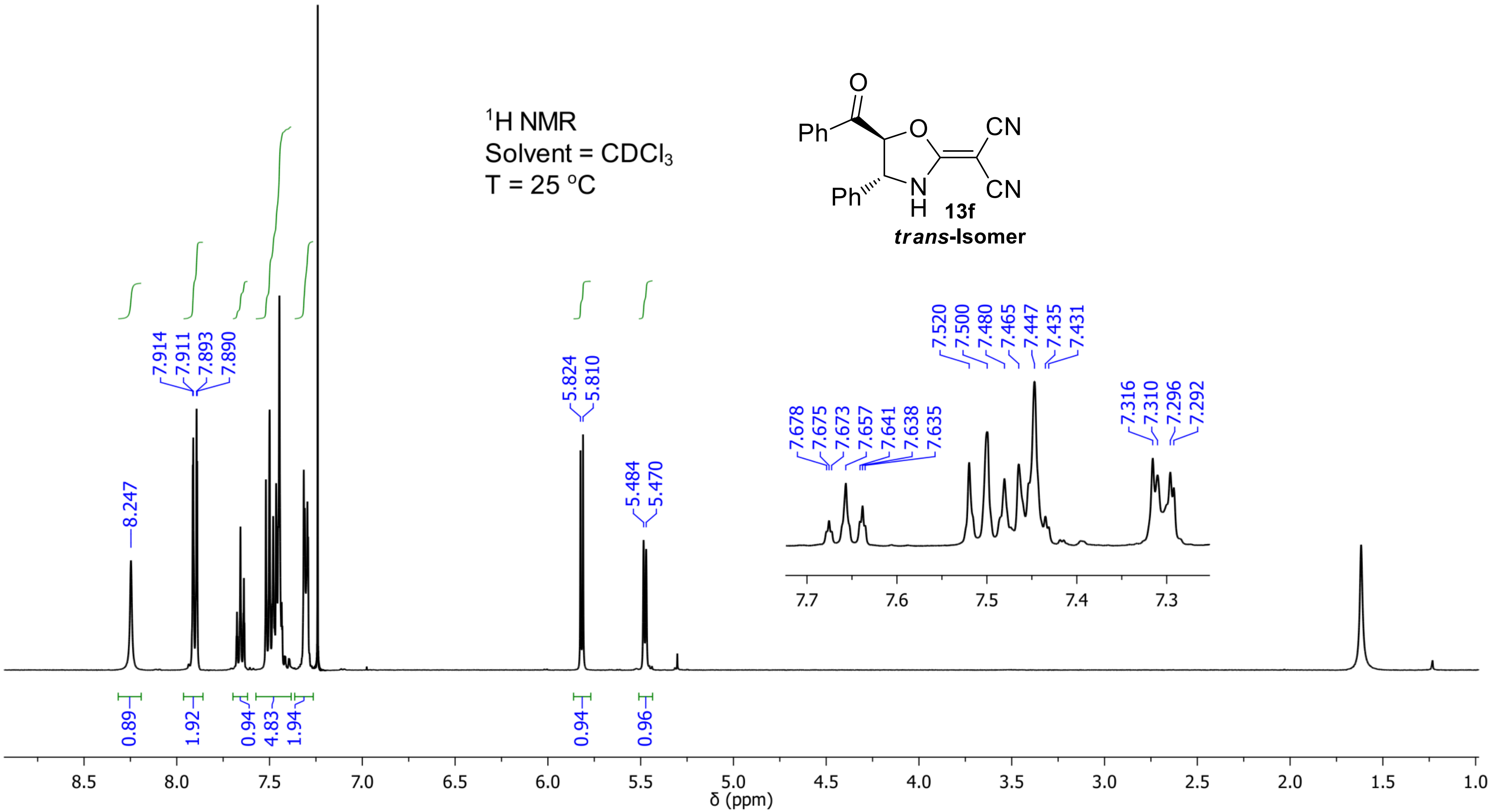

1H NMR
Solvent = CDCl3
T = 25 °C
13f
trans-Isomer
Ph
O
CN
N
H
8.247
7.914
7.911
7.893
7.890
5.824
5.810
5.484
5.470
7.678
7.675
7.673
7.657
7.641
7.638
7.635
7.520
7.500
7.480
7.465
7.447
7.435
7.431
7.316
7.310
7.296
7.292
0.89
1.92
0.94
4.83
1.94
0.94
0.96
δ (ppm)

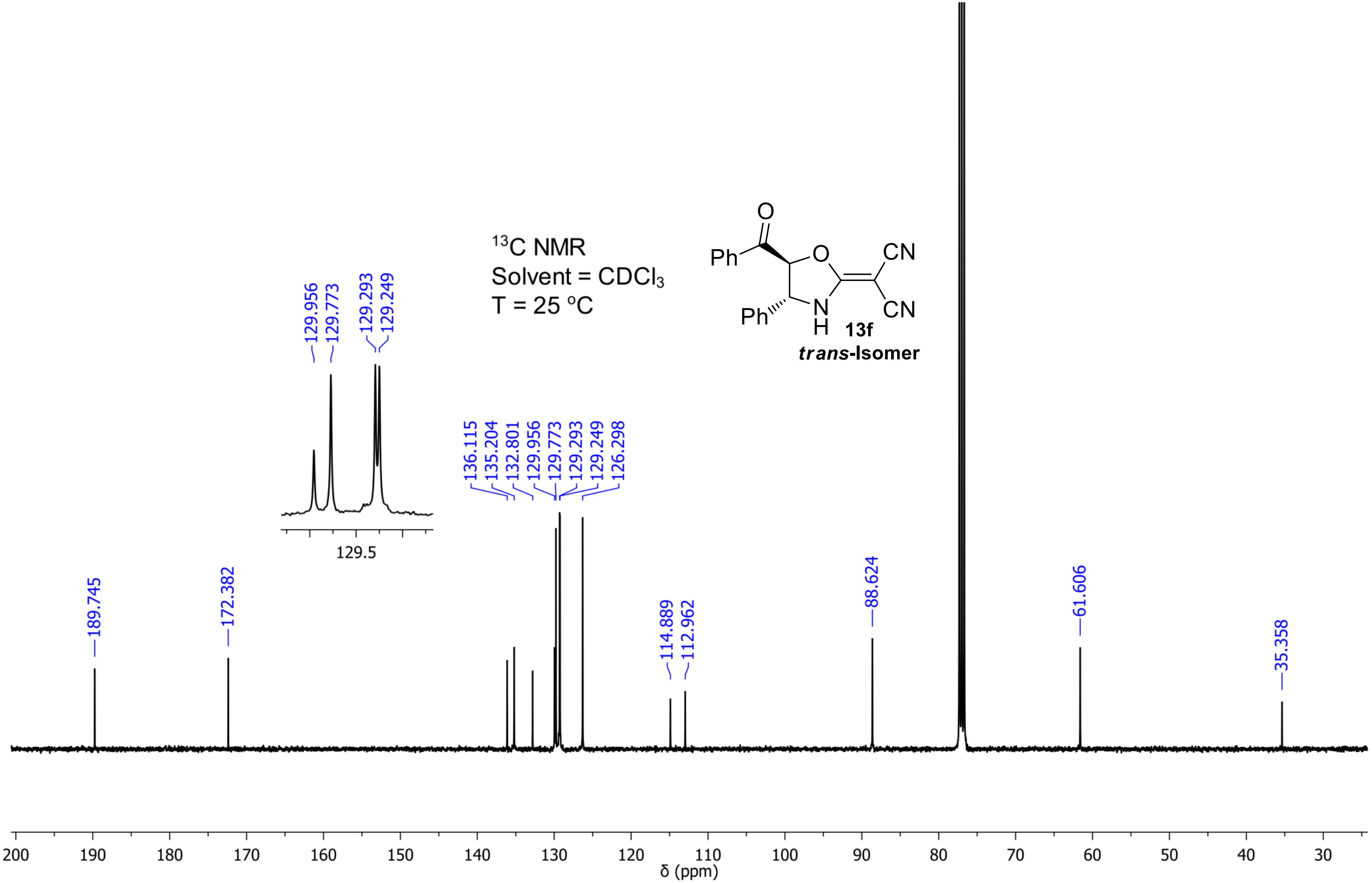

13C NMR
Solvent = CDCl3
T = 25 oC
13f
trans-Isomer
Ph
O
CN
N
H
129.956
129.773
129.293
129.249
129.5
136.115
135.204
132.801
126.298
189.745
172.382
114.889
112.962
88.624
61.606
35.358
δ (ppm)

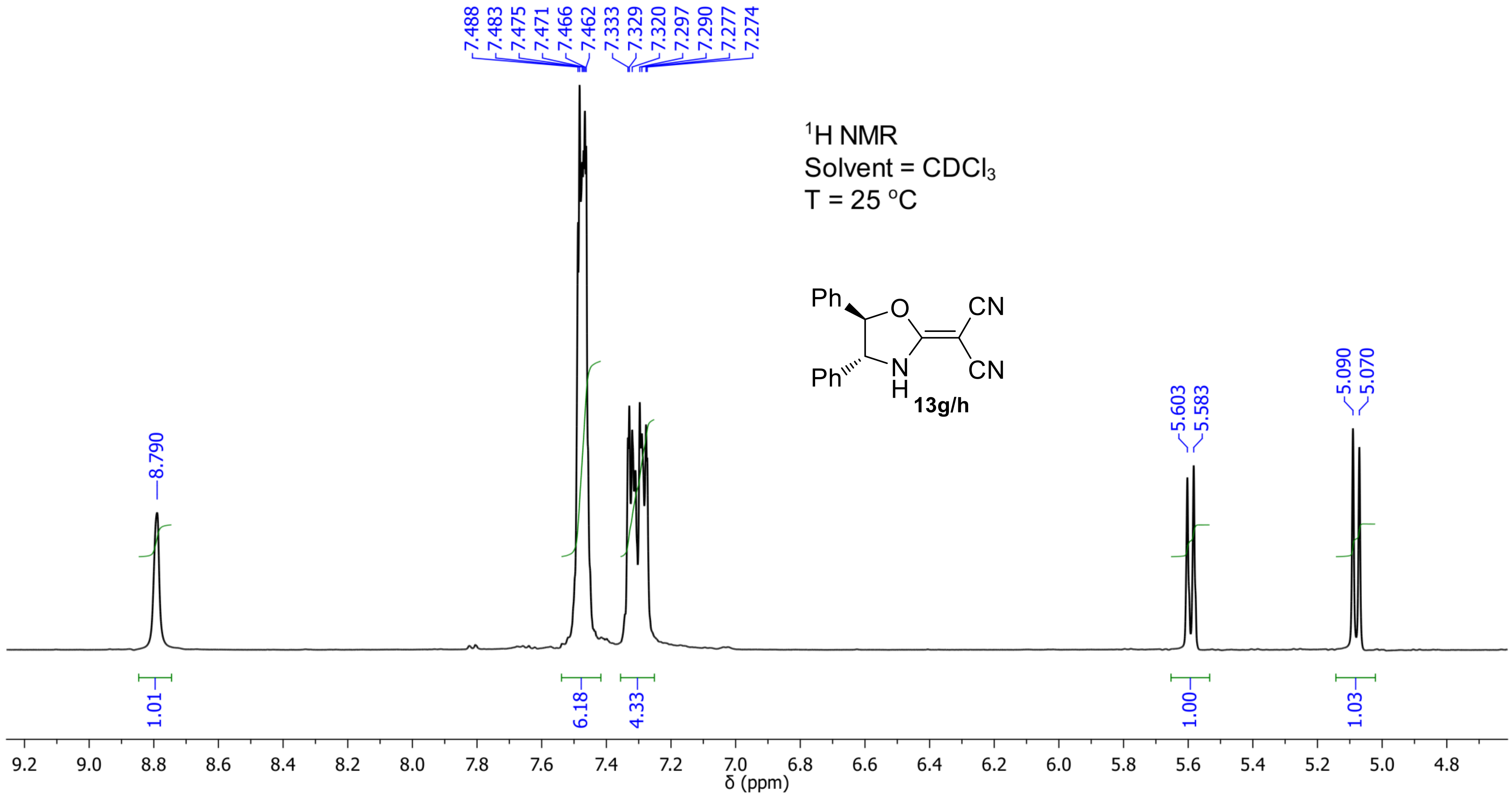

7.488
7.483
7.475
7.471
7.466
7.462
7.333
7.329
7.320
7.297
7.290
7.277
7.274
^{1}H NMR
Solvent = $CDCl_3$
T = 25 °C
Ph
O
CN
CN
Ph
N
H
13g/h
8.790
5.603
5.583
5.090
5.070
1.01
6.18
4.33
1.00
1.03
9.2
9.0
8.8
8.6
8.4
8.2
8.0
7.8
7.6
7.4
7.2
7.0
6.8
6.6
6.4
6.2
6.0
5.8
5.6
5.4
5.2
5.0
4.8
δ (ppm)

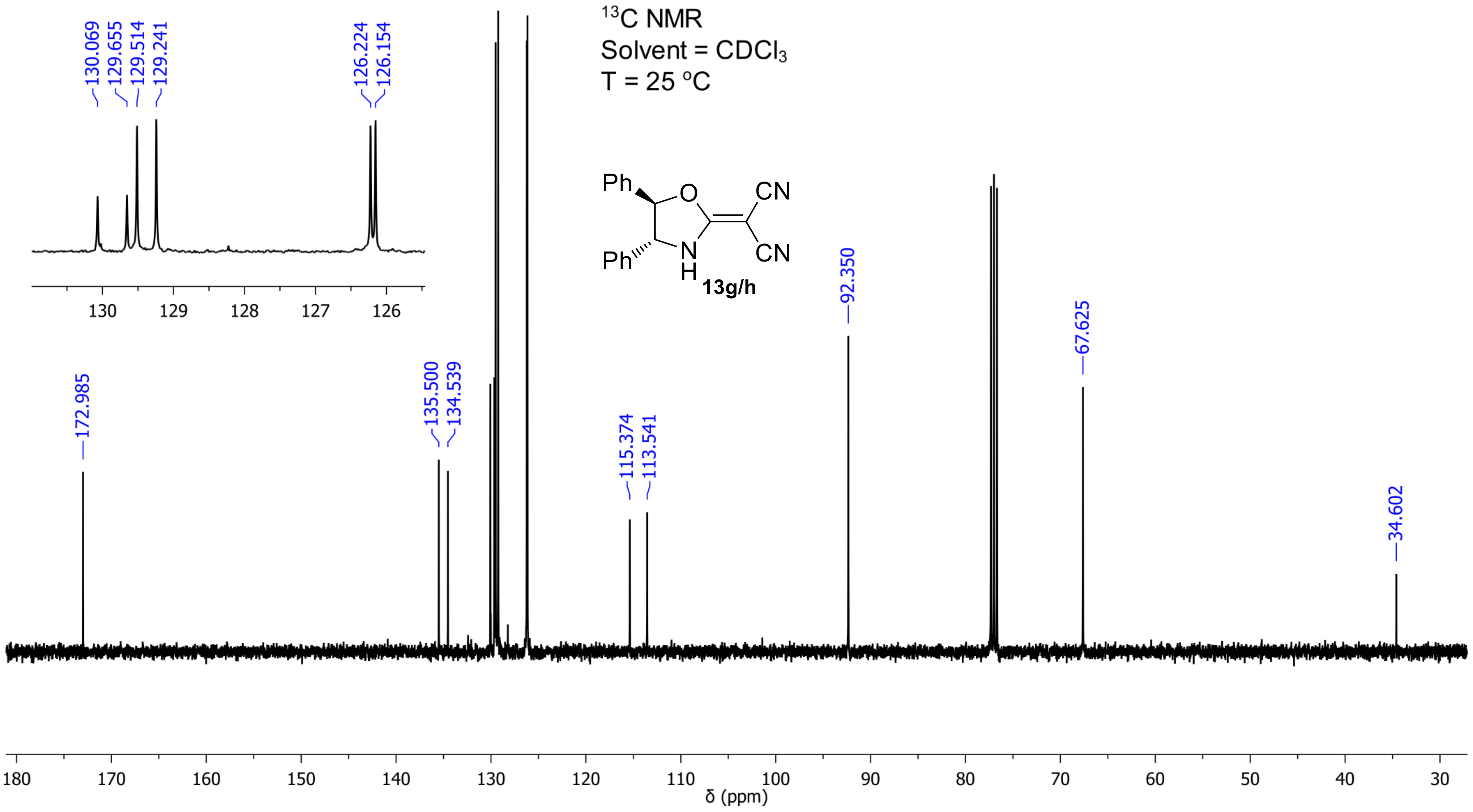

130.069
129.655
129.514
129.241
126.224
126.154
130
129
128
127
126
^{13}C NMR
Solvent = CDCl$_3$
T = 25 °C
Ph
O
CN
Ph
N
H
CN
13g/h
172.985
135.500
134.539
115.374
113.541
92.350
67.625
34.602
180
170
160
150
140
130
120
110
100
90
80
70
60
50
40
30
δ (ppm)

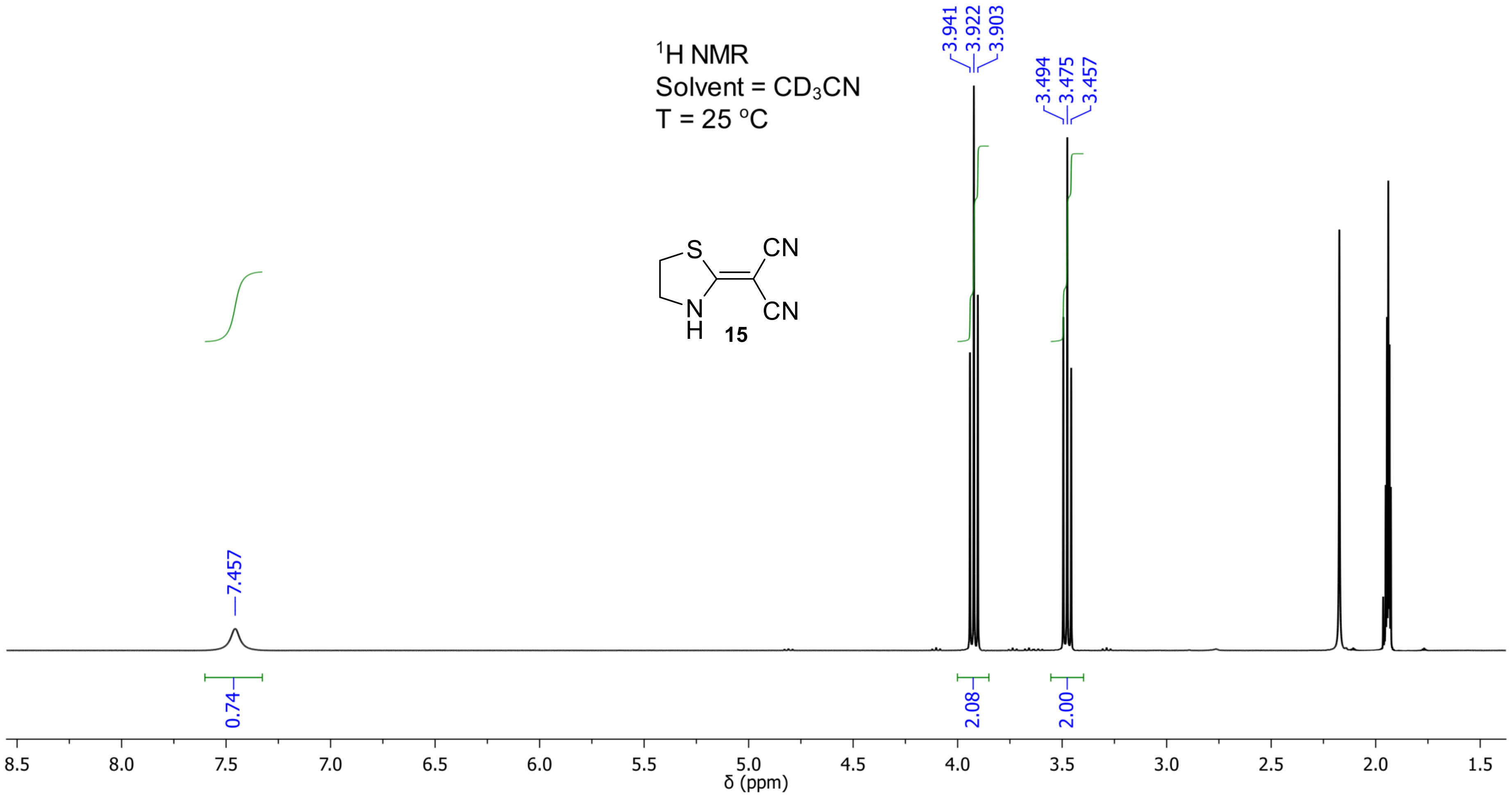

^{1}H NMR
Solvent = CD_3CN
T = 25 °C
S
N
H
CN
CN
15
3.941
3.922
3.903
3.494
3.475
3.457
7.457
0.74
2.08
2.00
8.5
8.0
7.5
7.0
6.5
6.0
5.5
5.0
4.5
4.0
3.5
3.0
2.5
2.0
1.5
δ (ppm)

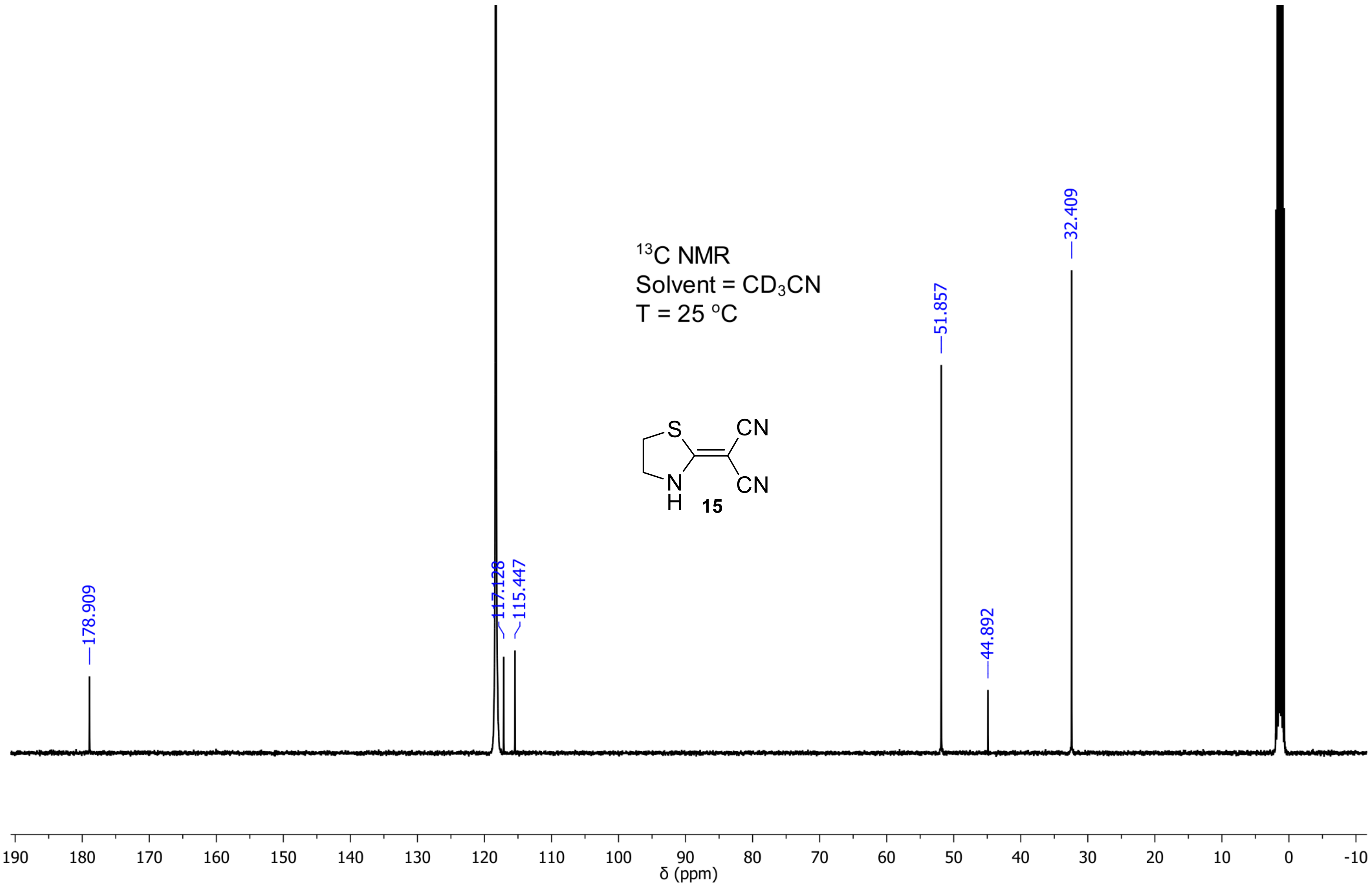

13C NMR
Solvent = CD3CN
T = 25 °C
S
CN
N
H
CN
15
178.909
117.128
115.447
51.857
44.892
32.409
190
180
170
160
150
140
130
120
110
100
90
80
70
60
50
40
30
20
10
0
-10
δ (ppm)

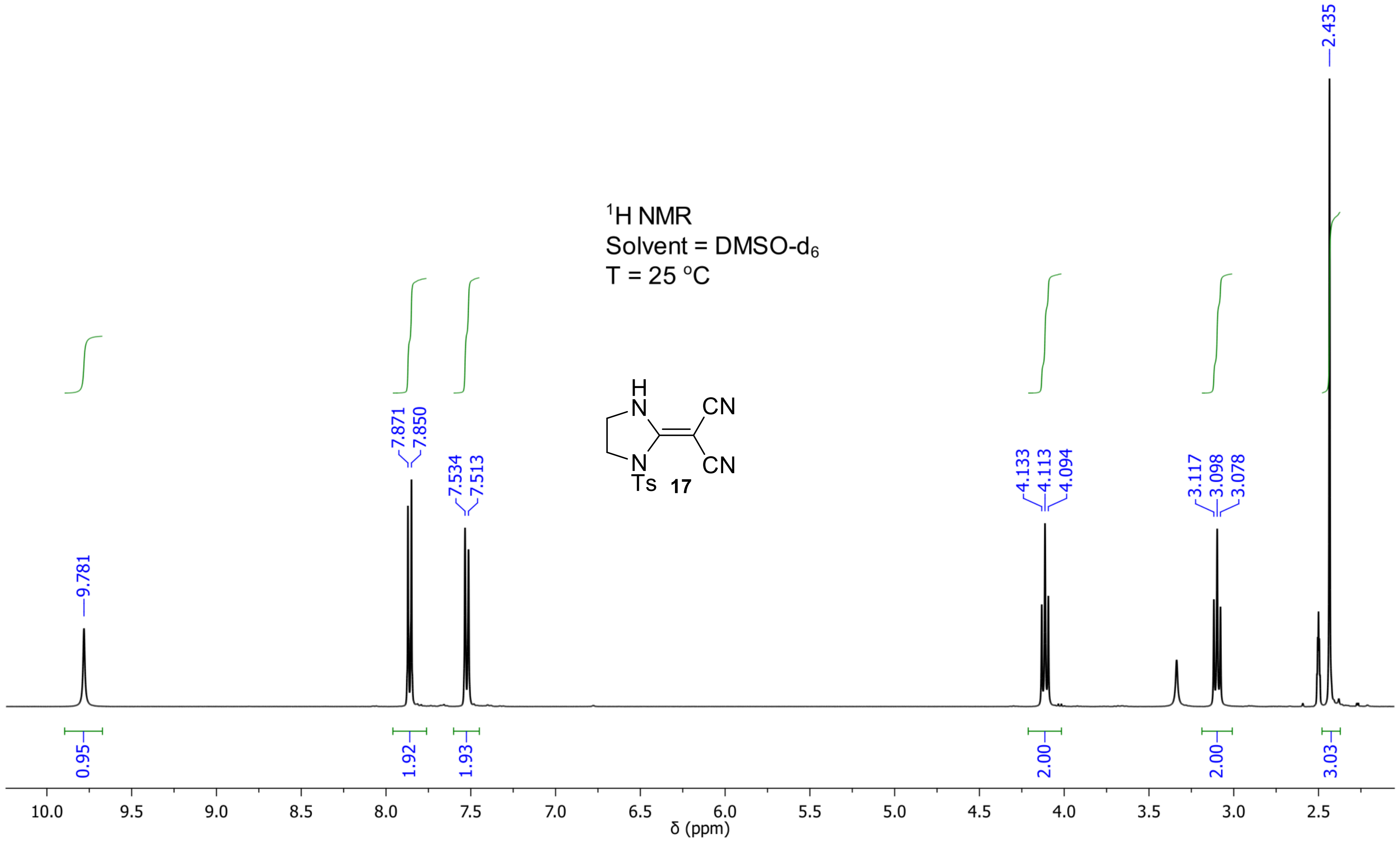

1H NMR
Solvent = DMSO-d6
T = 25 °C
H
N
CN
N
CN
Ts
17
9.781
7.871
7.850
7.534
7.513
4.133
4.113
4.094
3.117
3.098
3.078
2.435
0.95
1.92
1.93
2.00
2.00
3.03
10.0
9.5
9.0
8.5
8.0
7.5
7.0
6.5
6.0
5.5
5.0
4.5
4.0
3.5
3.0
2.5
δ (ppm)

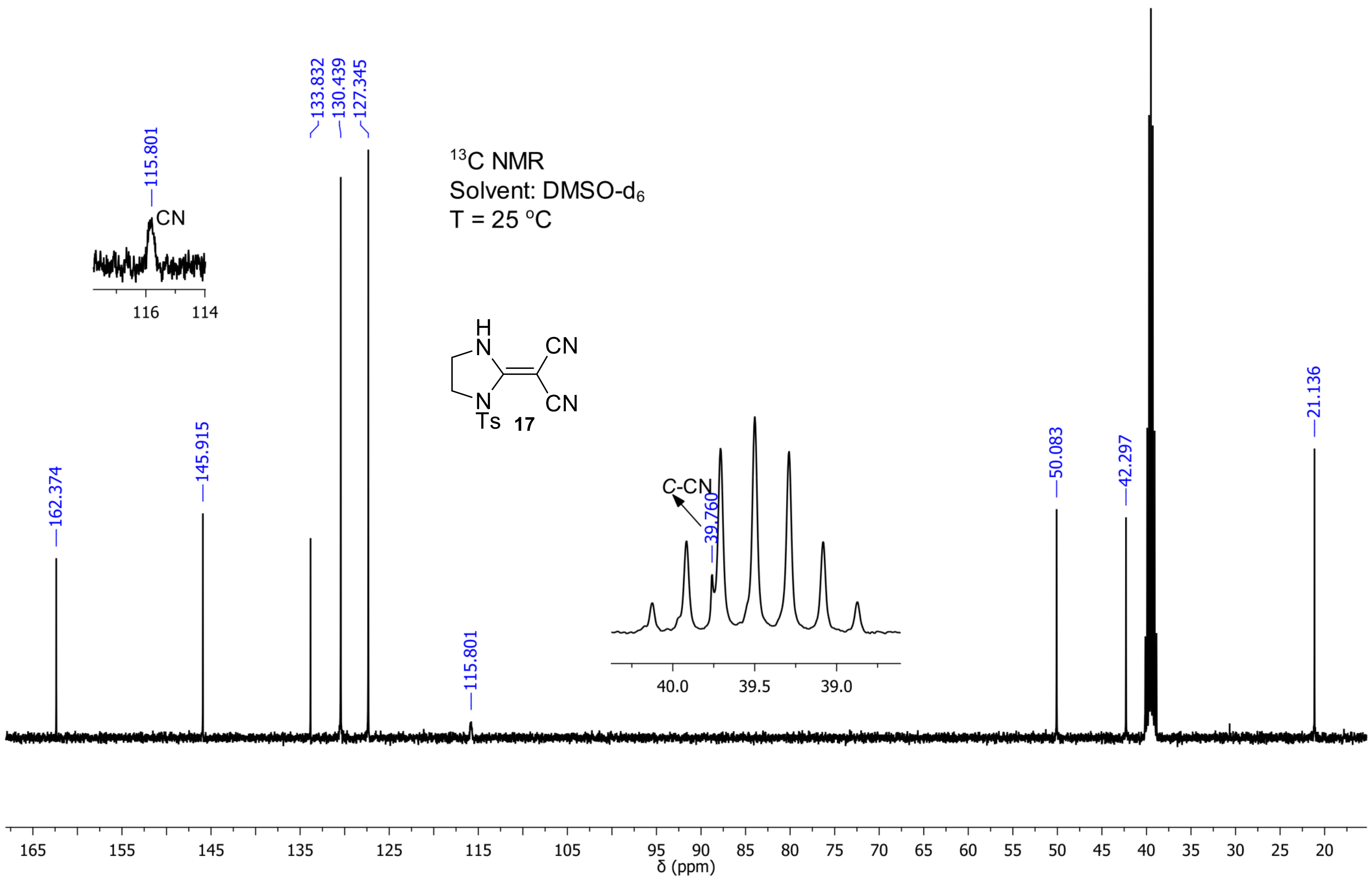
13C NMR
Solvent: DMSO-d6
T = 25 °C
H
N
CN
N
CN
Ts
17
CN
C-CN
162.374
145.915
133.832
130.439
127.345
115.801
50.083
42.297
39.760
21.136
116
114
40.0
39.5
39.0
165
155
145
135
125
115
105
95
90
85
80
75
70
65
60
55
50
45
40
35
30
25
20
δ (ppm)

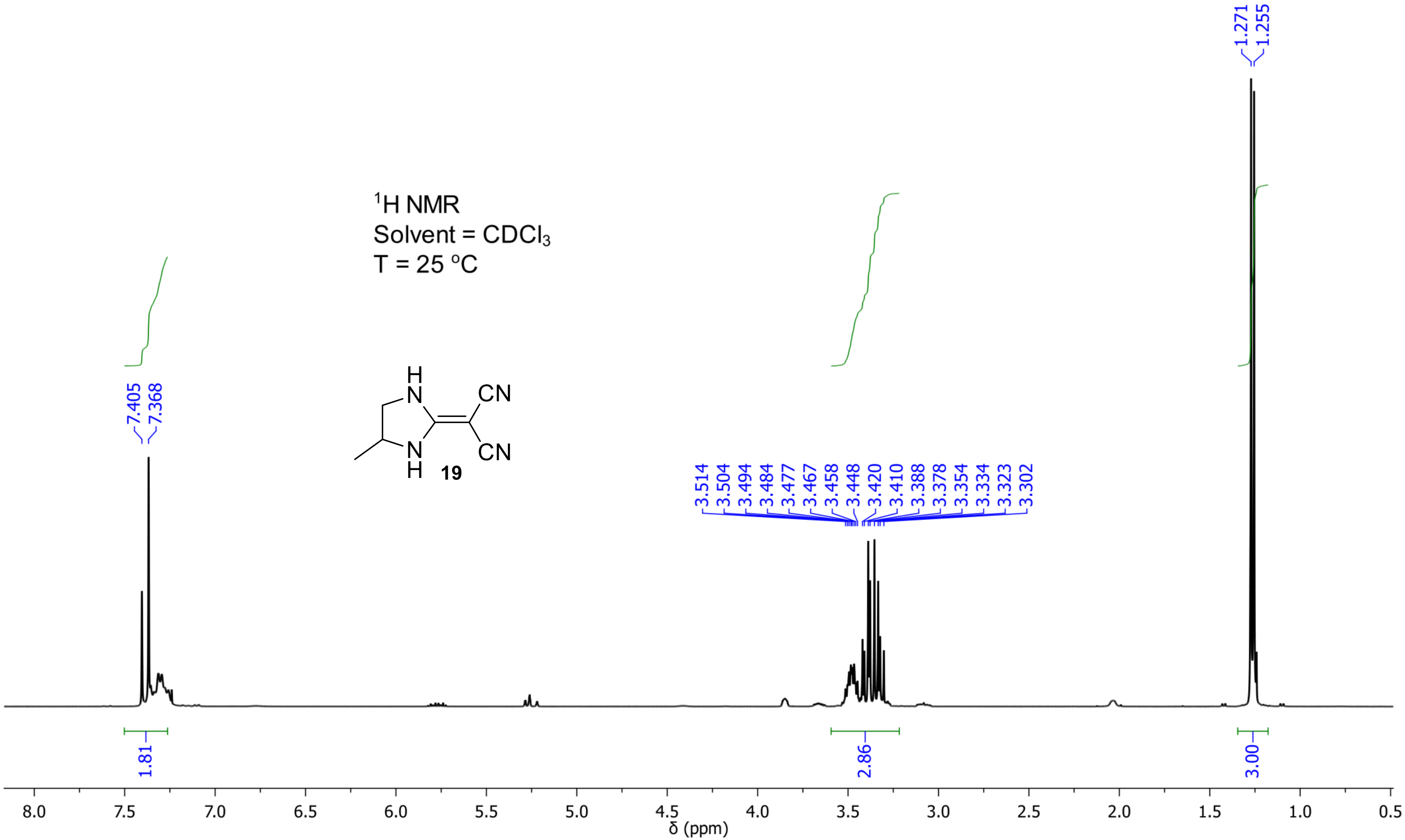

^{1}H NMR
Solvent = CDCl3
T = 25 °C
19
CN
CN
7.405
7.368
3.514
3.504
3.494
3.484
3.477
3.467
3.458
3.448
3.420
3.410
3.388
3.378
3.354
3.334
3.323
3.302
1.271
1.255
1.81
2.86
3.00
8.0
7.5
7.0
6.5
6.0
5.5
5.0
4.5
4.0
3.5
3.0
2.5
2.0
1.5
1.0
0.5
δ (ppm)

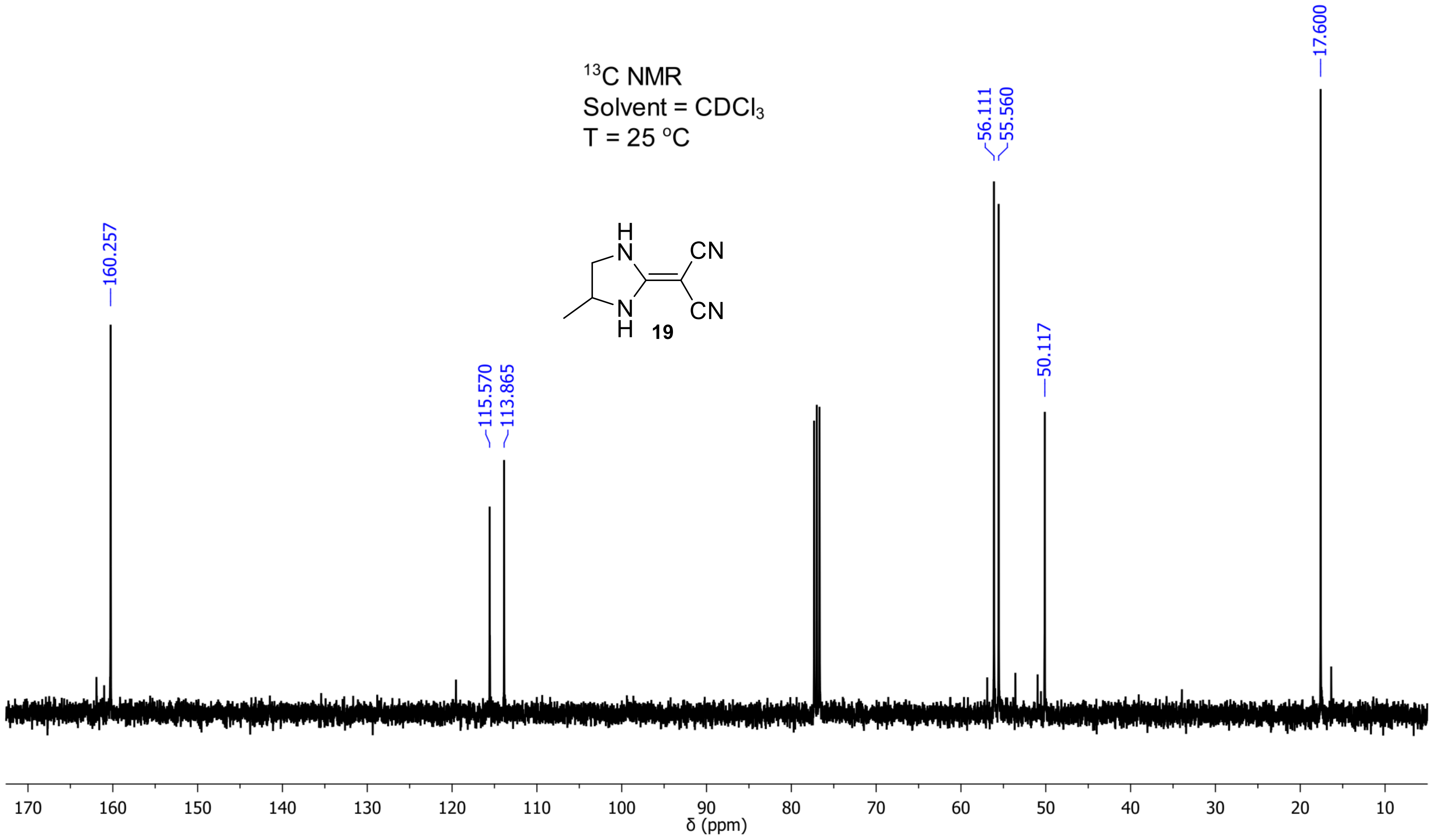

^{13}C NMR
Solvent = $CDCl_3$
T = 25 °C
H
N
CN
N
H
CN
19
160.257
115.570
113.865
56.111
55.560
50.117
17.600
170
160
150
140
130
120
110
100
90
80
70
60
50
40
30
20
10
δ (ppm)

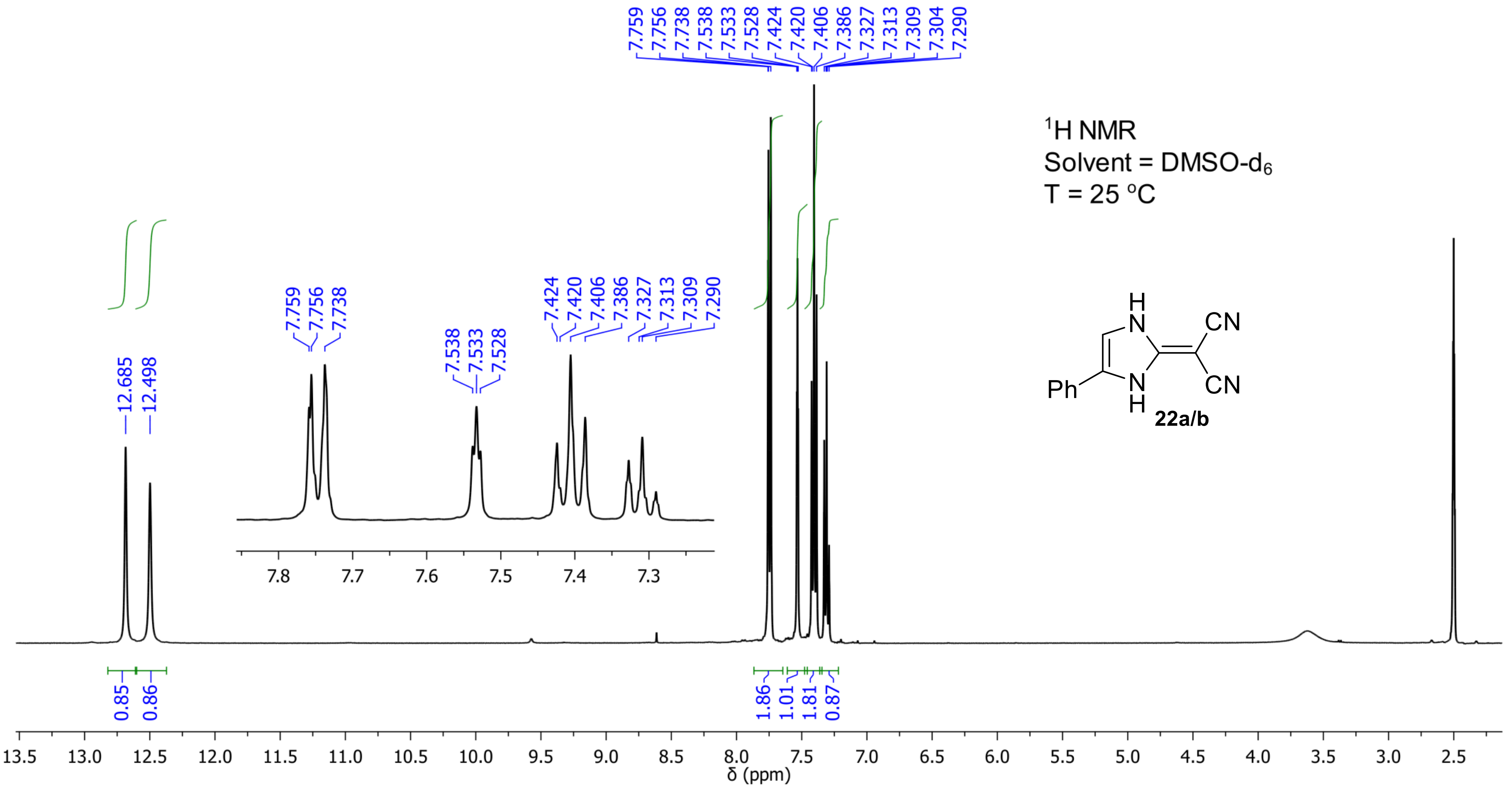

7.759
7.756
7.738
7.538
7.533
7.528
7.424
7.420
7.406
7.386
7.327
7.313
7.309
7.304
7.290
12.685
12.498
0.85
0.86
1.86
1.01
1.81
0.87
^{1}H NMR
Solvent = DMSO-d6
T = 25 °C
Ph
H
N
N
H
CN
CN
22a/b
δ (ppm)

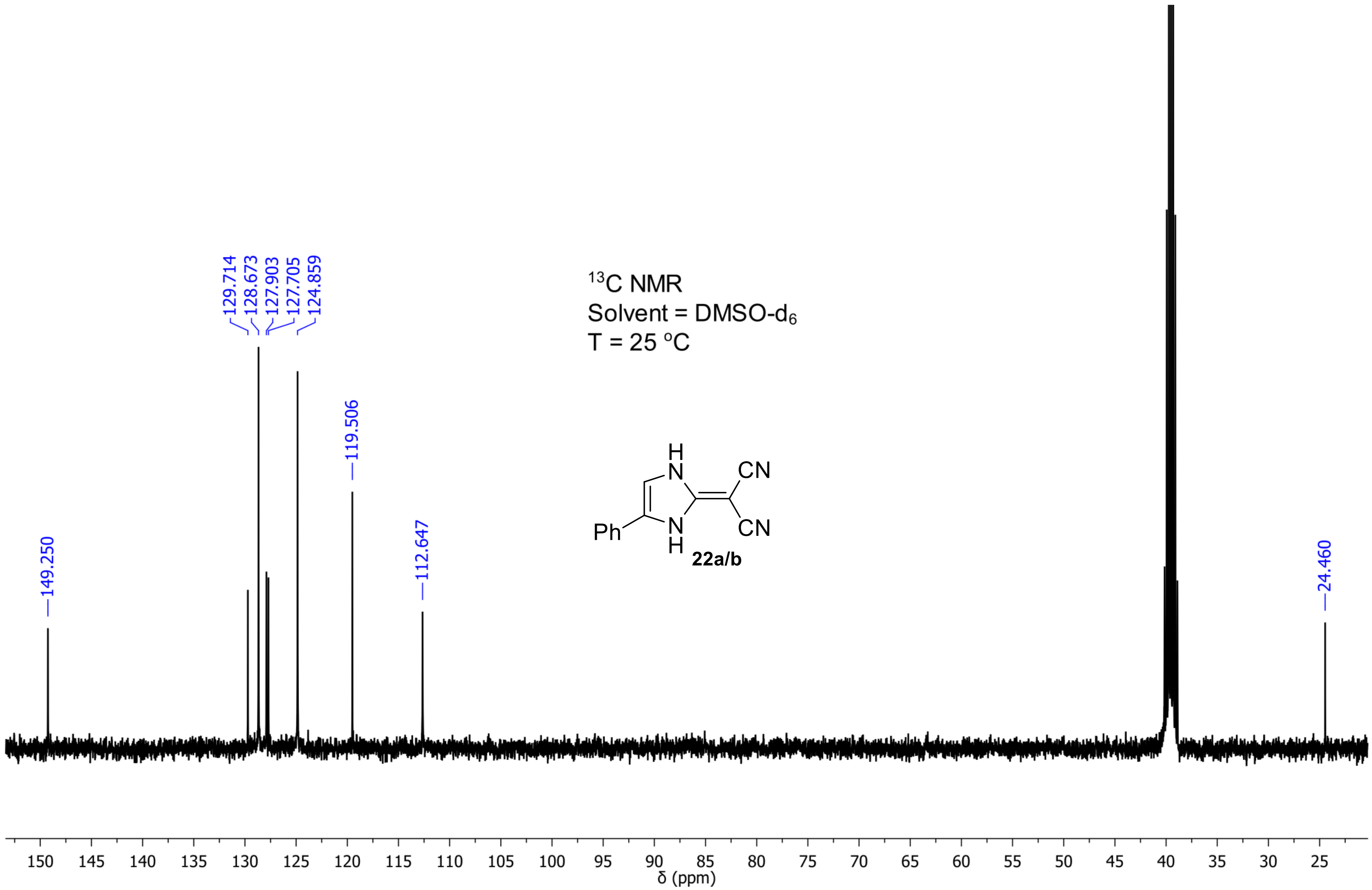

^{13}C NMR
Solvent = DMSO-d_6
T = 25 °C
129.714
128.673
127.903
127.705
124.859
119.506
112.647
149.250
24.460
CN
CN
Ph
H
N
N
H
22a/b
150 145 140 135 130 125 120 115 110 105 100 95 90 85 80 75 70 65 60 55 50 45 40 35 30 25
δ (ppm)

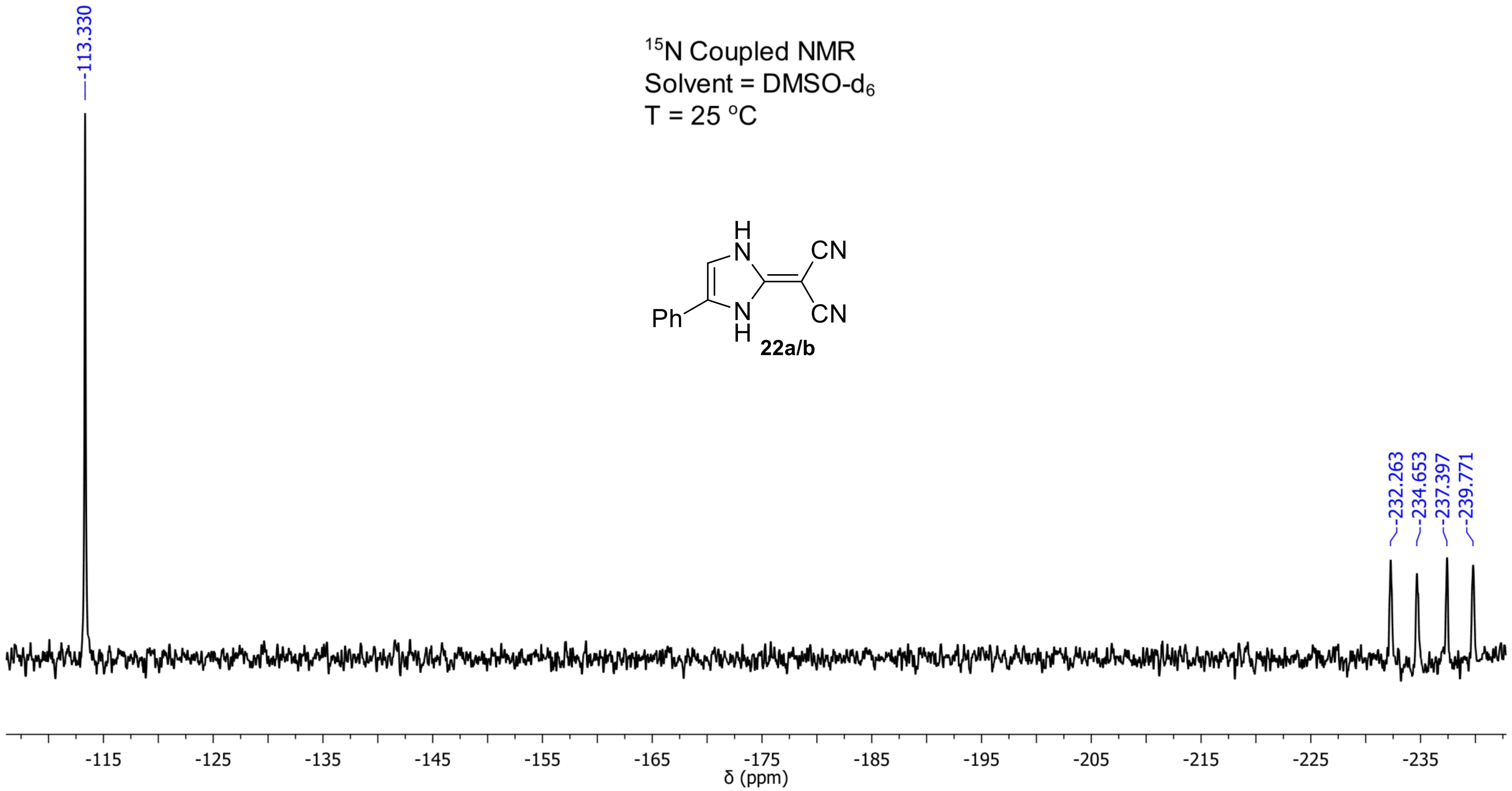

-113.330
^{15}N Coupled NMR
Solvent = DMSO-d_6
T = 25 °C
H
N
CN
Ph
N
H
CN
22a/b
-232.263
-234.653
-237.397
-239.771
-115
-125
-135
-145
-155
-165
-175
-185
-195
-205
-215
-225
-235
δ (ppm)

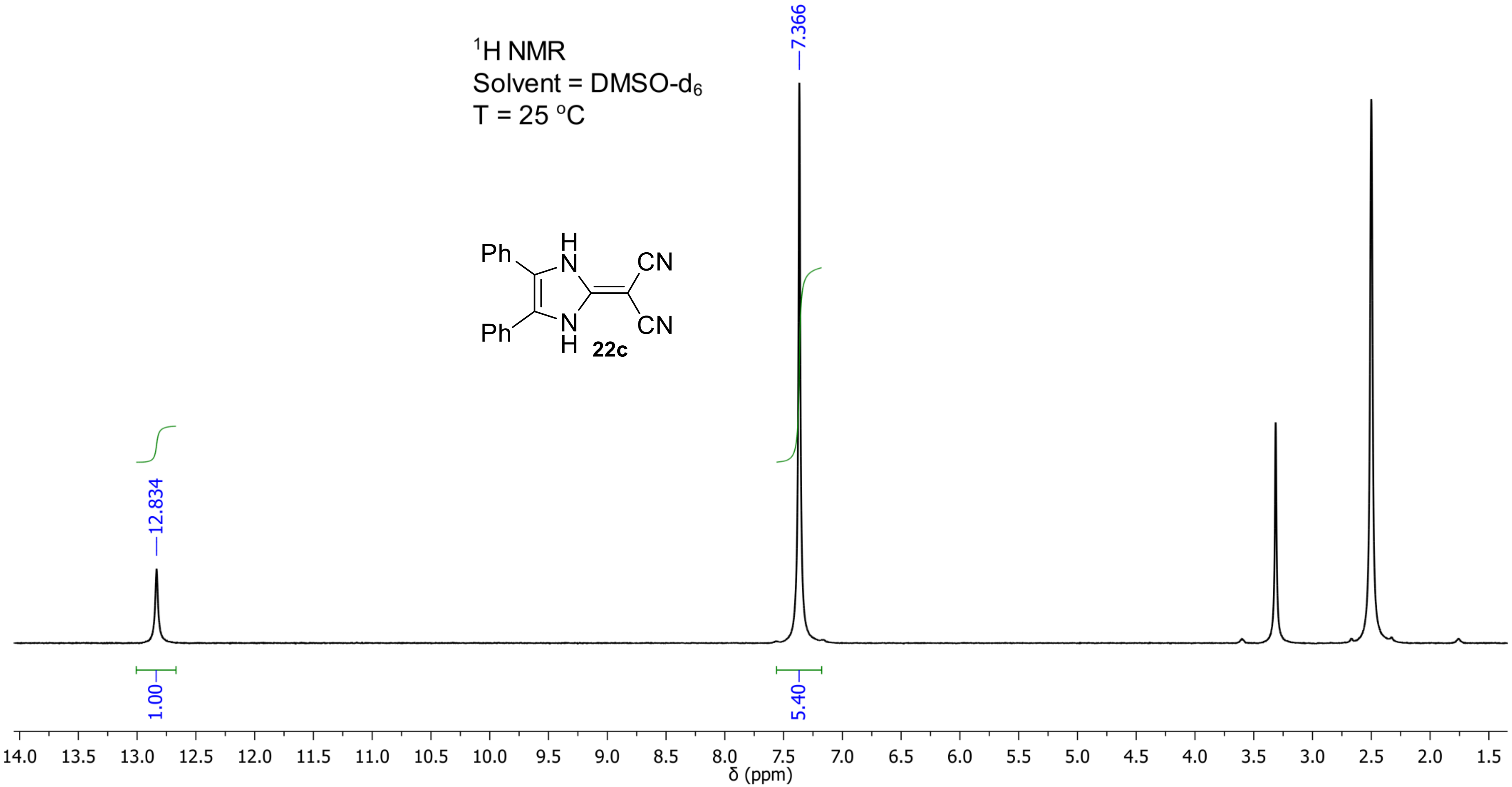
1H NMR
Solvent = DMSO-d6
T = 25 °C
Ph
Ph
H
N
N
H
CN
CN
22c
7.366
12.834
1.00
5.40
14.0 13.5 13.0 12.5 12.0 11.5 11.0 10.5 10.0 9.5 9.0 8.5 8.0 7.5 7.0 6.5 6.0 5.5 5.0 4.5 4.0 3.5 3.0 2.5 2.0 1.5
δ (ppm)

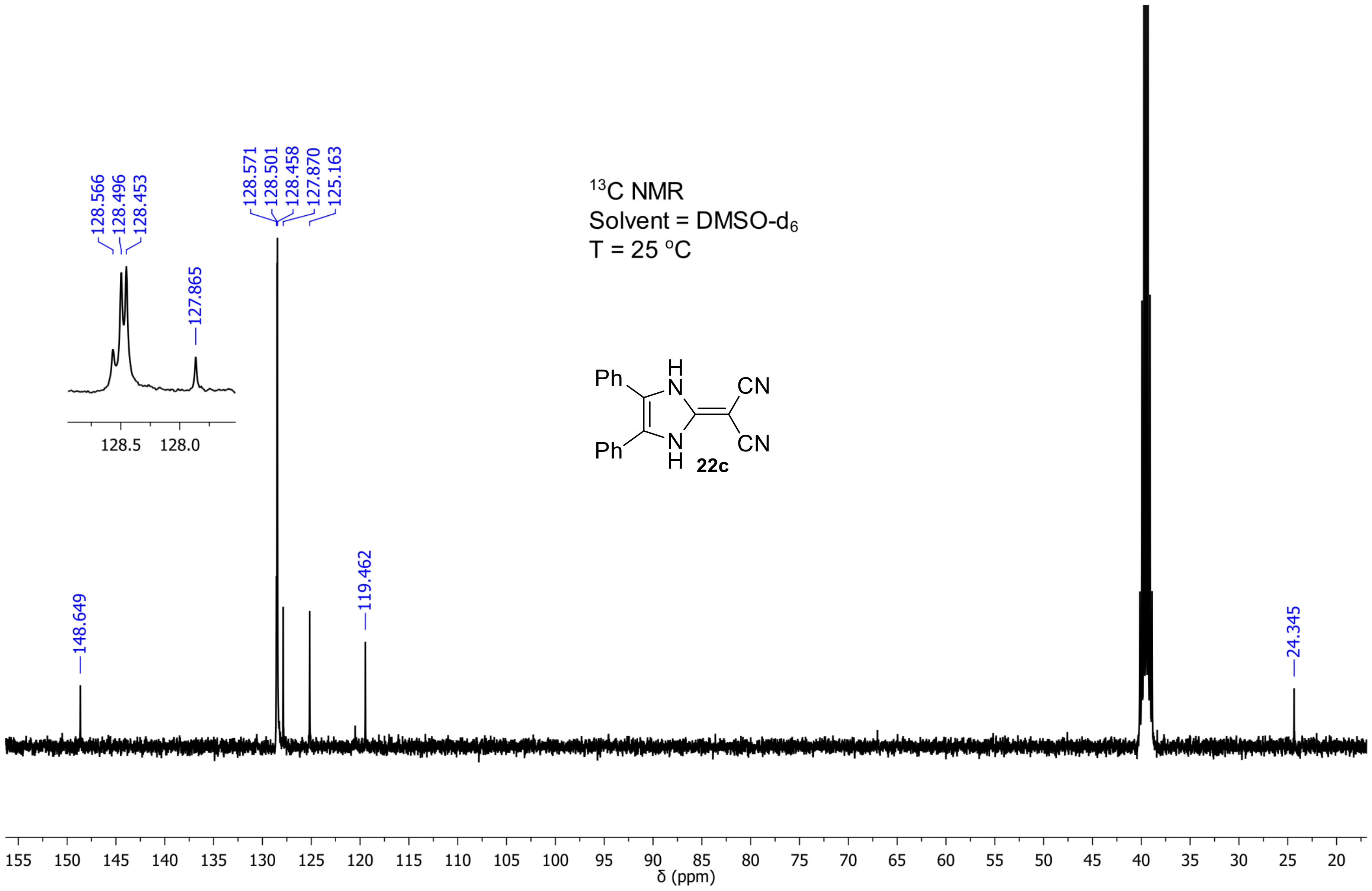

13C NMR
Solvent = DMSO-d6
T = 25 oC
22c
Ph
Ph
H
N
N
H
CN
CN
148.649
128.571
128.501
128.458
127.870
125.163
119.462
24.345
128.566
128.496
128.453
127.865
128.5
128.0
155
150
145
140
135
130
125
120
115
110
105
100
95
90
85
80
75
70
65
60
55
50
45
40
35
30
25
20
δ (ppm)

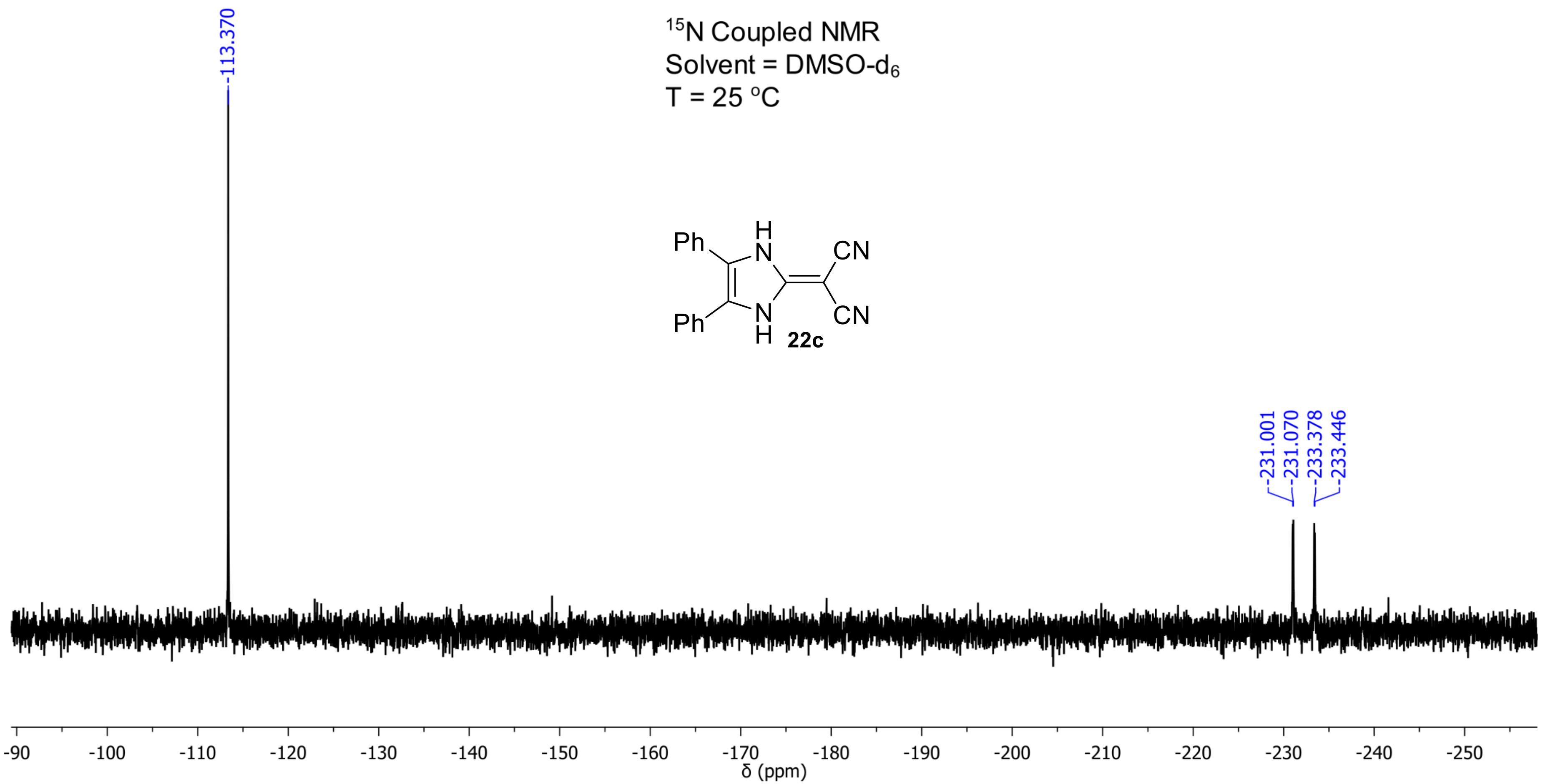
-113.370
^{15}N Coupled NMR
Solvent = DMSO-d6
T = 25 °C
Ph
Ph
H
N
N
H
CN
CN
22c
-231.001
-231.070
-233.378
-233.446
-90
-100
-110
-120
-130
-140
-150
-160
-170
-180
-190
-200
-210
-220
-230
-240
-250
δ (ppm)

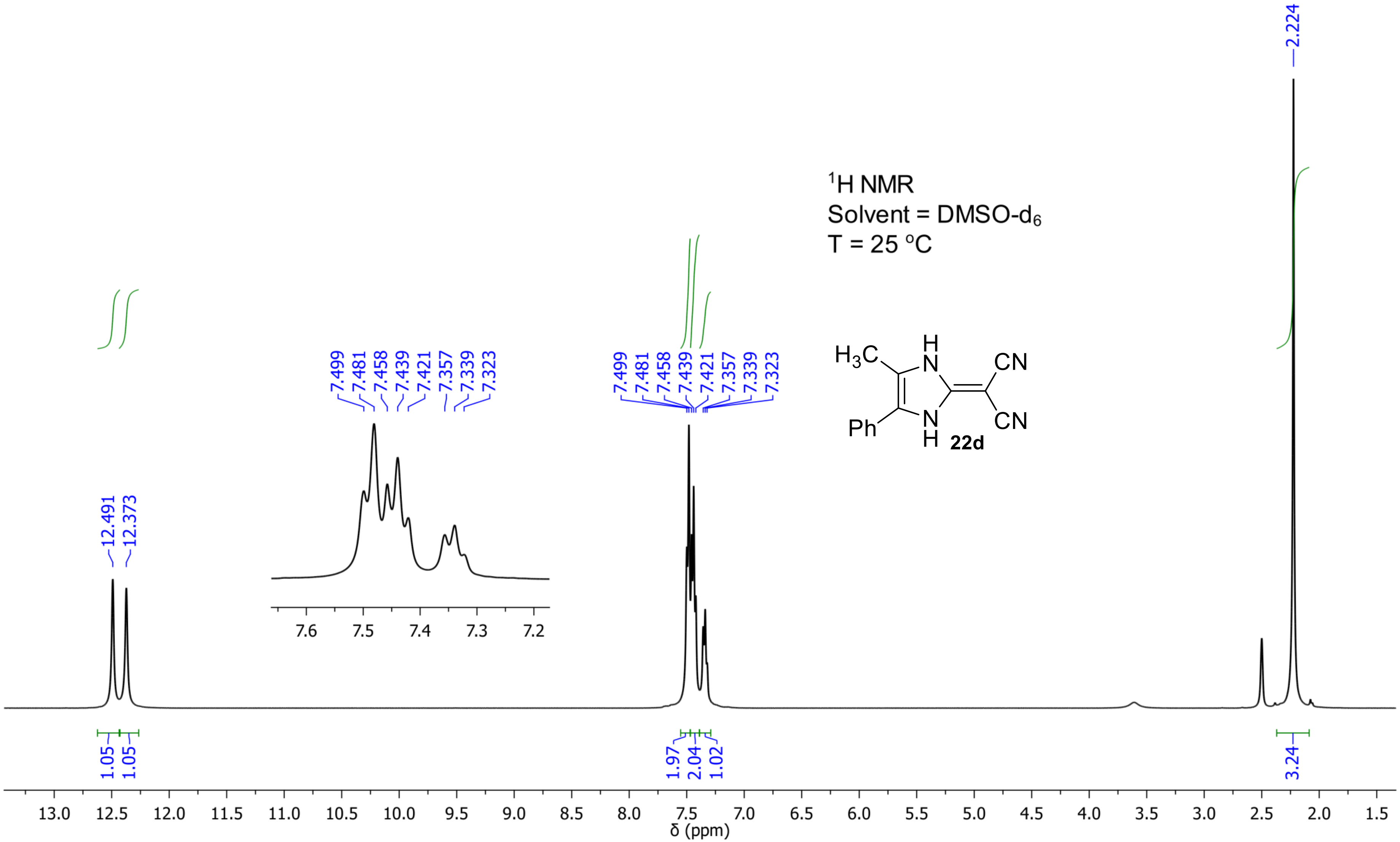

^{1}H NMR
Solvent = DMSO-d$_6$
T = 25 °C
22d
12.491
12.373
7.499
7.481
7.458
7.439
7.421
7.357
7.339
7.323
2.224
1.05
1.05
1.97
2.04
1.02
3.24
δ (ppm)

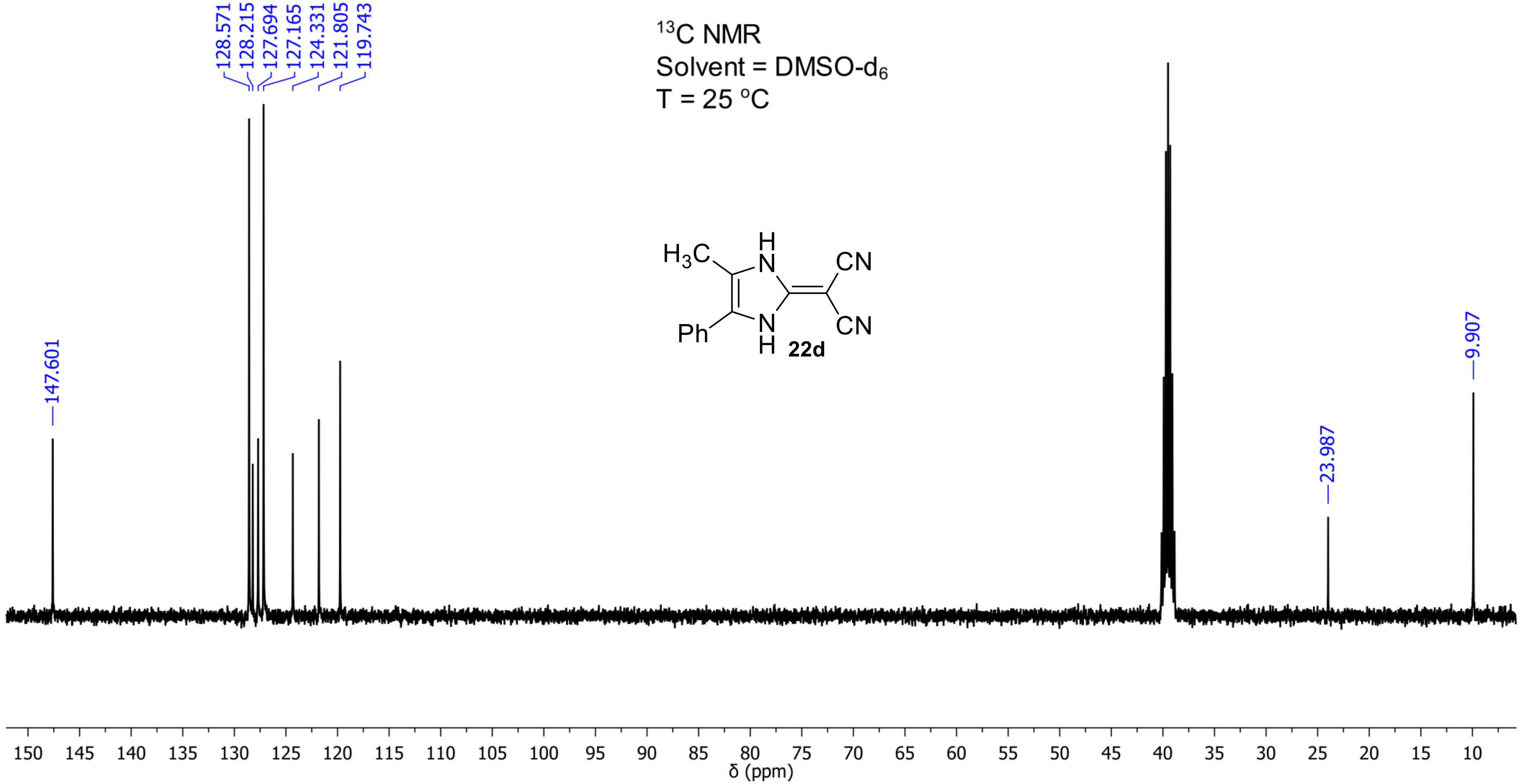

128.571
128.215
127.694
127.165
124.331
121.805
119.743
147.601
23.987
9.907
13C NMR
Solvent = DMSO-d6
T = 25 °C
H3C
H
N
CN
Ph
N
H
CN
22d
150
145
140
135
130
125
120
115
110
105
100
95
90
85
80
75
70
65
60
55
50
45
40
35
30
25
20
15
10
δ (ppm)

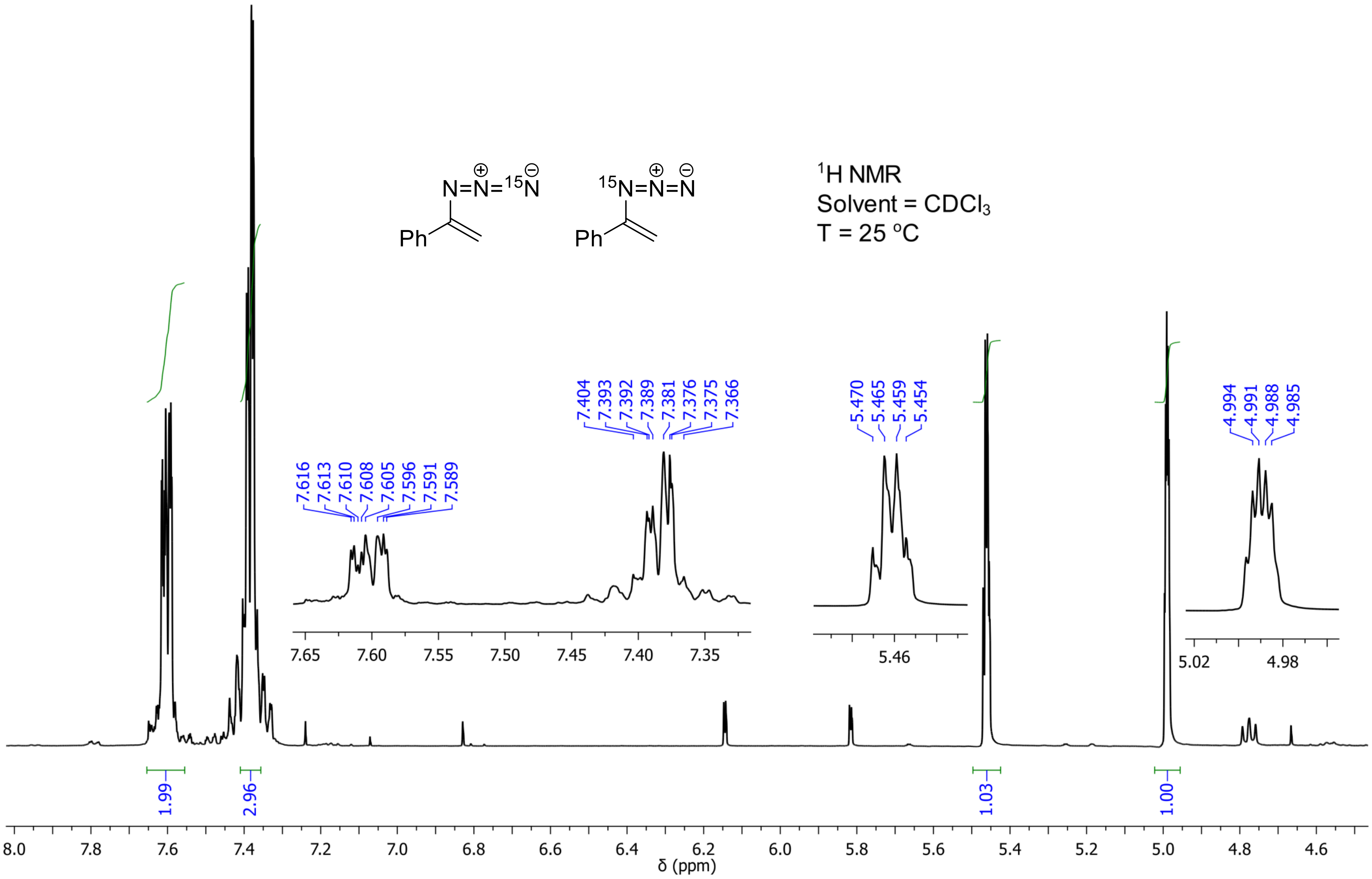

^{1}H NMR
Solvent = CDCl$_3$
T = 25 °C
Ph
N=N=^{15}N
^{15}N=N=N
7.616
7.613
7.610
7.608
7.605
7.596
7.591
7.589
7.404
7.393
7.392
7.389
7.381
7.376
7.375
7.366
5.470
5.465
5.459
5.454
4.994
4.991
4.988
4.985
1.99
2.96
1.03
1.00
δ (ppm)

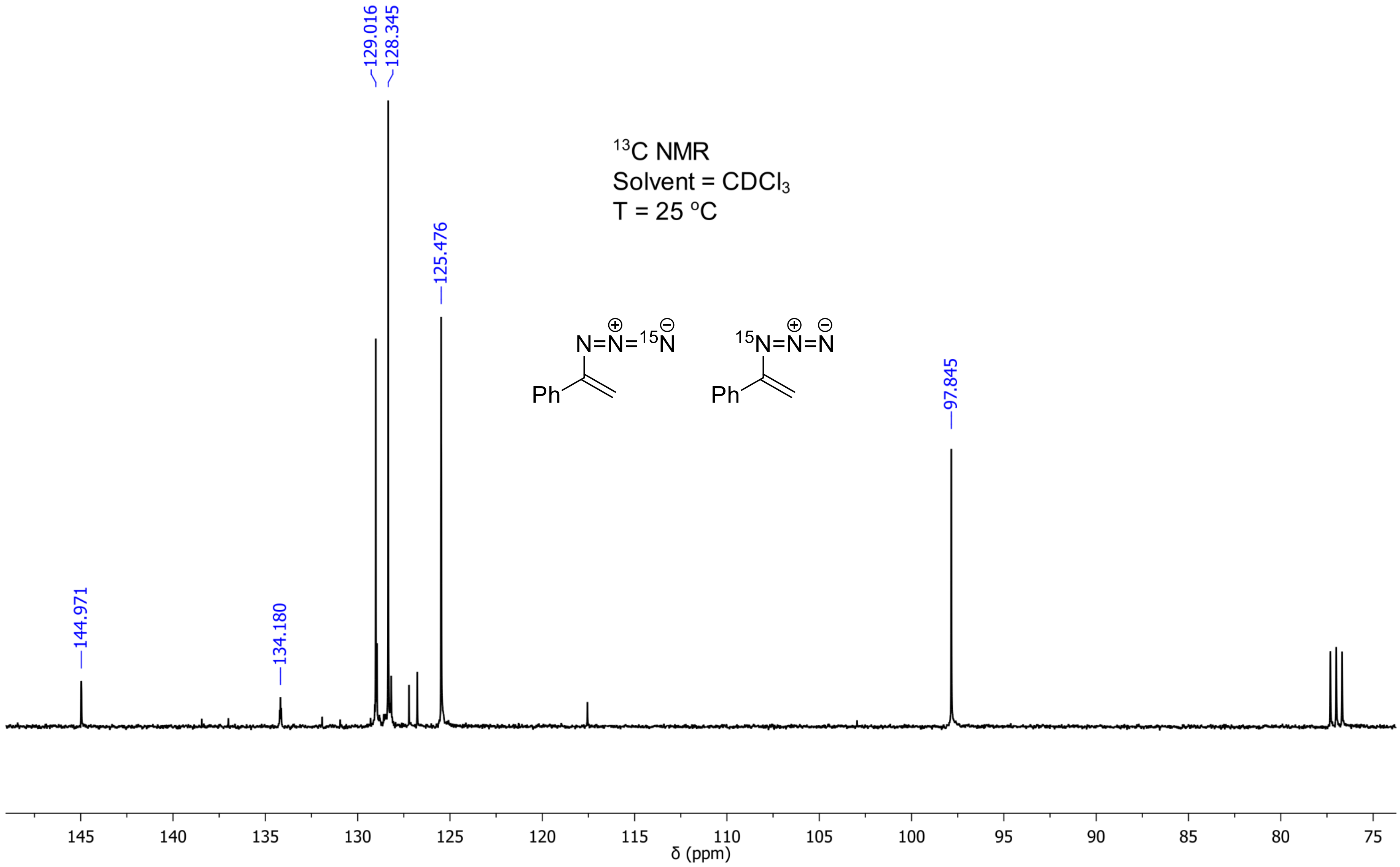
129.016
128.345
125.476
144.971
134.180
97.845
13C NMR
Solvent = CDCl3
T = 25 °C
N=N=15N
15N=N=N
Ph
Ph
145
140
135
130
125
120
115
110
105
100
95
90
85
80
75
δ (ppm)

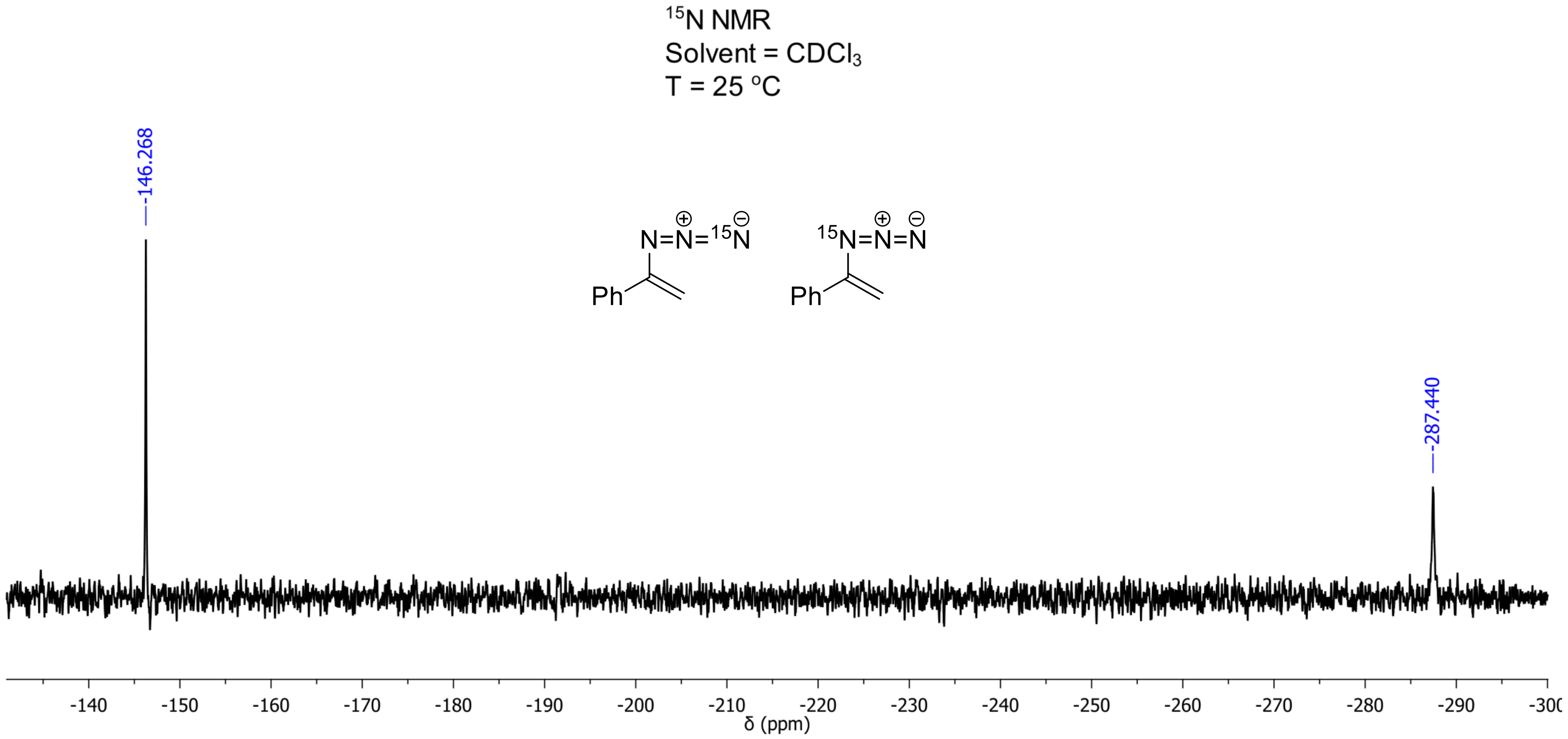

^{15}N NMR
Solvent = $CDCl_3$
T = 25 °C
-146.268
-287.440
Ph
Ph
δ (ppm)

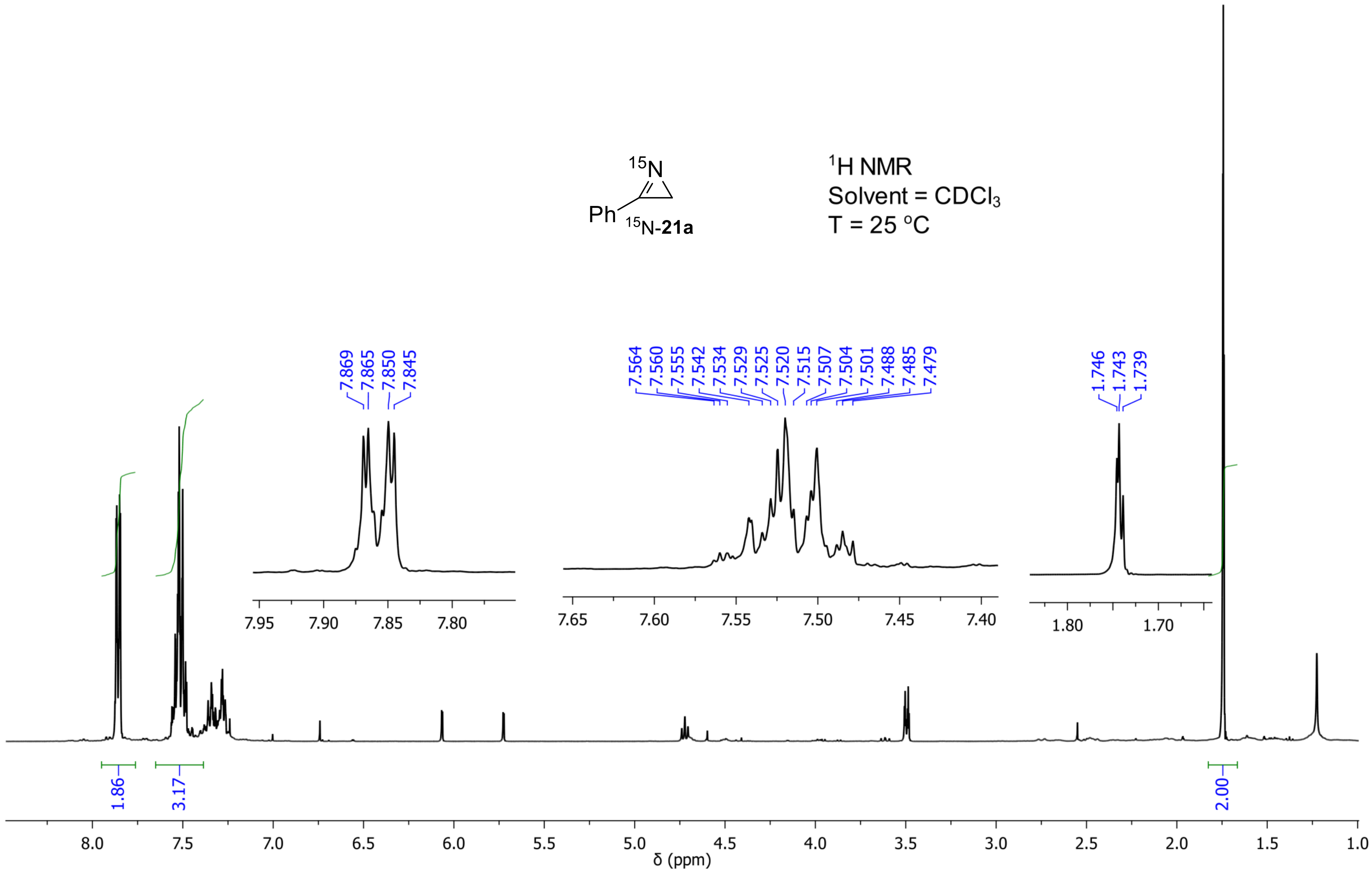
15N
Ph
15N-21a
1H NMR
Solvent = CDCl3
T = 25 oC
7.869
7.865
7.850
7.845
7.564
7.560
7.555
7.542
7.534
7.529
7.525
7.520
7.515
7.507
7.504
7.501
7.488
7.485
7.479
1.746
1.743
1.739
7.95
7.90
7.85
7.80
7.65
7.60
7.55
7.50
7.45
7.40
1.80
1.70
1.86
3.17
2.00
8.0
7.5
7.0
6.5
6.0
5.5
5.0
4.5
4.0
3.5
3.0
2.5
2.0
1.5
1.0
δ (ppm)

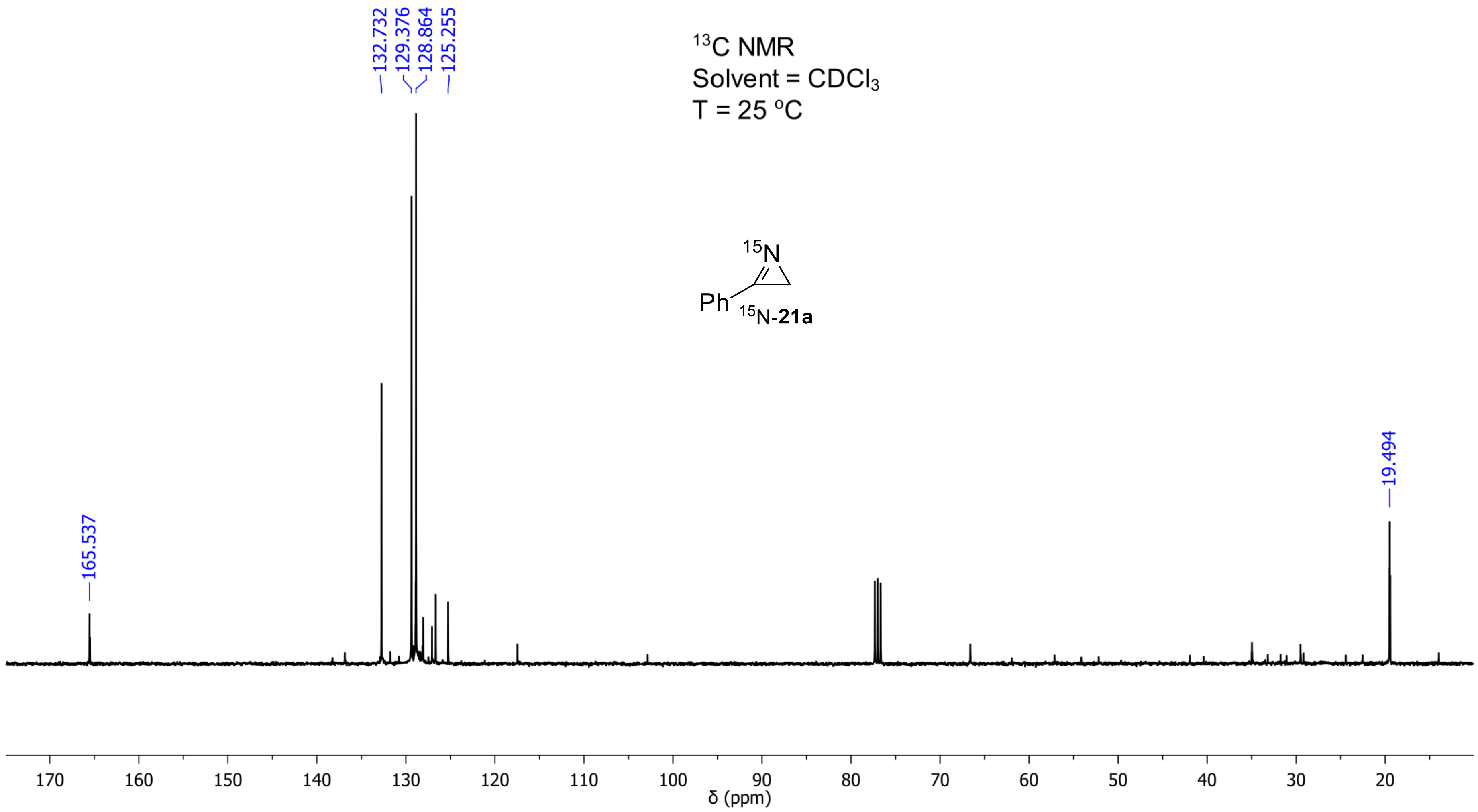
132.732
129.376
128.864
125.255
13C NMR
Solvent = CDCl3
T = 25 oC
15N
Ph
15N-21a
165.537
19.494
170
160
150
140
130
120
110
100
90
80
70
60
50
40
30
20
δ (ppm)

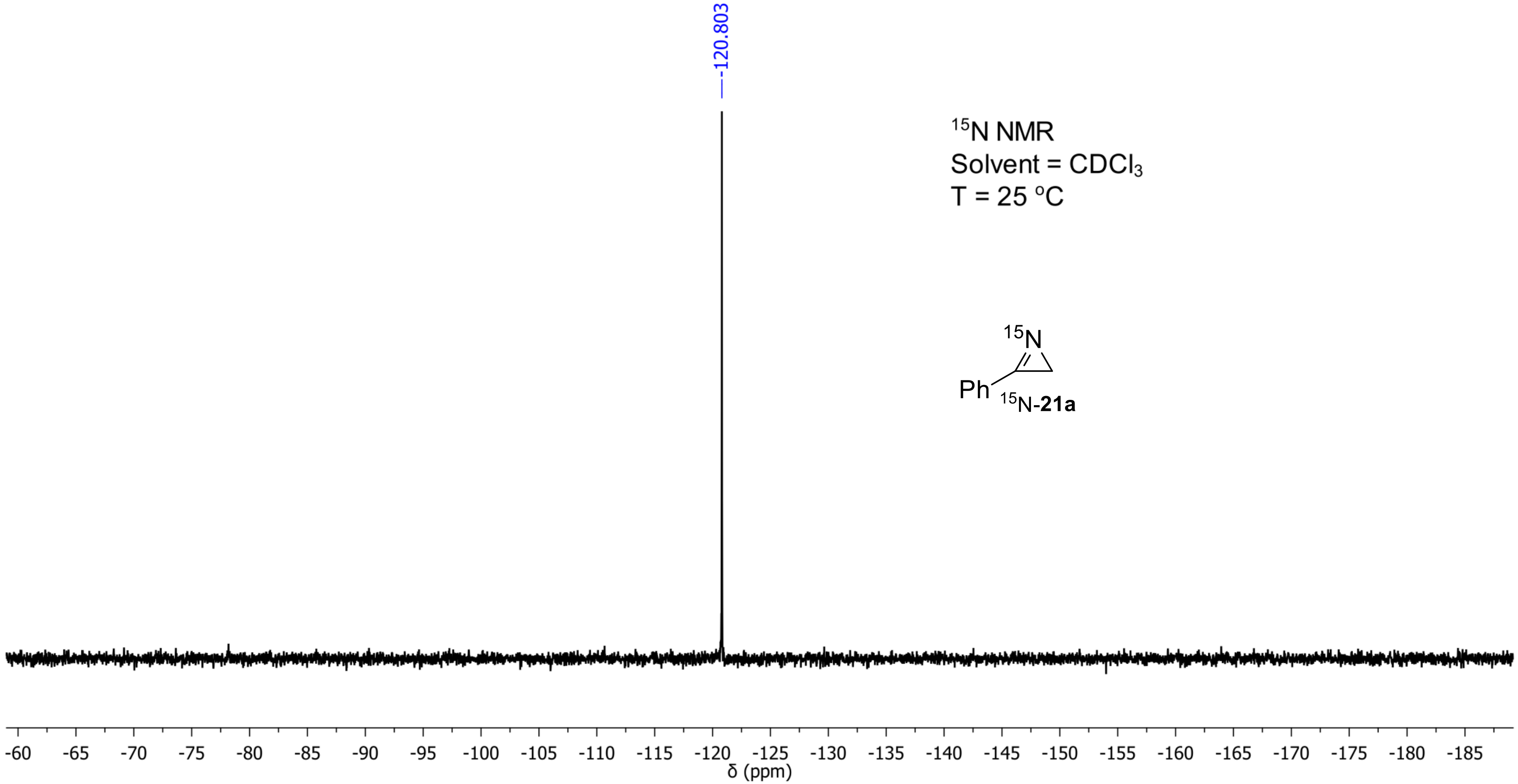

-120.803
^{15}N NMR
Solvent = CDCl$_3$
T = 25 °C
^{15}N
Ph
^{15}N-**21a**
-60
-65
-70
-75
-80
-85
-90
-95
-100
-105
-110
-115
-120
-125
-130
-135
-140
-145
-150
-155
-160
-165
-170
-175
-180
-185
δ (ppm)

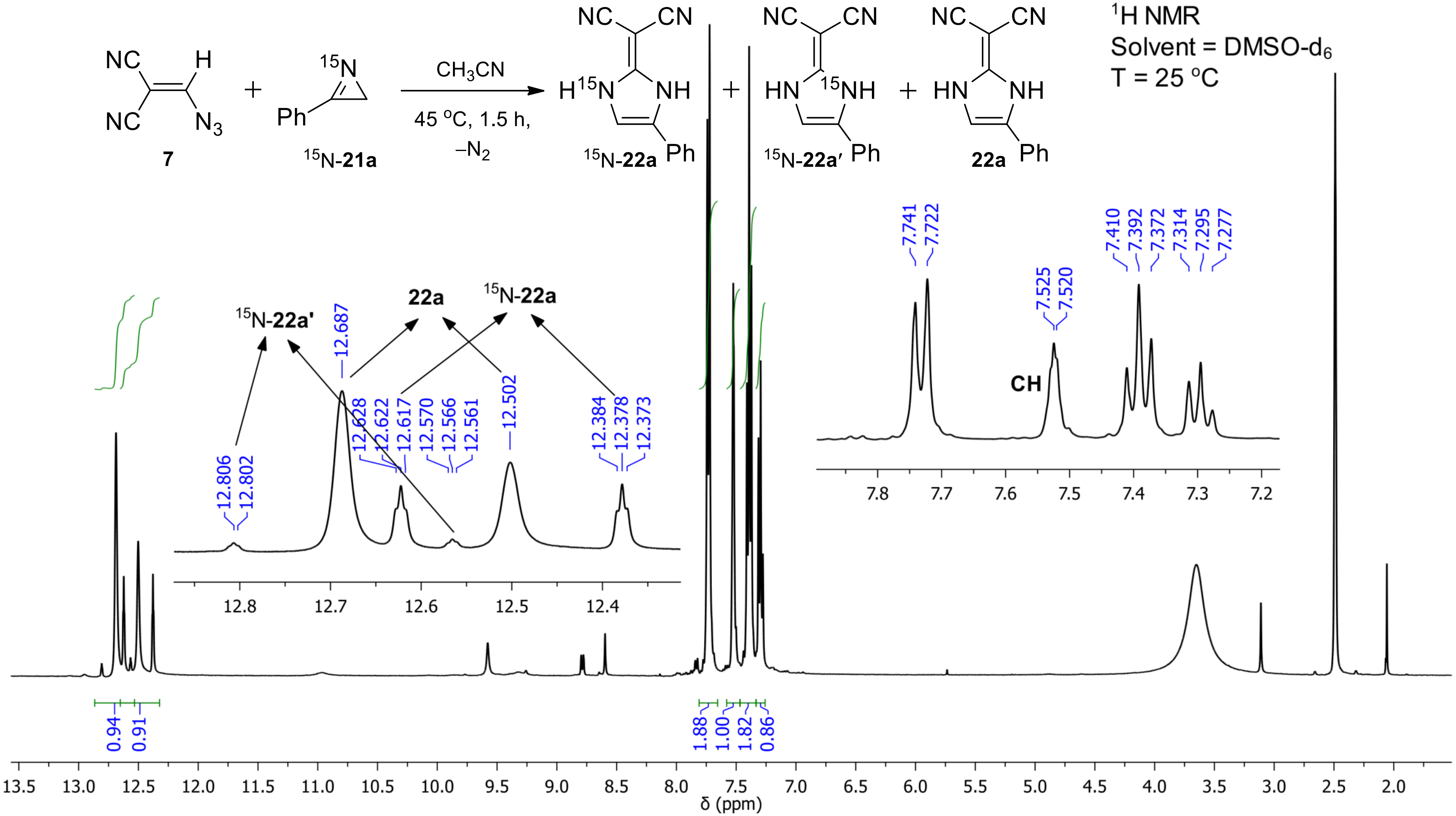

NC
H
N3
7
15N
Ph
15N-21a
CH3CN
45 oC, 1.5 h,
–N2
NC
CN
H15N
NH
Ph
15N-22a
HN
15NH
15N-22a′
22a
1H NMR
Solvent = DMSO-d6
T = 25 oC
15N-22a'
22a
15N-22a
12.806
12.802
12.687
12.628
12.622
12.617
12.570
12.566
12.561
12.502
12.384
12.378
12.373
7.741
7.722
7.525
7.520
7.410
7.392
7.372
7.314
7.295
7.277
CH
0.94
0.91
1.88
1.00
1.82
0.86
δ (ppm)

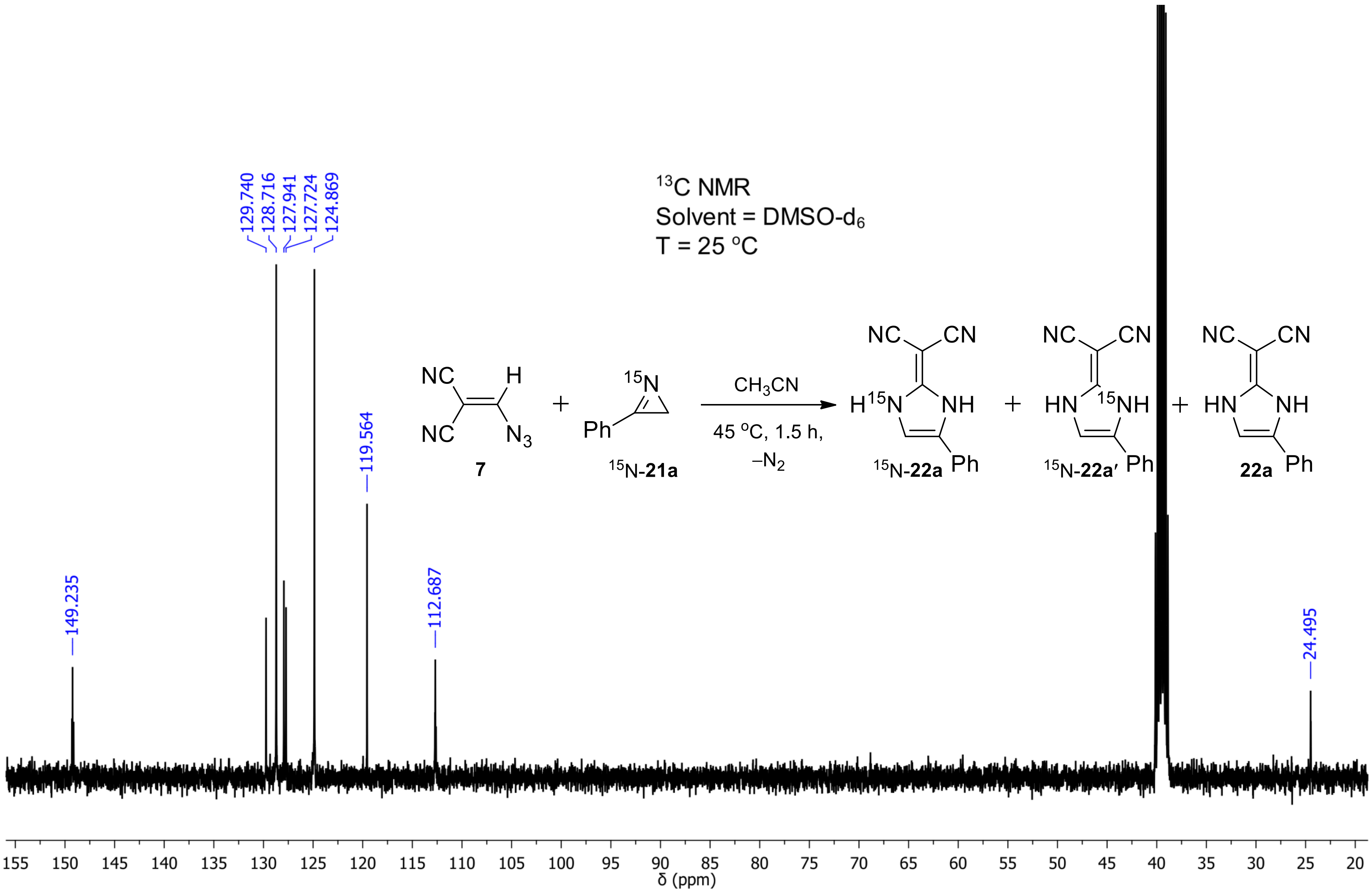

129.740
128.716
127.941
127.724
124.869
119.564
112.687
149.235
24.495
^{13}C NMR
Solvent = DMSO-d$_6$
T = 25 °C
NC
H
NC
N$_3$
7
^{15}N
Ph
^{15}N-21a
CH$_3$CN
45 °C, 1.5 h,
–N$_2$
NC
CN
H^{15}N
NH
^{15}N-22a
Ph
HN
^{15}NH
^{15}N-22a′
HN
NH
22a
155
150
145
140
135
130
125
120
115
110
105
100
95
90
85
80
75
70
65
60
55
50
45
40
35
30
25
20
δ (ppm)

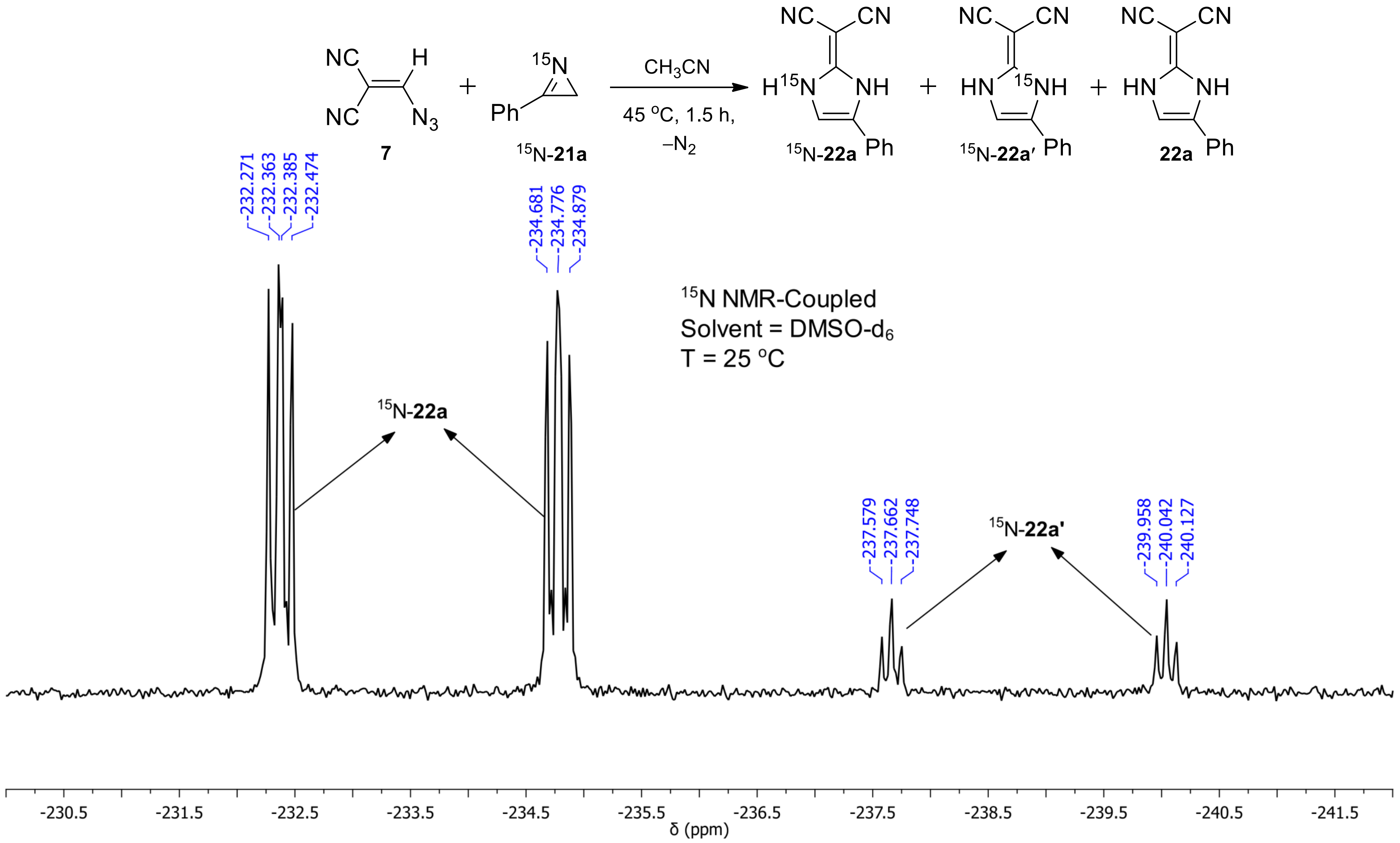
NC
H
NC
N3
7
+
15N
Ph
15N-21a
CH3CN
45 oC, 1.5 h,
–N2
NC
CN
H15N
NH
15N-22a
Ph
+
NC
CN
HN
15NH
15N-22a′
Ph
+
NC
CN
HN
NH
22a
Ph
-232.271
-232.363
-232.385
-232.474
-234.681
-234.776
-234.879
15N NMR-Coupled
Solvent = DMSO-d6
T = 25 oC
15N-22a
-237.579
-237.662
-237.748
15N-22a'
-239.958
-240.042
-240.127
-230.5
-231.5
-232.5
-233.5
-234.5
-235.5
-236.5
-237.5
-238.5
-239.5
-240.5
-241.5
δ (ppm)

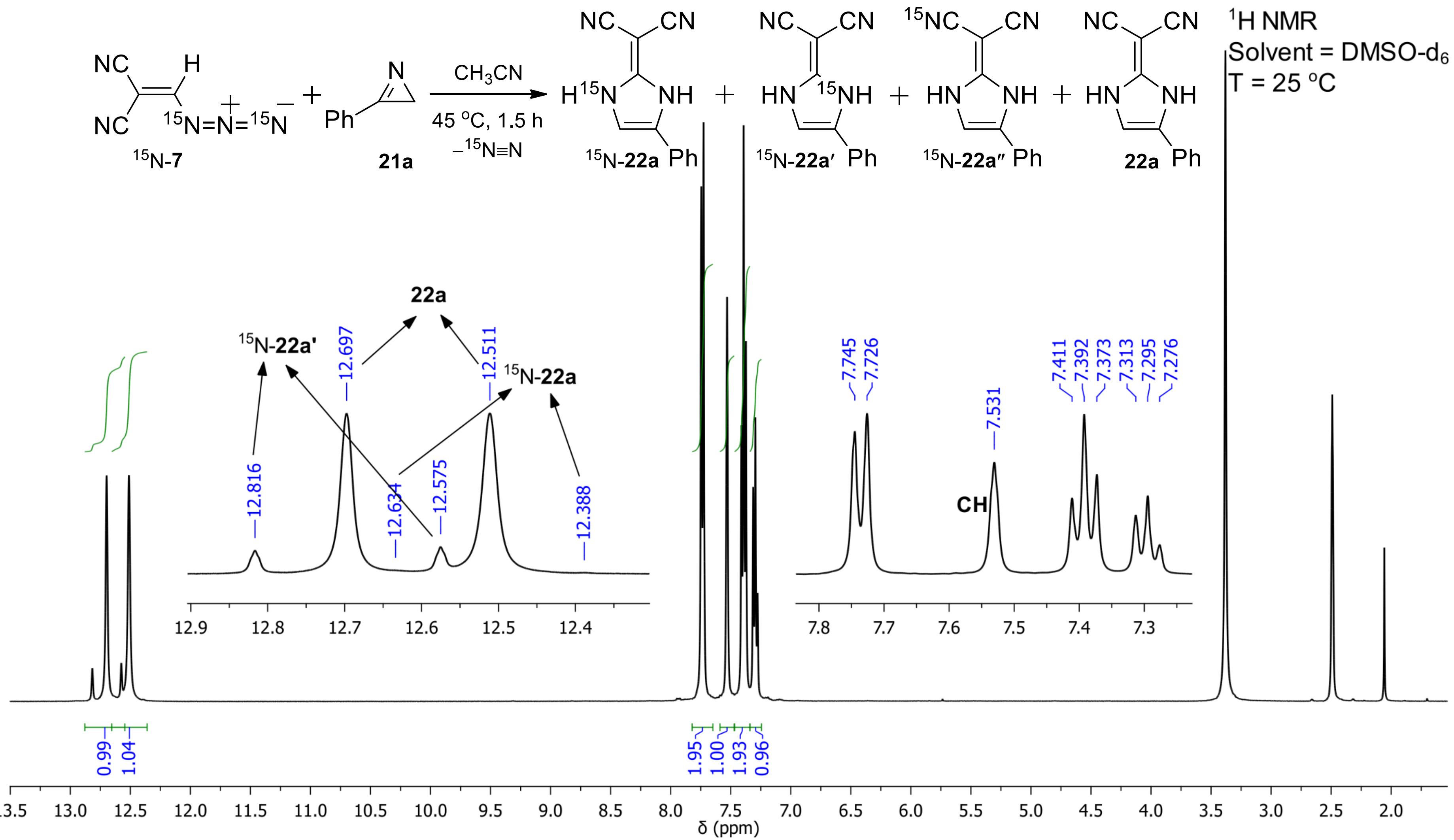
15N-7
21a
CH3CN
45 oC, 1.5 h
–15N≡N
15N-22a
15N-22a′
15N-22a″
22a
1H NMR
Solvent = DMSO-d6
T = 25 oC
22a
15N-22a'
15N-22a
12.816
12.697
12.634
12.575
12.511
12.388
7.745
7.726
7.531
7.411
7.392
7.373
7.313
7.295
7.276
CH
0.99
1.04
1.95
1.00
1.93
0.96
δ (ppm)

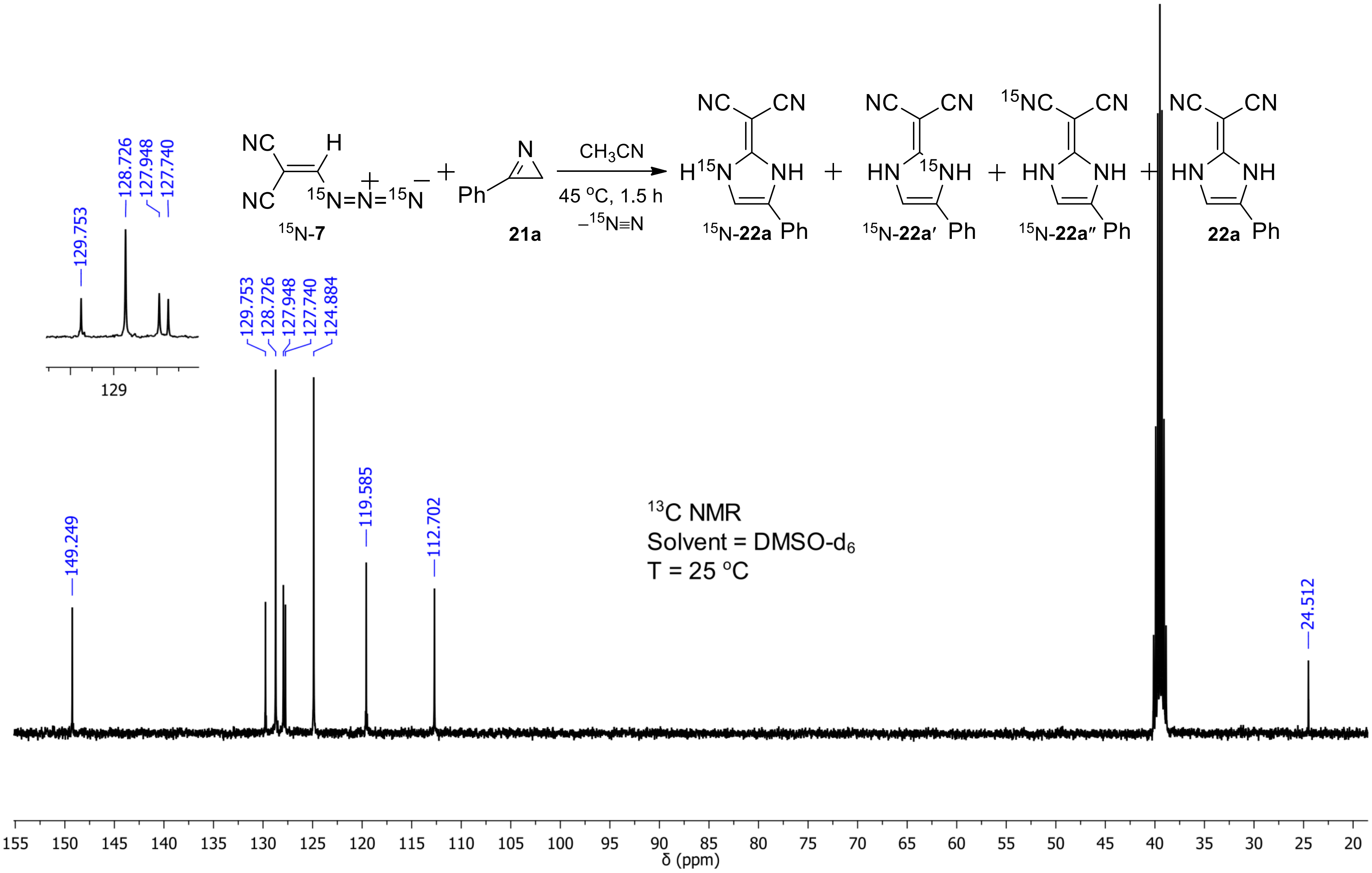
NC
H
NC
$^{15}N=N^{+}=^{15}N^{-}$
^{15}N-7
Ph
N
21a
CH_3CN
45 °C, 1.5 h
$-^{15}N{\equiv}N$
^{15}N-22a
^{15}N-22a′
^{15}N-22a″
22a
^{13}C NMR
Solvent = DMSO-d_6
T = 25 °C
149.249
129.753
128.726
127.948
127.740
124.884
119.585
112.702
24.512
129
δ (ppm)

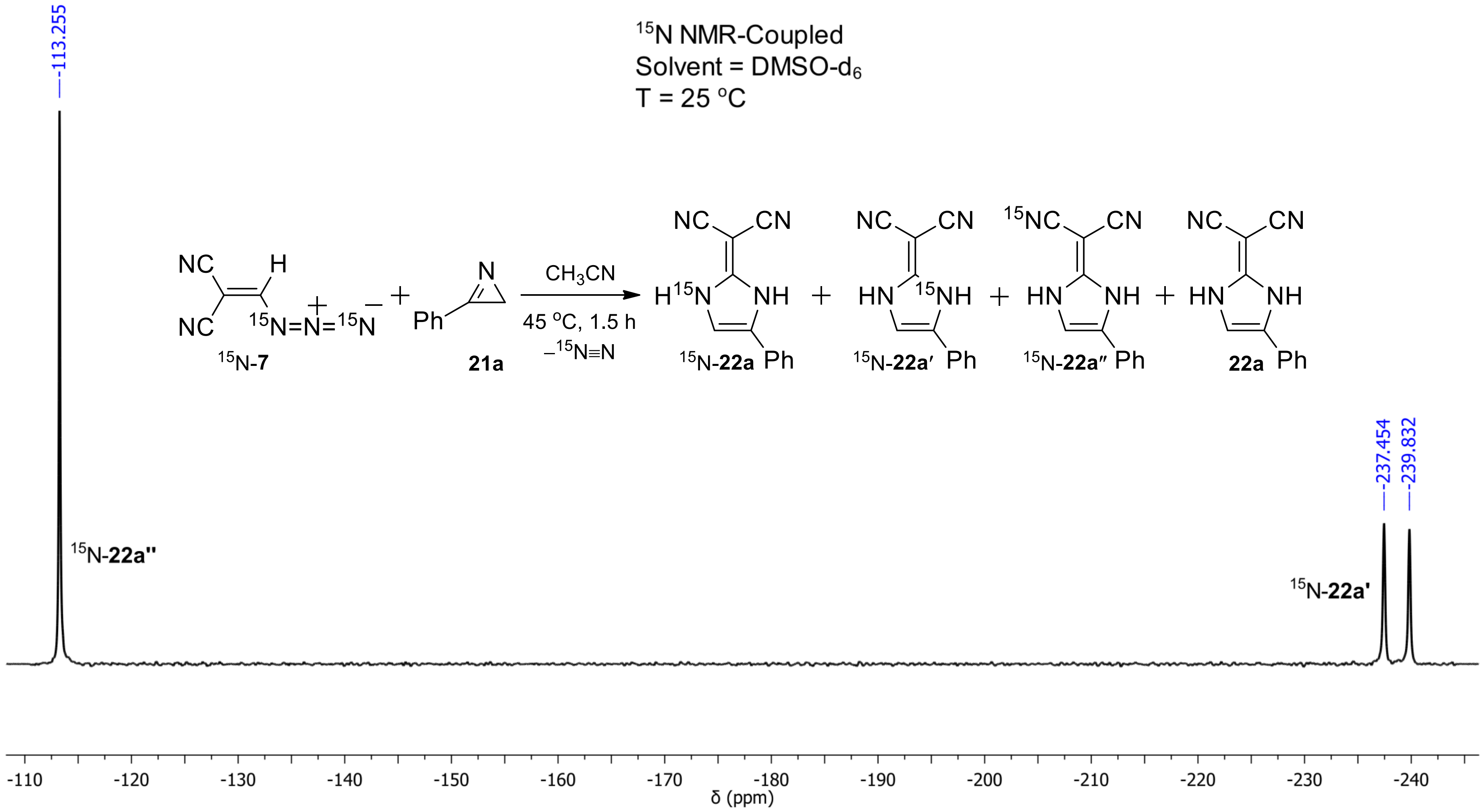

15N NMR-Coupled
Solvent = DMSO-d6
T = 25 °C
NC
H
NC
15N=N=15N
15N-7
Ph
N
21a
CH3CN
45 °C, 1.5 h
–15N≡N
NC
CN
H15N
NH
15N-22a
Ph
HN
15NH
15N-22a′
15NC
15N-22a″
22a
-113.255
15N-22a''
-237.454
-239.832
15N-22a'
-110
-120
-130
-140
-150
-160
-170
-180
-190
-200
-210
-220
-230
-240
δ (ppm)

Intermolecular Interactions in the packing of **13g** and **17**.

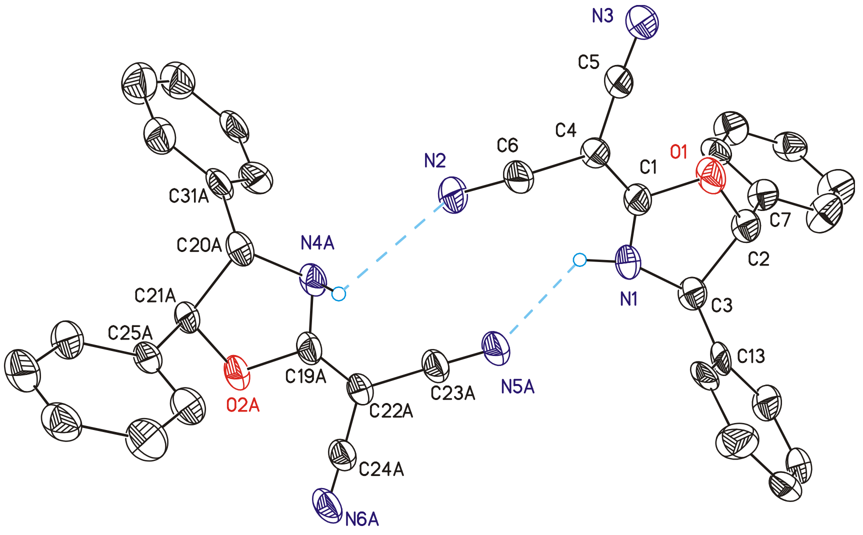

Figure SI1. ORTEP (30 % probability level) of the molecular structure of **13g**, showing the intermolecular hydrogen bonds (blue) and the hence formed dimer. Geometric properties (Å/°): N1···N5A 2.91(3), N1–H1N···N5A 123(18). Symmetry code: (A) 1/2–x, 1/2+y, –1/2+z.

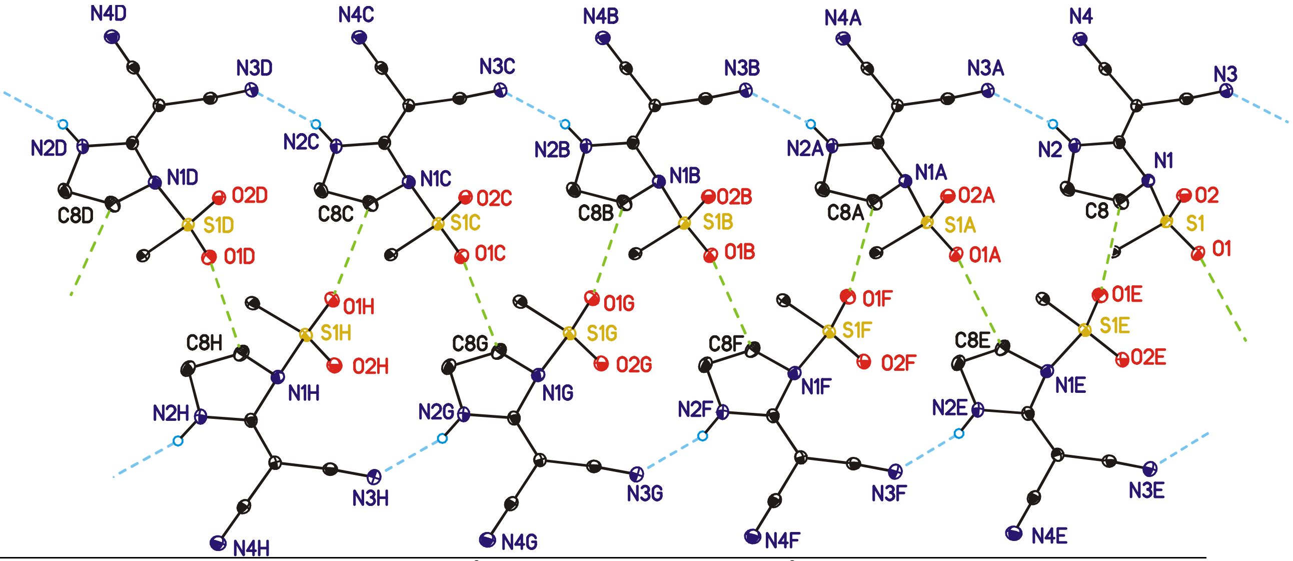

D–H···A	D–H / Å	D···A / Å	D–H···A / °
N2–H2N···N3	0.86(2)	2.8674(19)	160(2)
N6–H6N···N7	0.86(2)	2.8679(18)	160.5(19)
C8–H8A···O1	0.97	3.112(2)	130
C23–H23B···O3	0.97	3.1931(18)	139

Figure SI2. ORTEP (30 % probability level) of the molecular structure of **13g**, showing the intermolecular hydrogen bonds (blue) and non-classic (C–H···O) hydrogen bonds

(green), resulting chains along the crystallographic *b*-axis. The second molecule of the asymmetric unit forms an equal pattern, which is omitted herein. The *p*-tolyl fragment has been reduced to its ipso carbon atom for clarity. Symmetry code: (A) x, y+1, z; (B) x, y+2, z; (C) x, y+3, z; (D) x, y+4, z; (E) –x, y+1/2, 1/2–z; (F) –x, y+3/2, 1/2–z; (G) –x, y+5/2, 1/2–z; (H) –x, y+7/2, 1/2–z.

References

[S-1] G. M. Sheldrick, *Acta Crystallogr.* **1990**, *A46*, 467−473.

[S-2] G. M. Sheldrick, *Program for Crystal Structure Refinement*; University of Göttingen, Göttingen, Germany, **1997**.

[S-3] G. M. Sheldrick, *Acta Crystallogr*. **2008**, *A24*, 112−122.

[S-4] L. J. Farrugia, *J. Appl. Crystallogr*. **2012**, *45*, 849−854.

[S-5] H. D. Flack, *Acta Crystallogr., Sect. A* **1983**, *39*, 876.

[S-6] A. D. Josey, C. L. Dickinson, K. C. Dewhirst, B. C. McKusick, *J. Org. Chem.* **1967**, *32*, 1941–1944.

[S-7] H. Schubert, M. Regitz, *Synthesis*, **1982**, 149–151.

[S-8] L. G. Chanu, O. M. Singh, S. H. Jang, S. G. Lee, *Bull. Korean Chem. Soc.* **2010**, *31*, 859–862.

[S-9] J. A. Ciaccio, A. L. Drahus, R. M. Meis, C. T. Tingle, M. Smrtka, R. Geneste, *Synth. Commun.* **2003**, *33*, 2135–2143.

[S-10] M. Bakavoli, H. Beyzaie, M. Rahimizadeh, H. Eshghi, R. Takjoo, *Molecules* **2009**, *14*, 4849–4857.

[S-11] N. Jiang, J. Fan, T. Liu, J. Cao, B. Qiao, J. Wang, P. Gao, X. Peng, *Chem. Commun.* **2013**, *49*, 10620–10622.

[S-12] a) X. Zhang, S. K. Sarkar, G. K. Weragoda, S. Rajam, B. S. Ault, A. D. Gudmundsdottir, *J. Org. Chem.* **2014**, *79*, 653–663. b) b) H. Yang, Y. Li, M. Jiang, J. Wang, H. Fu, *Chem. Eur. J.* **2011**, *17*, 5652–5660. c) K. Isomura, M. Okada, H. Taniguchi, *Tetrahedron Lett.* **1969**, *10*, 4073–4076. d) K. Isomura, H. Taniguchi, *Org. Magn. Reson.* **1977**, *9*, 559–562.

[S-13] E. Orton, S. T. Collins, G. C. Pimentel, *J. Phys. Chem.* **1986**, *90*, 6139–6143.

[S-14] D. Šišak, L. B. McCusker, A. Buckl, G. Wuitschik, Y. L. Wu, W. B. Schweizer, J. D. Dunitz, *Chem. Eur. J.* **2010**, *16*, 7224–7230.

[S-15] R. A. Carboni, *Org. Synth.* **1959**, *39*, 64–67.

[S-16] a) S. Trofimenko, E. L. Little, Jr., H. F. Mower, *J. Org. Chem.* **1962**, *27*, 433–438. b) S. Trofimenko, *J. Org. Chem.* **1963**, *28*, 2755–2758.
